# Cancer chemoprevention: signaling pathways and strategic approaches

**DOI:** 10.1038/s41392-025-02167-1

**Published:** 2025-04-18

**Authors:** Junling Ren, Guangli Yan, Le Yang, Ling Kong, Yu Guan, Hui Sun, Chang Liu, Lei Liu, Ying Han, Xijun Wang

**Affiliations:** 1 State key Laboratory of Integration and Innovation of Classic Formula and Modern Chinese Medicine, National Chinmedomics Research Center, National TCM Key Laboratory of Serum Pharmacochemistry, Metabolomics Laboratory, Department of Pharmaceutical Analysis, Heilongjiang University of Chinese Medicine, Heping Road 24, Harbin, 150040 China; 2 State Key Laboratory of Dampness Syndrome, The Second Affiliated Hospital Guangzhou University of Chinese Medicine, Dade Road 111, Guangzhou, China

**Keywords:** Cancer, Drug discovery

## Abstract

Although cancer chemopreventive agents have been confirmed to effectively protect high-risk populations from cancer invasion or recurrence, only over ten drugs have been approved by the U.S. Food and Drug Administration. Therefore, screening potent cancer chemopreventive agents is crucial to reduce the constantly increasing incidence and mortality rate of cancer. Considering the lengthy prevention process, an ideal chemopreventive agent should be nontoxic, inexpensive, and oral. Natural compounds have become a natural treasure reservoir for cancer chemoprevention because of their superior ease of availability, cost-effectiveness, and safety. The benefits of natural compounds as chemopreventive agents in cancer prevention have been confirmed in various studies. In light of this, the present review is intended to fully delineate the entire scope of cancer chemoprevention, and primarily focuses on various aspects of cancer chemoprevention based on natural compounds, specifically focusing on the mechanism of action of natural compounds in cancer prevention, and discussing in detail how they exert cancer prevention effects by affecting classical signaling pathways, immune checkpoints, and gut microbiome. We also introduce novel cancer chemoprevention strategies and summarize the role of natural compounds in improving chemotherapy regimens. Furthermore, we describe strategies for discovering anticancer compounds with low abundance and high activity, revealing the broad prospects of natural compounds in drug discovery for cancer chemoprevention. Moreover, we associate cancer chemoprevention with precision medicine, and discuss the challenges encountered in cancer chemoprevention. Finally, we emphasize the transformative potential of natural compounds in advancing the field of cancer chemoprevention and their ability to introduce more effective and less toxic preventive options for oncology.

## Introduction

Cancer has become a major public health and economic issue in current society. According to GLOBOCAN 2022, there were 20 million new cases of cancer and 9.7 million deaths, and the number of new cancer cases was estimated to reach 35 million by 2050.^1^ The risk of developing cancer also tends to increase as the human development index elevated. Despite the rapid progress made in research on the etiology and molecular mechanisms of cancer, no fruitful results regarding improving overall cancer survival rates have been obtained. Nonetheless, in principle, cancer epidemiology have shown that all common cancers are largely preventable.^2,3^ Wherein, the concept of chemoprevention is increasingly popular, initially because it has successfully reduced the incidence rate of cardiovascular disease.^4,5^ Currently, the content of chemoprevention has expanded to include the use of natural, synthetic, and biological products to block, delay, or reverse carcinogenesis.^6^ Cancer chemoprevention has always been considered an important preventive strategy for reducing the burden of cancer on healthcare systems.^7–11^

**Fig. 1:**
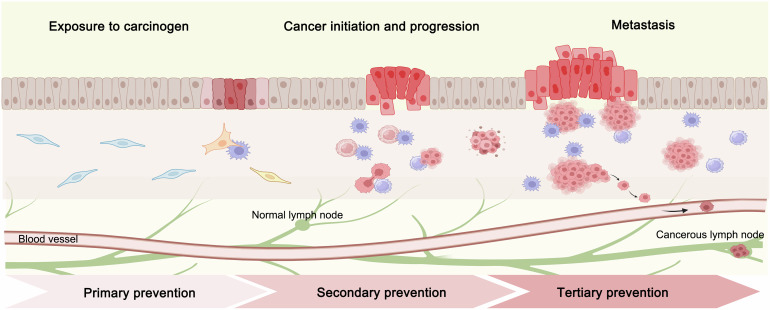
Cancer chemoprevention occurs through cancer initiation, progression, and metastasis. Cancer prevention can be effective throughout the entire process of cancer initiation and development. Primary chemoprevention can be achieved by altering diet, lifestyle habits, and increasing exercise to avoid or reduce exposure to known carcinogenic factors (chemical carcinogens such as formaldehyde, nitrosamine benzene, aflatoxin, etc.; physical carcinogenic factors, such as ultraviolet radiation, ionizing radiation, etc.; viruses such as human papillomavirus, hepatitis B virus, etc., and family genetic factors). Secondary chemoprevention helps suppress and reverse cancer. Tertiary chemoprevention aims to reduce the risk of tumor recurrence and metastasis, improve the quality of life and survival rate of patients. This figure was created with BioRender.com

Cancer chemoprevention refers to the use of natural, synthetic or biological products to prevent, slow down or reverse the development of cancer to reduce its incidence and mortality, and is usually divided into the following three levels (Fig. 1): (1) primary chemoprevention aims to prevent the development of diseases in the general population or especially high-risk populations; (2) secondary chemoprevention focuses on individuals diagnosed with a certain type of tumor or precancerous lesion that may develop into invasive cancer; (3) tertiary chemoprevention aims to inhibit cancer recurrence or secondary tumors.^7,12,13^ However, even after decades of development, only more than ten types of available drugs have been approved by the U.S. Food and Drug Administration (FDA) for cancer chemoprevention.^14,15^ Considering the constantly increasing incidence and mortality rates of cancer, it is of great significance to screen drugs for cancer chemoprevention from a large number of existing chemical substances. Moreover, given that the prevention process is lengthy, the chemoprevention process must always be safe, and the drugs used should be acceptable to both health and patients. Hence, the ideal chemopreventive agent should be nontoxic, inexpensive, and oral. Natural compounds have been a treasure reservoir for clinical use in cancer prevention and treatment for a long time.^16–18^ From 1981 to 2019, more than 60% of the small molecules approved for cancer treatment were natural products, natural product-derived, or natural product analogs.^16^ Natural compounds account for a significant proportion of currently used chemotherapy drugs, and a large number of studies have confirmed their chemopreventive efficacy.^9,17,19–22^ Due to the diverse chemical components, complex chemical structures, unique biological effects, high cost-effectiveness, ease of acquisition, low toxicity, and minimal side effects of natural compounds, there has been a greater focus on the use of natural compounds for cancer chemoprevention. In addition, the development of new preventive drugs with natural compounds as the lead compound has also emerged.^23,24^ Natural compounds have become a preferred approach in cancer chemoprevention.

In this review, we mainly discuss the concept, content, and classifications of cancer chemopreventive agents, highlight on exploring potential natural compound-derived cancer chemopreventive agents, and embark on a comprehensive investigation of multilevel regulatory mechanisms and therapeutic targets from the perspectives of targeted signaling pathways, immunotherapy, and gut microbiome regulation. We introduce various advances in drug delivery systems to assist in cancer chemoprevention. Clinical research progress of cancer chemoprevention in cancer treatment is also summarized and discussed. Moreover, we also summarize the various approaches that contribute to discovering anticancer natural compounds with low abundance and high activity to reveal the broad prospects of natural compounds as a treasure reservoir for cancer chemopreventive agent discovery. Meanwhile, we associate cancer chemoprevention with precision medicine to achieve individual precision cancer chemoprevention in the future. We also discuss the challenges encountered in cancer chemoprevention and expectations during the bench-to-bedside translation. Overall, we provide a comprehensive review of the field of cancer chemoprevention and emphasize the transformative potential of natural compounds in advancing the field of cancer chemoprevention and their ability to introduce more effective and less toxic preventive options for oncology.

## Agents for cancer chemoprevention

### FDA-approved medications for cancer chemoprevention

**Fig. 2:**
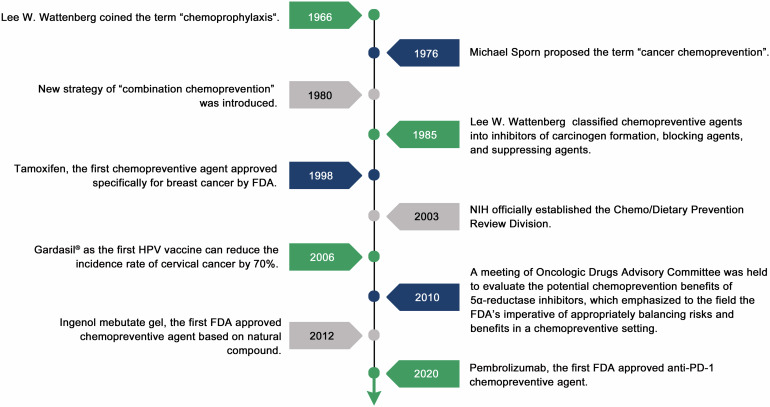
Historical timeline of important development in the field of cancer chemoprevention. FDA U.S. Food and Drug Administration, HPV human papillomavirus, NIH National Institutes of Health, PD-1 programmed cell death-1

Cancer chemoprevention is a rapidly developing field with broad prospects, but the long-term benefits of intervention may take many years or remain unknown. After the proposal of cancer chemoprevention, several agents were successfully approved by FDA for cancer prevention (Table 1). Many studies are also actively underway to expand the available agents for cancer prevention, and major development history of cancer chemoprevention are depicted in Fig. 2.

**Table 1:** Cancer chemoprevention agents approved by FDA

Cancer type	Agent and date first approved^a^	Property	Mechanism of action	Targeted cohort in indication	Reference
Breast cancer	Tamoxifen (1998)	Synthetic agent	Endocrine therapy	Reduce the incidence of breast cancer in (pre- and postmenopausal) women at high risk; reduce the risk of invasive breast cancer in women with ductal carcinoma in situ after breast surgery and radiation.	^25,783–786^
Breast cancer	Raloxifene (2007)	Synthetic agent	Endocrine therapy	Reduce the risk of invasive breast cancer in postmenopausal women at high risk for invasive breast cancer.	^787^
Skin cancer	Fluorouracil cream (1970)	Synthetic agent	Topical chemotherapy	Topical treatment of multiple actinic keratosis (AK).	^788^
Skin cancer	Photodynamic therapy (PDT) with 5-aminolevulinic acid (1999)	Synthetic agent	Combination therapy	Topical treatment of minimally to moderately thick AK of face or scalp.	^32,789^
Skin cancer	Diclofenac gel (2000)	Synthetic agent	Topical non-steroidal anti-inflammatory drug	Topical treatment of AK.	^790^
Skin cancer	Imiquimod cream (2004)	Synthetic agent	Immune modulator	Topical treatment of nonhyperkeratotic, nonhypertrophic AK of the face or scalp.	^791^
Skin cancer	Ingenol mebutate gel (2012)	Natural compound	Topical chemotherapy	Topical treatment of AK.	^33^
Skin cancer	Tirbanibulin (2020)	Synthetic agent	Microtubule inhibitor	Topical treatment of AK of face or scalp.	^792^
Bladder cancer	Bacillus Calmette-Guerin (BCG, 1990)	Biological product	Immunotherapy	Intravesical use for the prevention of carcinoma in situ of bladder and primary or recurrent stage Ta and/or T1 papillary tumors after transurethral resection.	^35,793^
Bladder cancer	Valrubicin (1998)	Synthetic agent	Chemotherapy	Intravesical therapy of BCG-refractory carcinoma in situ of the bladder, for whom immediate cystectomy would be associated with unacceptable morbidity or mortality.	^38^
Bladder cancer	Pembrolizumab (2020)	Biological product	Immunotherapy	Treatment of patients with high-risk non-muscle-invasive bladder cancer without in situ carcinoma and unresponsive to BCG, as well as who were declined or ineligible for radical cystectomy.	^794^
Esophageal cancer	PDT and porfimer sodium (2003)	Synthetic agent	Combination therapy	Ablation of high-grade dysplasia in Barrett esophagus patients.	^41^
Cervical cancer and other cancers associated with human papillomavirus vaccine (HPV) infection	HPV vaccine (Gardasil^®^, 2006)	Biological product	Quadrivalent vaccine for HPV types of 6, 11, 16, and 18.	Indicated in girls and women aged 9-26 for the prevention of the following diseases caused by HPV types included in the vaccine: cervical, vulvar, vaginal, and anal cancers caused by HPV types of 16 and 18, and the following precancerous or dysplastic lesions caused by HPV types of 6, 11, 16, and 18: cervical intraepithelial neoplasia (CIN) grades 1–3 and cervical adenocarcinoma in situ (AIS), vulvar intraepithelial neoplasia (VIN) grades 2 and 3, vaginal intraepithelial neoplasia (VaIN) grades 2 and 3, and anal intraepithelial neoplasia (AIN) grades 1–3. Indicated in boys and men aged 9-26 for the prevention of the following diseases caused by HPV types included in the vaccine: anal cancer caused by HPV types of 16 and 18, and the following precancerous or dysplastic lesions caused by HPV types of 6, 11, 16, and 18, and AIN grades 1–3.	^15,32,795^
Cervical cancer and other cancers associated with HPV infection	HPV vaccine (Cervarix^®^, 2009)	Biological product	Bivalent vaccine for HPV types of 16 and 18.	Prevention of cervical cancer, CIN grade≥2 and adenocarcinoma in situ; and CIN grade 1, caused by HPV types of 16 and 18 in girls and women aged 9-25.	^15,32,795^
Cervical cancer and other cancers associated with HPV infection	HPV vaccine (Gardasil^®^ 9, 2014)	Biological product	Nine-valent vaccine for HPV types of 6, 11, 16, 18, 31, 33, 45, 52, and 58.	Indicated in girls and women aged 9-45 for prevention of the following diseases: cervical, vulvar, vaginal, anal, oropharyngeal, and head and neck cancers caused by HPV types of 16, 18, 31, 33, 45, 52, and 58; and the following precancerous or dysplastic lesions caused by HPV types of 6, 11, 16, 18, 31, 33, 45, 52, and 58, CIN grade 2 and 3, and cervical AIS, CIN grade 1, VIN grade 2 and 3, VaIN grade 2 and 3, and AIN grades 1–3. Indicated in boys and men aged 9-45 for the prevention of the following diseases: anal, oropharyngeal and other head and neck cancers caused by HPV types of 16, 18, 31, 33, 45, 52, and 58; and the following precancerous or dysplastic lesions caused by HPV types of 6, 11, 16, 18, 31, 33, 45, 52, and 58, AIN grades 1–3.	^15,32,795,796^
Hepatocellular carcinoma (HCC)	Hepatitis B vaccine (HBV, Recombivax-HB^®^, 1986)	Biological product	HBV vaccine	Indicated for prevention of infection caused by all known subtypes of HBV (from birth onward).	^797^
HCC	HBV (Engerix-B^®^, 1989)	Biological product	HBV vaccine	Indicated for immunization against infection caused by all known subtypes of HBV (from birth onward).	^798^
HCC	HBV (Heplisav-B^®^, 2017)	Biological product	HBV vaccine	Indicated for prevention of infection caused by all known subtypes of HBV (for adults aged 18 years or older).	^799^
HCC	HBV (PreHevbrio^®^, 2021)^b^	Biological product	HBV vaccine	Indicated for prevention of infection caused by all known subtypes of HBV (for adults aged 18 years or older).	^800^

*AIN* anal intraepithelial neoplasia, *AIS* adenocarcinoma in situ, *AK* actinic keratosis, *BCG* Bacillus Calmette-Guerin, *CIN* cervical intraepithelial neoplasia, *HBV* hepatitis B virus, *HCC* hepatocellular carcinoma, *HPV* human papillomavirus, *PDT* photodynamic therapy, *VaIN* vaginal intraepithelial neoplasia, *VIN* vulvar intraepithelial neoplasia

^a^First approval for precancerous lesion or cancer prevention indication;

^b^PreHevbrio® has been voluntarily withdrawn from the market by the company, VBI Vaccines Inc., due to the bankruptcy of the company and the termination of its operations in November 2024.

Ta, noninvasive papillary carcinoma; T1, the tumor has spread to the connective tissue (known as the lamina propria layer), which separates the inner wall of the bladder from the underlying muscles, but the tumor has not invaded the bladder wall muscle layer

#### Chemopreventive agents for breast cancer

Intimate relationship exists between breast cancer and estrogen, and anti-hormone drugs are the first potential chemopreventive drugs to be tested in large-scale clinical trials. Among them, tamoxifen, a selective estrogen receptor (ER) modulator (SERM), is the first specifically cancer chemopreventive drug approved by FDA, which can reduce the risk of invasive and noninvasive breast cancer. At a median follow-up of 55 months, tamoxifen reduced the risk of invasive breast cancer by 49% and noninvasive cancer by 50%, as well as a reduction in hip, radius, and spine fractures was observed, while, the risk of endometrial cancer and the incidence of thromboembolic events also increase accordingly.^25^ In a meta-analysis, tamoxifen reduced the risk of ER-positive breast cancer by 48%.^26^ In study of comparing tamoxifen with placebo, the incidence of adverse reactions decreased and it was equivalent to that of patients taking placebo by 10 years (or 5 years after discontinuation of tamoxifen).^27^ In the latest study of tamoxifen and raloxifene, raloxifene has been proven to be less effective in reducing invasive breast cancer than tamoxifen, but it maintains a higher safety.^28^ At present, raloxifene is also approved to reduce the risk of invasive breast cancer in postmenopausal women with osteoporosis and in postmenopausal women at high risk.^29^ In addition, aromatase inhibitors such as ezetimibe and anastrozole have shown efficacy as chemopreventive agents, although they have not yet been approved.^30,31^

#### Chemopreventive agents for skin cancer

Actinic keratosis (AK) is an abnormal proliferation of epidermal keratinocytes caused by long-term ultraviolet irradiation, and about 10% of lesions can develop into invasive squamous cell carcinoma. Compared with other chemoprevention targets, skin is easier accessibility, which can carried out the direct local treatment.^7^ Currently, fluorouracil cream, diclofenac sodium gel, imiquimod cream, ingenol mebutuate gel, and tirbanibulin have been approved by FDA for the treatment of AK.^15,32^ Among them, ingenol mebutuate gel was a plant extract from *Euphorbia peplus* L., which provided an exciting natural compound-based chemopreventive option for skin cancer. Ingenol mebutuate gel is very effective for treating AK on face or scalp and body, exhibiting good tolerability, no systemic absorption, and excellent patient compliance in clinical settings.^33,34^ The successful application of chemoprevention in skin cancer provides great confidence for the development of chemopreventive agents.

#### Chemopreventive agents for bladder cancer and esophageal cancer

Bacillus Calmette-Guerin (BCG) is a live attenuated strain, initially used to treat tuberculosis, and it was proved that intravesical administration of BCG can treat carcinoma in situ of the bladder and reduce its recurrence.^35–37^ Besides, valrubicin and pembrolizumab were approved for the treatment of BCG-unresponsive carcinoma in situ of the bladder, with 21% and 41% patients achieved a complete response (CR), respectively.^38–40^ The approval of both BCG and pembrolizumab for carcinoma in situ of the bladder manifested that immunotherapy can play a role in cancer chemoprevention.

Photodynamic therapy (PDT) in combination with the photosensitizer drug porfimer sodium (PDT/PS) is licensed for the treatment of barrett esophagus with high-grade dysplasia. It was reported PDT/PS exhibited a superior complete ablation of Barrett esophagus compared with omeprazole (77% vs. 39%), as well as fewer patients progressed to cancer (13% vs. 28%).^41^ This therapy indicated that treating individuals with identifiable high-risk pre-invasive lesions can elaborate the cancer chemopreventive role.

#### Chemopreventive agents for cervical carcinoma and hepatocellular carcinoma (HCC)

New cases of gastric cancer, liver cancer, and cervical cancer worldwide reached 2.5 million in 2022, equivalent to one-eighth of all newly diagnosed cancer patients.^1^ The main carcinogenic factors (e.g., *Helicobacter pylori* (*H. pylori)*, human papillomavirus (HPV), and hepatitis B virus (HBV)) that cause the above-mentioned cancers are preventable. HPV infection has been widely recognized as a necessary cause of cervical cancer.^42,43^ The specificity of this virus is highly desirable as vaccination provides a highly effective chemoprevention method.^7^ The currently available HPV vaccines can protect against infection with HPV 16 and HPV 18, which account for 71% of cervical cancer cases.^44,45^ These vaccines are considered to be molecularly targeted because they generate immune responses against specific proteins.^46^ It was reported after 5–8 years of vaccination, the incidence of HPV 16 and 18 in girls aged 13–19 significantly decreased by 83%, and the incidence in women aged 20–24 significantly decreased by 66%.^47^ Global vaccination coverage with at least one dose in girls by 15 years of age was estimated to be 20% as of 2023.^48^ Moreover, the World Health Organization recommends getting the HPV vaccine before sexual activity to maximize its preventive effect.^49^ Chronic hepatitis B and hepatitis C are the most important causes of HCC and account for 21–55% of HCC cases globally.^50,51^ Statistical data from multiple countries and regions indicate that the popularity of HBV vaccination has significantly reduced the incidence rate of HCC.^52–54^

### Synthetic chemopreventive agents with clinical efficacy but lacking approval

Finasteride, a type of 5α-reductase inhibitor, has been shown can significantly reduce the prevalence of prostate cancer, but this protective effect is limited to lower-grade.^55,56^ Although finasteride has not been approved for the prevention of prostate cancer, multiple subsequent studies have supported its ability as prostate cancer chemoprevention.^57–59^ For example, finasteride can significantly decrease the risk of high-grade prostatic intraepithelial neoplasia.^60^ In the long-term follow-up study (median follow-up time of 18.4 years) of finasteride published by Goodman et al., finasteride reduced the risk of prostate cancer death by 25%, but it was not statistically significant due to the small number of prostate cancer deaths.^61^

Aspirin, a non-steroidal anti-inflammatory drug (NSAID) as well as cyclooxygenase (COX) inhibitor, can prevent the incidence rate and recurrence of colorectal cancer (CRC).^62,63^ Compared to a control group without aspirin, daily administration of aspirin halved the risk of colorectal metastatic adenocarcinoma and reduced distant metastases by 30–40%.^64^ In addition, it was advised that individuals with an average risk of CRC should be considered to employ low-dose aspirin for CRC prevention if they are not older than 70 years with a life expectancy of at least 10 years, not at high risk for bleeding, and have a 10-year cardiovascular disease risk of at least 10%.^65^ However, due to the significant gastrointestinal burden associated with long-term use of aspirin, its effectiveness as a chemopreventive agent is also limited.^66^

The selective COX-2 inhibitor celecoxib can significantly reduce the burden of colon polyps,^67^ and decrease the incidence of adenomas,^68^ and is more effective in reducing the risk of gastrointestinal bleeding, which has been approved to reduce the number of polyps in patients with familial adenomatous polyposis (FAP). Although clinically effective, it is associated with an increased risk of cardiovascular disease, leading manufacturer withdraw the CRC prevention function from its label, highlighting the necessity of long-term safety monitoring after drug approval.^15^

Sulindac, a NSAID drug, was reported standard doses of sulindac did not prevent the development of adenomas in subjects with FAP,^69^ however, the combination of difluoromethylornithine (DFMO) and sulindac can prevent recurrence of colorectal adenomas in patients at high risk by 70%.^70^ The most notable aspect of this study is the impact of DFMO and sulindac on the number and severity of new adenomas with only one case of advanced adenoma was detected in the combination therapy group, while 11 cases were observed in the placebo group,^70^ which provides impetus for further development of multi-drug combination therapy for cancer chemoprevention. In a meta-analysis of over 20,000 patients, the combination of DFMO and sulindac [relative risk, 0.24; 95% confidence interval (CI), 0.10–0.55] showed excellent protective effects compared to aspirin (relative risk, 0.77; 95% CI, 0.60–1.00), celecoxib (relative risk, 0.56; 95% CI, 0.31–1.01), and metformin (relative risk, 0.56; 95% CI, 0.22–1.39).^71^ Although the combination of DFMO and sulindac has a significant preventive effect, relatively cardiovascular adverse reactions are pronounced.

Diabetes is associated with an increased risk of several types of cancers.^72^ Metformin is an effective drug for treating diabetes, and because of its low cost, it is also considered as a promising cancer prevention strategy. A meta-analysis showed metformin reduced overall cancer incidence rate by 31% (summary relative risk, 0.69; 95% CI, 0.52–0.90), despite significant heterogeneity among studies (*I*^2^ = 88%).^73^ Besides, Higurashi’s study provided encouraging evidence for the cancer chemopreventive effect of metformin.^74^ One hundred and fifty-one patients who underwent resection of single or multiple colorectal adenomatous polyps were enrolled, and compared with placebo after just 1 year, a low dose of metformin (250 mg/day) significantly reduced the risk of total polyps (risk ratio, 0.67; 95% CI, 0.47–0.97) and adenomas (risk ratio, 0.60; 95% CI, 0.39–0.92). This study suggested the potential role of metformin in cancer prevention, however, large-scale and long-term trials still need to provide clear conclusions.

Statins are potent competitive inhibitors of 3-hydroxy-3-methylglutaryl-CoA reductase, commonly used as lipid-lowering drugs. In recent years, an increasing number of studies have shown that statins may have potential roles in the field of cancer chemoprevention. It was reported statins have an overall positive impact on the clinical outcomes of a series of cancers, including but not limited to CRC, HCC, gastric cancer, breast cancer, lung cancer, and kidney cancer.^75^ In a meta-analysis of over one million cancer patients, the use of statins was significantly associated with decreased risk of all-cause mortality [hazard ratio (HR), 0.70; 95% CI, 0.66–0.74] and cancer-specific mortality (HR, 0.60; 95% CI, 0.47–0.77) compared with non-users.^76^ Other meta-analysis also found that the use of statins is significantly associated with a reduced risk of HCC development,^77–79^ and this effect is dose-dependent, particularly evident in lipophilic statins.^78^ An observation study displayed regular use of aspirin and statins can reduce the risk of cancer with systemic inflammatory diseases.^80^ However, there is still controversy over the role of statins in cancer prevention, and more researches are needed to clarify their preventive effects.

There are also controversial chemopreventive agents, such as vitamin E and selenium, the results from a large-scale clinical study on the selenium and vitamin E cancer prevention trial with a follow-up period of at least 7 years exhibited the controversy surrounding the use of vitamin E and selenium as prostate cancer prevention agents.^81^ In another randomized clinical trial of selenium and vitamin E in patients with newly diagnosed non-muscle-invasive bladder cancer, neither selenium nor vitamin E supplementation affect disease recurrence or overall survival (OS) rate.^82^ The above researches on cancer chemoprevention agents provided valuable information for the field of cancer chemoprevention and have guiding significance for future experimental design. With the increasing cost of developing chemotherapy drugs, limited benefits, and the high economic burden, thus, as a solution, cancer chemoprevention requires the development of better and safer preventive agents.

### Potential biological products as chemopreventive agents

#### Vaccine

In addition to traditional cancer prevention vaccines such as HPV and HBV, of which exert cancer prevention effects based on known tumor-associated antigens, currently, multiple studies have pioneered new cancer vaccines with personalized and immune system activation characteristics, expanding the potential treasure trove of cancer chemopreventive agents.

CIMAvax-EGF consists of a chemical conjugate between epidermal growth factor (EGF) and P64, targeting the immune system and inducing anti-EGF antibodies, thus leads to a decrease in circulating EGF, thereby exerting its anticancer activity.^83^ It is worth noting that CIMAvax-EGF can also benefit patients who have not undergone genetic testing for mutations. Data shows that after treatment, the median OS of non-small cell lung cancer (NSCLC) patients is 22.46 months, with the survival rates at 6, 12, and 24 months were 97.7%, 82.7% and 45.5%, respectively. The median progression free survival (PFS) was 8.16 months, and the PFS rate at 6, 12, and 24 months were 55.4%, 36.4%, and 19.1%, respectively.^84^

Autogene cevumeran (BNT122), an individualized mRNA neoantigen vaccine containing up to 20 major histocompatibility complex class I (MHC I) and MHCII restricted neoantigens in lipoplex nanoparticles intravenously delivered, was reported can delay recurrence and prolong the survival time of pancreatic ductal adenocarcinoma patients.^85^ Sixteen patients were treated with atezolizumab and autogene cevumeran, then 15 patients with mFOLFIRINOX (comprising folinic acid, fuorouracil, irinotecan and oxaliplatin), the autogene cevumeran was tolerable and induced de novo high-magnitude neoantigen-specifc T cells in 8 out of 16 patients. After 18-month median follow-up, patients with vaccine-expanded T cells had a significant longer median recurrence-free survival compared with that without vaccine-expanded T cells (13.4 months, *P* = 0.003). Besides, the serum CA19-9 level of one patient increased and appeared a new liver lesion, while, a biopsy sample did not reveal malignant cells, indicated that the BNT122 vaccine may have the ability to eradicate micrometastases. Autogene cevumeran substantially expanded T cells that included vaccine neoantigen-specific, functional and durable CD8^+^ T cells, which can persist up to 2 years despite post-vaccination mFOLFIRINOX treatment, and the persistence of these T cells is associated with a longer median recurrence-free survival in vaccinated individuals. This is a phase I study with a small sample size, and subsequent studies has also conducted randomized phase II clinical trials on melanoma, NSCLC, CRC, etc (NCT03289962, NCT03815058, and NCT06534983).

mRNA-4157 (V940) is an mRNA-based personalized neoantigen therapy by encoding up to 34 tumor-specific antigens in a lipid nanoparticle formulation and tailored specifically for cancer patients, subsequently stimulating the immune system to produce reactive T cells targeting patient-specific tumor neoantigens, thereby achieving the goal of controlling and treating tumors. The most famous mRNA-4157 studies are KEYNOTE-603 and KEYNOTE-942. KEYNOTE-603 study mainly assessed safety, tolerability, and immunogenicity of mRNA-4157 in patients with resected NSCLC or resected cutaneous melanoma.^86^ This open-label, multicenter of phase I study taking mRNA-4157 as adjuvant monotherapy or combination therapy with pembrolizumab is safe and overall tolerable, and mRNA-4157 exhibited generation of de novo and enhancement of existing neoantigen-specific T-cell responses and provided mechanistic proof to support further development of mRNA-4157 for patients with resected solid tumors.^86^ Moreover, these T-cell responses are persistent and can even be detected 100 days after the last administration, indicated mRNA-4157 not only triggers strong immune responses, but these responses can be sustained for a long time. The latest research on mRNA-4157 (KEYNOTE-942) showed that combination of mRNA-4157 and pembrolizumab prolonged recurrence-free survival than monotherapy (HR, 0.561; 95% CI, 0.309–1.017), with lower recurrence or death event rate (22% vs. 40%), 18-month recurrence-free survival was 79% versus 62%.^87^ A phase III trial (NCT05933577) is conducting to confirm the safety and prevention cancer from returning in people with high-risk melanoma.

GNOS-PV02 can encode up to 40 neoantigens by using DNA plasmids as vectors, these neoantigens were determined through DNA, RNA, and germ cell DNA sequencing of tumor samples from each patient. After vaccination, it can stimulate the immune system to generate tumor-infiltrating lymphocytes to kill tumor cells. Yarchoan et al. used GNOS-PV02, DNA plasmid-encoded cytokine interleukin-12 (IL-12, as an adjuvant to enhance the response to neoantigens), and pembrolizumab in combination for the treatment of advanced HCC patients.^88^ The objective response rate (ORR) was 30.6% (11/36), with 8.3% (3/36) of patients achieving a CR, and the disease control rate was 55.6% (20/36). Compared to the historical value of ORR of pembrolizumab monotherapy of 18.3%,^89^ the efficacy of GNOS-PV02 combination therapy is about twice that of immunotherapy alone. It is worth noting that the target lesions of two patients continued to reduction of 44% and 59%, respectively. For most HCC patients, the high-capacity plasmid used by GNOS-PV02 is sufficient to cover neoantigens in the tumor, allowing the immune system to select the most effective neoantigens on its own.

Tumor vaccine can accurately target and block key stages of cancer occurrence, prolong survival time, delay cancer metastasis or recurrence. Compared to traditional synthetic prevention agents, its side effects are mild, usually including pain, redness, and swelling at the injection site. However, due to significant individual differences, many factors can affect the immune response of vaccines, thereby reducing their effectiveness in preventing cancer, and the immune evasion mechanism of tumor cells can weaken the preventive effect. Introducing novel natural drug carriers to prepare cancer immunotherapy-targeted vaccines might improve the aforementioned shortcomings.^90^ The neoantigens cancer vaccine is tailored specifically for each individual and is unique to each patient, with the continuous emergence of clinical evidence, the feasibility, safety, and promising clinical efficacy of cancer immunotherapy based on neoantigens have been demonstrated in cancer patients. With continuous exploration and research, the neoantigens cancer vaccine is expected to become a powerful solution for cancer prevention.

#### Immune modulator

The immune system is dedicated to protect organisms from harmful substances, and IL-2, as an important immune modulator, plays a crucial role in T-cell development and expansion, and has been approved by the FDA as an antitumor drug for patients with renal cell carcinoma (RCC) and melanoma.^91^ It was reported high dose (HD) IL-2 treatment yielded durable responses in metastatic RCC patients and prolonged survival.^92^ Among 356 patients receiving HD IL-2 treatment alone, 119 (33%) met the favorable risk criteria, 203 (57%) met the intermediate risk criteria, and 34 (10%) met the adverse risk criteria. The favorable and intermediate risk patients in this group demonstrated prolonged OS, and many experience years of treatment-free survival.^92^ The median OS for favorable, intermediate and poor risk groups treated with IL-2 alone is 64.5 months, 57.6 months, and 14 months, respectively, and the 2-year OS for those treated with IL-2 alone by risk category is 73.8, 63.7, and 39.8%, respectively.^92^ In radically operable CRC patients, pre-operative IL-2 immunoprophylaxis exhibited an encouraging effect.^93^ After a median follow-up of 72 months, 6/20 recurrences (30%) were observed in IL-2 pre-operative group, with 19/40 recurrences (47.5%) in controls. Furthermore, after a 5-year follow-up, 4/20 (20%) IL-2 treated patients were dead and the control patients were 19/40 (47.5%, *P* = 0.05).^93^ Interferon-α (IFN-α) is another cytokine approved by the FDA for the treatment of hematological malignancies and melanoma, it has been demonstrated to exert antitumor effect mediated by activation of immune system and antiangiogenic effect.^94^ However, due to the low response rate and high toxicity associated with HD IL-2 and IFN-α, the clinical application of these cytokines has been replaced by targeted therapy.

Cancer cells typically express tumor-associated antigen (TAA) that is different from normal cells, such as human epidermal growth factor receptor-2 (HER-2). Monoclonal antibodies (mAbs) can specifically recognize and bind with these TAAs, thereby blocking the growth, proliferation, invasion, and metastasis of cancer cells. Trastuzumab, a classical anti-HER-2 mAb, inhibits the growth of HER-2 positive breast cancer cells through specific binding with HER-2 receptors,^95^ and plays a leading role in adjuvant therapy and metastatic therapy of breast cancer. Researches show that trastuzumab adjuvant chemotherapy significantly improves the disease-free survival rate of HER-2 positive breast cancer patients, and reduces the risk of recurrence and death.^96–98^ Although other anti-HER-2 drugs are currently available, such as pertuzumab and lapatinib, trastuzumab remains the gold standard for treating HER-2-positive breast cancer.^99^ If TAA with abnormal expression in the body can be identified before cancer occurs and mAbs can be used for intervention in a timely manner, it may prevent the formation or development of cancer cells.

Biological products with potential cancer chemopreventive effects have highly specific and personalized preventive potential, and most of them with the advantages of strong immune regulatory effects, which may prevent cancer from occurring at the source. However, due to tumor heterogeneity and individual genetic differences, it may affect its preventive effect. In addition, as most biological products work by regulating the immune system, which might cause immune-related adverse reactions (irAEs), and long-term use may lead to changes in immune system tolerance, affecting normal immune function. In addition, biological products usually require special storage and transportation conditions, and injection is required for use. The economic burden brought by expensive prices should not be underestimated, too. Therefore, the widespread use of cancer chemopreventive agents based on biological products is limited. Considering the prevention process is lengthy, the agents used should be nontoxic, inexpensive, and take orally to both health and patients.

### Natural compounds as promising cancer chemoprevention agents

#### Natural compounds for cancer chemoprevention at the preclinical stage

**Fig. 3:**
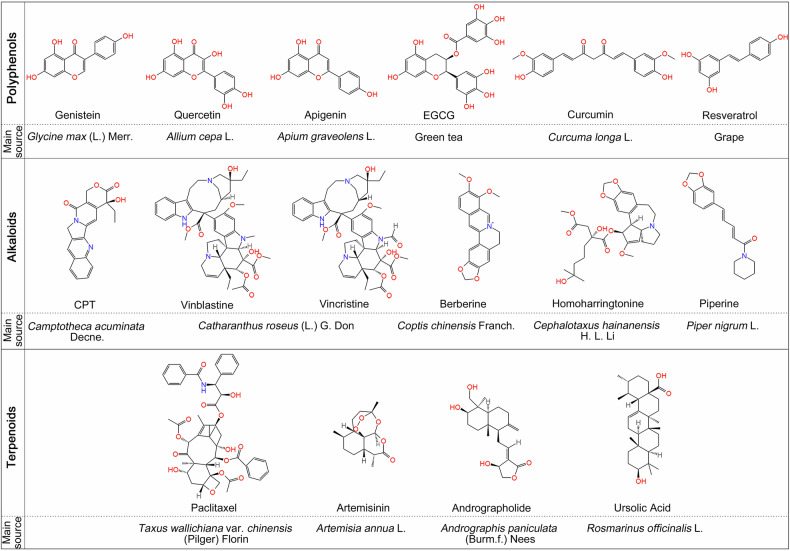
The chemical structures and main source of presentative natural compounds exhibiting cancer chemoprevention. EGCG epigallocatechin-3-gallate, CPT camptothecin

Unlike many synthetic drugs, the consumption of natural compounds extracted from food, plants, herbs, etc. has been considered as relatively safe for thousands of years, which has brought new vitality to the field. In the past few decades, natural compounds have gradually become the main source of new candidate drugs. Due to the effective tumor targeting potential and low toxicity to normal tissues, natural compounds have become the potential cancer chemoprevention drug for use alone or in combination with other chemotherapy drugs.^100–102^ In this section, we introduced the classical and widely studied natural compounds with cancer chemoprevention characteristics, and demonstrated their potentiality in cancer chemoprevention (Fig. 3).

##### Polyphenols

Polyphenols are a large class of compounds with over 8000 structural variants.^103^ Most polyphenols have pharmacological activities, such as anti-inflammatory, immunomodulatory, and anticancer effects.^104,105^

*Genistein:* Genistein is a multifunctional natural flavonoid mainly derived from soybeans and their products, and it is a typical example of a phytoestrogenic compound. Researches have shown a relationship between a high soy products and cancer prevention. Soy food consumption was associated with a reduced risk of prostate cancer in men,^106,107^ and it was negatively associated with the risk of breast cancer in Asian women. While, this association has not been confirmed in Western women.^108,109^ Moreover, soybean isoflavone intake can reduce the risk of breast cancer in both premenopausal and postmenopausal women in Asian countries.^110^ The mechanism for their cancer chemoprevention involves inducing apoptosis, cell cycle arrest, anti-proliferation, reducing angiogenesis, acting on cancer stem cells (CSCs), regulating gene and mRNA expression, and its unique estrogenic properties. Wherein, genistein induces cell apoptosis through multiple pathways, such as stimulation of the peroxisome proliferator-activated receptor gamma,^111^ inactivation of the insulin-like growth factor (IGF)-1 receptor/p-Akt signaling pathway,^112^ activation Ca^2+^-dependent pro-apoptotic proteases,^113^ inhibition the accumulation of lipid droplets,^114^ regulation endoplasmic reticulum stress, mitochondria-dependent or reactive oxygen species (ROS)-dependent pathways,^115,116^ etc. Through inactivating DNA repair pathways, such as homologous recombination and nonhomologous end joining pathway,^117,118^ regulating epigenetic proteins,^119–121^ modulating microRNAs (miRNAs),^122–126^ genistein thus exhibited the cancer chemoprevention role. In addition, due to its unique estrogenic properties, genistein can disrupt estrogen binding within molecules and affect estrogen-dependent pathways in a dose-dependent manner,^127,128^ It can effectively inhibit the development of breast cancer by blocking estrogen and inhibiting tumor cell proliferation, because it has a strong affinity for ERβ than ERα.^129^ The above preclinical studies collectively support the role of genistein in preventing, retarding, and blocking cancer development, indicating genistein is a promising chemopreventive drug.

*Quercetin:* Quercetin has a wide range of sources and potential health benefits, which mainly from *Allium cepa* L., *Crataegus pinnatifida* Bge., apple, etc. It has been added as a commercial dietary supplement to functional foods, as well as playing a role in the prevention and treatment of cancer.^130^ A great deal of researches have been proved the effect of quercetin in preventing the growth, proliferation and progression of cancer,^131–135^ In addition, quercetin as an antioxidant, can protect cells from oxidative stress and reduce DNA damage by regulating ROS signaling pathways, and maintain redox homeostasis.^136,137^ Meanwhile, quercetin can also impact and specifically target oncogenic miRNA^138^^,^^139^ and long non-coding RNA (lncRNA).^140–142^ According to reports, it is safe to orally administration quercetin 1 g/day, and it can absorbed up to 60%.^143^ Quercetin is mainly absorbed by intestinal cells in the form of glycosides, hydrolyzed into aglycones, and enters the intestinal cavity.^144^ In addition, gut microbiota play important role in the production of glycosidases and enzymes, which can transfer quercetin to smaller, and more easily absorbed molecules.^145,146^ Furthermore, no toxicity or side effects of quercetin have been observed in the adults within a reasonable dosage range.^147^

*Apigenin:* Apigenin is a plant-derived flavonoid widely found in *Apium graveolens* L., *Coriandrum sativum* L., and *Matricaria recutita* L., with the health-promoting effect due to its low toxicity and significant effects on normal vs. cancer cells.^148,149^ Apigenin induces various programmed cell death processes such as apoptosis, autophagy, and stimulates immune responses, inhibits cell cycle progression, migration, and invasion, suppresses CSCs by modulating signaling pathways involved in tumor development and progression.^150,151^ In addition, the interplay between apigenin and miRNA,^152–154^ lncRNA^155^ is also a new avenue for cancer prevention. The chemopreventive potential of apigenin has been demonstrated, but further researches are needed to validate apigenin as a potential chemopreventive agent.

*Epigallocatechin-3-gallate (EGCG):* EGCG is the most important and abundant polyphenol in green tea, which exerts various biological effects by regulating signaling pathways, such as anti-DNA methylation, anti-apoptosis, anti-inflammatory, anti-angiogenesis, and anti-metastasis.^156–158^ EGCG is composed of three aromatic rings and connected by a pyran ring, which is believed to be the cause for its biological activity.^159,160^ Recent research progress supports the potential role of EGCG in chemoprevention of various cancers by interfering with cancer hallmarks, including sustained proliferation signaling, evasion of growth inhibitors, resistance to cell death, induction of angiogenesis, activation of invasion and metastasis, energy metabolism reprogramming and evasion.^158^ EGCG demonstrates a multifunctional mechanism of action by targeting these features to provide possibilities for cancer prevention. In clinical trials, EGCG concentrations usually range from 100 mg to 600 mg/day.^161^ It was reported patients with Down’s syndrome received EGCG (600–800 mg/day) for 12 months have good safety and tolerability, with no significant changes in aspartate transaminase, alanine transaminase, and electrocardiogram.^162^ As the newly reviewed safety of EGCG by European Food Safety Agency, a daily intake of 800 mg or more of EGCG as a food supplement can induce a significant increase in serum transaminases of the human body,^163^ which might be a guidance for the dosage for clinical studies.

*Curcumin*: Curcumin is an acidic polyphenolic compound mainly presented in *Zingiberaceae* plant of *Curcuma longa* L. Its molecular feature characterized by its unique polyphenolic framework, especially the two feruloyl groups connected by methylene, which are responsible for its anti-inflammatory and antiproliferative biological properties.^164,165^ Numerous studies have shown that curcumin can inhibit cancer cell proliferation, migration, and metastasis by regulating multiple signaling pathways to induce cancer cell apoptosis, inhibit tumor angiogenesis, reverse multi-drug resistance, improve epigenetics.^166–168^ Moreover, curcumin exhibited safe, effective, and well-tolerated characteristics, and is an encouraging drug in chemoprevention. Curcumin is recognized as generally safe by the FDA, and healthy individuals can tolerate up to 12,000 mg/day of curcumin without adverse reactions.^169^ In a study of curcumin in the treatment of patients with advanced pancreatic cancer, oral curcumin is tolerated without toxicity at doses of 8 g/day for up to 18 months.^170^ Moreover, it is worth noting that the safe dosage range of curcumin may vary greatly depending on the type of cancer being treated.^171^

*Resveratrol:* Resveratrol exists in various plants, such as grapes, blueberries, *Polygonum cuspidatum* Sieb. et Zucc., as well as plenty genera of fungi, including *Botryosphaeria*, *Penicillium*, *Cephalosporium*, etc.^172^ Resveratrol has been reported with plenty of health benefits, and there were compelling evidences demonstrated its anti-inflammatory, antioxidant, antiviral, anti-aging roles, as well as retarding the growth of a variety of cancer cells in vitro and in vivo models.^173–177^ It has shown that administrating 1.0 g of resveratrol can provide a 0.6 mM maximum plasma concentration, and it occurs within 1 h.^178–181^ Although the absorption rate of resveratrol in the systemic circulation is very low, its absorption rate can exceed 70%.^182,183^ It is worth noting that the blood concentrations of glucuronide and sulfate conjugates in resveratrol are higher than those of free resveratrol, confirming the rapid metabolism of resveratrol and indicating that it may undergo enteropathic recirculation. Gut microbiota have been shown can promote the synthesis of resveratrol from its precursor, thus improving its bioavailability.^184,185^ Therefore, regulating the composition of the microbiota composition is one of the mechanisms by which resveratrol exerts cancer chemoprevention.^174,186^

##### Alkaloids

Alkaloids, as natural bioactive compounds, play an important role in the development of anticancer drugs.^187–189^ It also constitutes a reserve for the lead compounds for drug discovery. Some of these alkaloids have been approved by the FDA as chemotherapy drugs, such as camptothecin (CPT),^190^ a well-known inhibitor of topoisomerase I, and vinblastine,^191^ which interacts with microtubule proteins to cause mitotic disasters.

*Camptothecin (CPT):* The discovery of the anticancer activity of CPT is considered as a great breakthrough, it was isolated from the bark and stem of *Camptotheca acuminata* Decne., it has been used in clinical treatment of certain cancers in the 1970s.^192^ CPT exerts anticancer effects by inhibiting topoisomerase I (Top 1)-DNA complexes, referred as “Top 1 covalent complex”, the main target of CPT, rather than free TOP 1 enzyme.^193,194^ Under normal physiological conditions, the equilibrium between unbound Top 1 and TOP 1 covalent complex shifts towards the free enzyme, while, under the effect of CPT, this balance strongly shifts to the formation of ternary complex, abating the amount of free Top 1 thus eventually diminishing its effect.^193^ Although CPT exhibits significantly higher selectivity towards the Top 1 to cancer cells, its cytotoxic effect can also affect normal healthy cells.^195^ Wherein, the most outstanding side effects include myelosuppression and gastrointestinal toxicity. In order to overcome the drawbacks of toxicity and poor stability of CPT, researchers have attempted to prepare it into novel drug delivery systems such as nanoformulations.^196^

*Vinblastine and vincristine:* Vinca alkaloids was the first type of alkaloid applied in cancer therapy, mainly originated from *Catharanthus roseus* (L.) G. Don, with vinblastine, vincristine, and vinorelbine of well-known, which are the second-most widely used chemotherapy compounds for the treatment of various cancers, especially in combination chemotherapy regimens.^197^ Among them, vinblastine and vincristine as natural compounds, have been approved for use in United States.^198^ Vinca alkaloids are effective anti-mitotic chemotherapy drugs through inhibiting cell proliferation by binding to microtubules, which can cause mitotic arrest and cell apoptosis.^199^ Vinca alkaloids have typical peripheral neurotoxicity, mainly manifested as peripheral, symmetric multiple sensor-motor and autonomic neuropathy.^200^ In addition to autonomic dysfunction, gastrointestinal toxicity can also be observed.

*Berberine:* Berberine is a natural isoquinoline alkaloid mainly distributed in *Coptis chinensis* Franch., *Phellodendron chinense* Schneid., etc. Convincing studies have shown berberine possesses diverse pharmacological activities of anti-inflammatory, antibacterial, anti-diabetic effects, as well as exhibits preventive and therapeutic effects on various types of cancer.^201–205^ For example, berberine plays a reliable chemopreventive role in the formation of CRC, which can alleviate intestinal ecological imbalance, increase the abundance of beneficial bacteria, regulate small-molecule metabolism of intestinal microorganisms and intestinal inflammation, thus control intestinal mucosal inflammation.^206–209^ In the stage of CRC, in addition to regulating the homeostasis of gut microbiota, berberine exerts the effect of inhibiting cell proliferation, invasion, and metastasis, blocking the cell cycle, inducing cell apoptosis, regulating cell metabolism, suppressing angiogenesis, and enhancing chemical sensitivity, similar to other active molecules.^210^

*Homoharringtonine:* Homoharringtonine can be extracted from *Cephalotaxus hainanensis* H. L. Li, known as a notable bioactive compound in hematological malignancies, it has been used in the clinical treatment of leukemia in China since 1970s, and it was approved by FDA for the treatment of chronic myeloid leukemia (CML) with resistant to tyrosine kinase inhibitors in 2012.^211,212^ Homoharringtonine mainly suppressing translation process by affecting the A site in the ribosome, thus effectively inhibiting the initial elongation step of protein synthesis, which is crucial for the survival and proliferation of cancer cells.^213,214^ Homoharringtonine also targets the phosphorylated serine 209 residues of the eukaryotic translation initiation factor eIF4E, leading to the degradation of phosphorylated proteins and hindering the growth of leukemia cells.^215^ Although it is currently used for the treatment of leukemia in clinical practice, studies have displayed its inhibitory effects on different types of cancer.^216–220^

*Piperine:* Piperine is a widely distributed dietary phytochemical compound, mainly founded in *Piperaceae* family, with a broad spectrum of pharmacological activities, including anti-inflammatory, antibacterial, and antitumor properties.^221^ Piperine as a cancer chemopreventive agent can affect cancer cells in various ways, such as influencing cell apoptosis signaling, inhibiting proliferation and survival, restraining invasion and metastasis, affecting redox homeostasis, regulating endoplasmic reticulum stress and autophagy, and blocking drug metabolism enzymes, etc.^222^ In addition, piperine can enhance the biological effects of many important therapeutic nutrients and drugs.^223–225^ Human consumption of piperine in black pepper (5 mg/kg/day) has been determined to have no adverse effect, and subacute toxicity test demonstrated a dose of 100 mg/kg pepper is nontoxic in mice.^226,227^ More interestingly, piperine does not undergo any metabolic changes during absorption, as it is found in both intestinal tissue and serous fluid.^226^

##### Terpenoids

*Paclitaxel:* Paclitaxel is naturally produced in the bark and needles of *Taxus wallichiana* var. *chinensis* (Pilger) Florin, is one of the most successful and widely used natural anticancer drugs, and listed on the World Health Organization Essential Medicines List.^228,229^ Due to the low yield and high cost of extracting paclitaxel from plants, most of the current paclitaxel is obtained from chemically synthesized or genetically modified endophytic fungi, with its trade name of Taxol.^229^ Paclitaxel is a mitotic inhibitor that targets microtubules and induces cell cycle arrest, and ultimately leads to cancer cell death.^230^ In addition, paclitaxel can also induce antitumor immunity on various types of immune cells, and the interaction with the immune system is crucial for single or combination therapy.^231^

*Artemisinin:* Artemisinin, a gift from traditional Chinese medicine to the world, was extracted from *Artemisia annua* L., and its discovery has led to the Nobel Prize in Physiology or Medicine to Youyou Tu in 2015.^232^ In addition to its well-known anti-malaria effect, it also has excellent antitumor properties. Its biological activity is mainly attributed to the internal endoperoxide trioxane moiety in its sesquiterpene lactone structure.^233,234^ The cytotoxic activity of artemisinin in multiple cell lines is attributed to free iron, as it is necessary to activate artemisinin.^235–237^ In the presence of free iron, artemisinin can convert itself into cytotoxic carbon center radicals, an efficient alkylating agent that induces direct oxidative damage to cancer cells.^235^ Moreover, artemisinin upregulates intracellular free ion levels, promotes the accumulation of lipid peroxides in cells, thus induce ferroptosis, and retard the development of cancer.^238–240^ In addition, artemisinin can induce cancer cell death by disrupting cell membranes, as well as induce cell apoptosis, inhibit angiogenesis, proliferation, and migration of endothelial cells, and improve sensitivity to chemotherapy or radiotherapy.^241,242^

*Andrographolide:* Andrographolide is the major bioactive ingredient present in *Andrographis paniculata* (Burm.f.) Nees, with immunosuppressive, antipyretic, analgesic, antitumor, antiviral and anti-inflammatory properties.^243,244^ Andrographolide showed promising antitumor effects in preclinical studies, established the foundation for its cancer chemoprevention and cancer treatment. It was discovered that andrographolide has the highest concentration distribution in the kidneys,^245^ and the maximum concentration was 58.62 ng/mL when oral administration of 200 mg of andrographolide after 1.6 h, with the elimination half-life of 10.5 h.^246^

*Ursolic acid:* Ursolic acid is the most predominant representative of pentacyclic triterpenoids, obtained from various plants and fruit of *Rosmarinus officinalis* L., *Salvia japonica* Thunb., apple peel, and possesses considerable pharmacological activities.^247–249^ In preclinical studies, ursolic acid is widely known as a cancer chemopreventive agent with the potential to manage the neoplastic progress and target caner hallmarks at various phases.^250^ Pharmacokinetics studies indicated that the plasma concentration of ursolic acid is low even though the oral administration of doses up to 300 mg/kg, and the removal half-life is relatively short.^251^ Research has revealed that ursolic acid has low cytotoxicity, and even with a daily intake of up to 9.26 g/kg can be well-tolerated.^252^ The limited bioavailability, solubility, and rapid metabolic characteristics of ursolic acid hinder its clinical application. Therefore, alternative strategies of constructing its analogs or novel delivery systems were able to improve its bioavailability.

According to a great deal of published results, it has been found that natural compounds with cancer prevention activity come from a wide range of sources. In general, the above-mentioned natural compounds have great potential as candidate cancer chemopreventive agents, and further in vivo and in vitro research is needed to explore their targets and mechanisms of action, to provide data support for the translation into clinical applications.

## Therapeutic targets of natural compounds as cancer chemopreventive agents

### Targeting classical signaling pathways

Testing the potential of cancer chemoprevention drugs mostly starts from in vitro research of cell lines, followed by in vivo tests, to measure the incidence rate, size and quantity of tumors. These in vitro*/*in vivo studies help to understand the molecular mechanism of the chemoprevention effect of natural compounds. Given that previous researches have comprehensively reviewed oxidative stress and antioxidant defense celluar signaling molecules as targets, such as nuclear factor erythroid 2-related factor 2 (Nrf-2)/antioxidant response element (ARE) pathway,^22,253,254^ activator protein 1,^17^ matrix metalloproteinases (MMP)^255^ of the role in cancer chemoprevetion, and we will not go into much detail here. We overviewed the preclinical research results of natural compounds in the field of cancer prevention in past decade, focusing on classic cancer-related signaling pathways, as well as their main targets and pathways, which mainly including mitogen-activated protein kinase (MAPK) signaling pathway, phosphoinositide 3-kinase (PI3K)/Akt/mammalian target of rapamycin (mTOR) signaling pathway, wingless (Wnt)/β-catenin signaling pathway, nuclear factor kappa-B (NF-κB) signaling pathway, Janus kinase (JAK)/signal transducer and activator of transcription (STAT) signaling pathway, Hippo signaling pathway, Hedgehog pathway, and signaling pathway-associated crosstalk.

#### MAPK signaling pathway

MAPK signaling pathway is a complex and interrelated signaling cascade that frequently involved in tumor development, progression, and drug resistance.^256^ The MAPK cascade is composed of signal transduction partially regulated by phosphorylation and is a highly conserved tertiary kinase model, mainly composed of MAPK kinase kinase (MAPKKK), MAPK kinase (MEK, also known as MAPKK), and MAPK.^257,258^ The relevant signals stimulate the upstream kinase MAPKK, and respond by activating the intermediate kinase MAPKK, followed by activating the downstream kinase MAPK.^259,260^ The three primary MAPKs are extracellular signal-regulated kinase (ERK), c-Jun N-terminal kinase (JNK), and p38 kinase.^261,262^ The most extensively studied MAPK pathway is the RAS/RAF/MEK/ERK signaling, which occupies a central position in controlling cell proliferation and differentiation, as well as control various aspects of cancer cell metabolism.^263,264^ JNK and p38 signaling also play essential roles in MAPK cascade, they can triggered by various damages mainly activated by MKK4/7 and MKK3/6 kinases, respectively.^265,266^ Natural compounds can also exert anticancer effects by regulating the MAPK pathway.

##### Polyphenols

The cytoskeleton adapter protein vinculin can regulate the interaction between focal adhesion kinase and paxilin through ERK pathway,^267^ and a high concentration of genistein with 100 μM can dramatically restrain the expression levels of p-FAK, p-paxillin, tensin-2, vinculin, and α-actinin, furthermore, p-p38, p-ERK, and p-JNK levels were also significantly lessened by genistein in B16F10 melanoma cells.^268^ In HeLa cells, genistein was also reported to markedly suppressed the phosphorylations of p38 and p42/44 proteins in the MAPK pathway to inhibit the migration and invasion.^269^ Osteopontin plays significant role in determining the metastatic potential of various malignant tumors,^270^ it can be inhibited by genistein in MDA-MB-435 and MDA-MB-231 cells, meanwhile, genistein can also decreased p-ERK1/2 and p-MEK1/2, and no alteration was observed in nucleus.^271^ Quercetin treatment could promote apoptosis, lead G1 cell cycle arrest and inhibit migration of PC-3 and CD44^+^/CD133^+^ cells via decreased the expression of p-ERK1/2 and p38 in MAPK pathway.^272^ Quercetin also shows antitumor effect on melanoma cells by increasing the expression of Bcl-2 associated X protein (Bax), p-JNK, p-p38, p-ERK1/2, and cleaved poly-ADP ribose polymerase and decreasing Bcl-2 in vitro, the further in vivo study also displayed that quercetin can significantly reduce tumor volume and increase the expression of p-JNK and p-p38.^273^ In Tca8113 cells, apigenin restrains the p-MEKK1, p-ERK1/2, thus prevent the proliferation, invasion, and migration of tumor cells.^274^ Li et al. elucidated apigenin can inhibit HepG2 cell growth via inducing G1 arrest and activating p38 MAPK pathway by elevating the levels of phosphorylation p38 and decreasing p21.^275^ In CML cells, the proliferation inhibition and apoptosis induction caused by EGCG was associated with the increased expression of p-JNK and the decreased p38.^276^ It was reported the levels of phosphorylated ERK and p38 MAPK can be substantially enhanced when the chemo-resistant A549 sublines were treated with curcumin. Knocking down p38 MAPK can significantly reduce curcumin-induced cell apoptosis, and it is suggested activated p38 MAPK signaling was considered as a pro-apoptotic signal for curcumin-induced apoptosis of chemo-resistant human lung cancer cells.^277^ The study of Zhu et al. provided evidence to confirm the effect of curcumin in activating JNK and p38 but suppressed ERK and p-p65, which leads to SHI-1 tumor of diminished volume.^278^ Curcumin can inhibit human placental choriocarcinoma cells via activating MAPK signaling by elevating the expression of p-ERK1/2, JUN, p-p90RSK, p–c-JUN.^279^ The aberrant expression of bone morphogenetic proteins (BMPs) was related to the pathogenesis of various types of cancer, and resveratrol can effectively restrain the proliferation and promote the apoptosis of LoVo cells, which was related with the upregulation of BMP9 to activate p38 MAPK.^280^ A growing number of evidence indicate that there is a close relationship between diabetes and pancreatic cancer, and resveratrol represses hyperglycemia-driven ROS-induced invasion and migration of pancreatic cancer cells through inhibiting of ERK and p38 MAPK signaling pathways.^281^ Resveratrol has also increased the relative expression of Beclin1 and LC3-II/I while decreased p62 expression, suggesting that resveratrol induced autophagy in NSCLC cells.^282^

##### Alkaloids

Piperine can activate caspase-3 and caspase-9, and cleaved PARP, as well as reduce the phosphorylation of JNK and p38 MAPK in A2780 cells, indicated piperine could induce cell death through the JNK/p38 MAPK-mediated intrinsic apoptotic pathway in ovarian cancer cells.^283^ IL-6 as a multifunctional inflammatory cytokine, various studies have shown that higher serum IL-6 concentrations are closely associated with advanced tumor stages and shorter survival periods.^284,285^ Piperine was reported can suppress TMK-1 cell invasion via lessening IL-6 expression through inhibition of p38 MAPK signaling.^286^ Berberine can also decrease the IL-8 expression in vitro and in vivo through the inhibition of the phosphorylation levels of p38 MAPK, ERK1/2 and JNK in gastric cancer MGC-803 cell.^287^ It was shown whether MG-63 cells were treated with berberine and cisplatin alone or combination, the MAPK signaling was inhibited manifested by the downregulation of expression of p-p38, p-JNK, and p-ERK.^288^ Zheng et al. validated berberine restrained the growth and induce cell cycle arrest in G0/G1 phase, and apoptosis in NSCLC cells through p38α MAPK-mediated induction of Forkhead box O3a and p53, followed by p21 protein expression.^289^ Homoharringtonine not only has a significant effect on the treatment of leukemia related diseases, but also inhibits the proliferation of LoVo cell growth, this effect is mainly achieved through inhibition EphB4 and the downstream MAPK/EKR1/2 signaling pathway, suggested homoharringtonine might be a promising EphB4 inhibitor for CRC treatment.^290^

##### Terpenoids

Andrographolide inhibits growth of human T-cell acute lymphoblastic leukemia Jurkat cells by upregulation of p-p38 expression, it can also constrict Jurkat xenografts tumor growth in vivo.^291^ In Huh-7 and SK-Hep-1 cells, the treatment of ursolic acid can affect the anti-apoptotic protein of Mcl-1, Bcl-xL, Bcl-2, TCTP, and apoptosis-related proteins tumor necrosis factor-α (TNF-α), Fas, FADD, Bax, cleaved caspase-3, caspase-8, caspase-9, PARP, as well as significantly upregulation the expression of p-ERK1/2 and p-JNK, and downregulation p-p38.^292,293^ In addition, ursolic acid can trigger caspase-dependant and ERK1/2 MAPK associated-apoptosis in osteosarcoma MG-63 cells.^294^ Artemisinin also displays the inhibitory effects on osteosarcoma. Artemisinin could induce the phosphorylation of cAMP response element-binding protein (CREB) via the activation of p38 MAPK signaling in osteosarcoma cells, and the phosphorylation of CREB can also bond specifically to the promoter of secretion of thrombospondin-1 (TSP-1) and promote its transcriptional activation, suggest the p38 MAPK/CREB/TSP-1 signaling cascade might be a potential therapeutic target for osteosarcoma.^295^

#### PI3K/Akt /mTOR signaling pathway

The PI3K/Akt/mTOR signaling pathway is a highly conserved and important transduction network in all higher eukaryotic cells, involved in the processes of cell survival, growth, and proliferation, etc.^296^ It’s one of the most frequently dysregulated pathways in human cancers,^297–299^ causing apoptosis deregulation and chemotherapeutic resistance,^300,301^ and also a common target of natural compounds regulation.^300,302–304^

##### Polyphenols

As the estrogen receptor like effects of genistein, it reduces cell cycle arrest and apoptosis with altered p-FAK, p-PI3K, p-Akt, p-GSK3β, p21 or cyclin D1 expression in ovarian cancer cells.^305^ Genistein can also play the chemopreventive potential by inducing G2/M arrest and apoptosis in T24 cells through ROS-dependent blocking of the PI3K/Akt signaling pathway.^306^ After quercetin treatment, ERα and PI3K/Akt/mTOR signaling were downregulated, concurrently with the inhibition of CD44^+^/CD24^−^ viability and clone formation, implied quercetin targeted cancer stem cells to promote tumor eradication.^307^ Quercetin might be a good candidate drug for the treatment of invasive B-cell lymphoma, as it reduced the release of IL-6 and IL-10, inhibited the PI3K/Akt/mTOR and STAT3 pathways and induce primary effusion lymphoma cells death. Quercetin also decreases the expression of latent and lytic Kaposis’ Sarcoma-associated Herpesvirus proteins, increases the human leukocyte antigen DR and calreticulin to make the dying cells more easily detected by the immune system.^308^ Apigenin can also inhibit HCC cell proliferation and induce autophagy by inhibiting the PI3K/Akt/mTOR pathway, and 3-methyladenine and Atg5 genes silencing enhanced apigenin-induced proliferation inhibition and apoptosis, evidenced that the combination of autophagy inhibitors and apigenin would be a potential chemotherapy strategy for HCC treatment.^309^ It was reported IGF2 was the target of curcumin, by suppressing IGF2, PI3K-p85, Akt, mTOR, eIF4E-bind-ing protein 1 and ribosomal protein S6 kinase beta-1 expression in bladder cancer cell to exhibit the mechanism of suppressing IGF2-mediated PI3K/Akt/mTOR signaling pathway.^310^ By inhibiting the PI3K/Akt/mTOR pathway, curcumin can also exert cytotoxic effect on A549 cells with significantly increasing the expression of Beclin1 and LC3-II, and reducing p62 levels to induce autophagy.^311^ In the mechanisms study of curcumin in laryngeal squamous cell carcinoma (LSCC), it was found miR-145 was significantly downregulated in LSCC cells and tissues, and curcumin can dramatically upregulate miR-145 and inhibit PI3K/Akt/mTOR pathway to suppress the progression of LSCC.^312^

##### Alkaloids

Han et al. exhibited piperine-induced apoptosis of oral cancer cells is associated with the inhibition of PI3K/Akt/mTOR pathway with the expression levels of the autophagy-related proteins a significant decrease in p-mTOR, Beclin1, and LC3 in vitro, and inhibiting tumor growth, inducing apoptosis in vivo.^313^ Berberine dosed-dependently inhibited SW480 cells proliferation by inducing autophagy and cell cycle arrest under the regulation of PI3K/Akt/mTOR pathway by upregulating phosphatase and tensin homolog deleted on chromosome ten (PTEN).^314^

##### Terpenoids

Paclitaxel increases ROS-mediated DNA damage thus triggers the activation of apoptotic signaling pathways, and inhibits the epidermal growth factor receptor (EGFR)/PI3K/Akt/mTOR signaling pathway to prevent PC9 cell proliferation to exert anticancer effect.^315^ Andrographolide shows an inhibitory effect on the proliferation of MCF-7 cells through ERα-mediated transcription and undergoes crosstalk with PI3K/Akt/mTOR signaling in a concentration-dependent manner, and the effects is comparable to that of the anticancer drug fuvinsetron, indicating its potential role as a possible anti-estrogenic agent in the treatment of breast cancer.^316^ Ursolic acid administration can inhibit the proliferation and induce apoptosis in LNCaP and PC-3 cell lines through PI3K/Akt/mTOR pathway, as characterized by the increased Annexin V-binding.^317^ Moreover, administration of ursolic acid significantly inhibits the growth of LNCaP prostate tumor xenografts in vivo, which is confirmed to be related to the inhibition of the PI3K signaling pathway. Thus, ursolic acid appears to be an attractive natural compound for the chemoprevention of prostate cancer. The treatment of artemisinin can dramatically reduce the phosphorylation of PI3K, Akt, and mTOR in uveal melanoma cells, as well as induce mitochondrial membrane potential loss and apoptosis, demonstrating the therapeutic potential on primary intraocular malignancy.^318^

#### Wnt/β-catenin signaling pathway

Wnt/β-catenin signaling pathway, also known as the canonical Wnt signaling pathway, involves the nuclear translation of β-catenin and activation of target genes through T-cell factor/lymphoid enhancer-binding factor (TCF/LEF) transcription factors.^319–321^ Once the signaling pathway is activated, it will induce the stability of β-catenin and transfers it to the nucleus, ultimately promoting the expression of genes involved in cell proliferation, survival, differentiation, and migration.^319,322^ Moreover, cytoplasmic–nuclear shuttling of β-catenin is believed to be a prominent property of Wnt/β-catenin pathway activation.^323^ Various investigations suggested its dysregulation is one of the most relevant events related to the development of cancer.

##### Polyphenols

Quercetin can impair the increased expression of β-catenin and cyclin D1 induced by TGF-β in prostate cancer (PC-3 cells), and it has the potency to prevent TGF-β-induced epithelial-mesenchymal transition (EMT) process by suppressing N-cadherin and vimentin while increasing E-cadherin.^324^ Apigenin inhibits β-catenin/TCF/LEF signaling activation induced by LiCl in a dose-dependent manner, and restrains β-catenin nuclear entry, thereby inhibiting the expression of Wnt downstream genes, thus significantly inhibits the proliferation, migration, and invasion of CRC cells (HEK293T and SW480) and the growth of intestinal organoids.^325^ Apigenin can also suppress in vitro and in vivo HCC (HepG2 and SMMC-7721) growth by downregulating H19 and reducing β-catenin expression, leading to deactivation of Wnt/β-catenin pathway and its downstream genes octamer-binding transcription factor 4 (OCT4), vascular endothelial growth factor (VEGF), CD44, cyclin D1, and axis inhibition protein 2 (Axin2).^326^ EGCG can effectively diminish the spheroid formation of lung cancer and CRC, as well as the CSC markers, along with deactivating Wnt/β-catenin pathway to suppress lung cancer and CRC cell proliferation and induce apoptosis.^327,328^ In gastric cancer, EGCG decreases nuclear translocation of β-catenin, and downregulates its downstream gene, CCND1, c-Myc, and c-JUN, indicating EGCG restrained proliferation of gastric cancer cells by inhibiting activation of canonical Wnt/β-catenin signaling.^329^ The proliferation and invasion of NSCLC cells (95D and A549) can be retarded by curcumin via metastasis-associated protein 1-mediated inactivation of Wnt/β-catenin pathway,^330^ and the levels of lung CSCs markers of CD133, CD44, aldehyde dehydrogenas 1A1 (ALDH1A1), Nanog and OCT4 can be downregulated, as well as the tumorsphere formation and the number of CD133 positive cells.^331^ In liver cancer, curcumin treatment suppresses long intergenic non-coding RNA ROR (lincROR) expression, blocks the activation of Wnt/β-catenin signaling via downregulating downstream target genes of CD44, OCT3/4, CCND1, and c-Myc,^332^ sperm-associated antigen 5 expression can also be decreased resulted in the suppressed expression of β-catenin.^333^ The preventive effect of curcumin on CRC is mainly reflected in upregulating naked cuticle homolog 2, suppressing EMT and chemokine receptor 4, and inhibiting the invasion and metastasis, thus leading to the downregulation of key markers of β-catenin, axin, and TCF-4 associated with Wnt/β-catenin pathway.^334^ In another research, it was found that curcumin is able to retard CRC by inhibiting Wnt/β-catenin pathway via miR-130a, indicating miR-130a might serve as a novel target of curcumin for the treatment of CRC.^335^ In addition to exhibiting inhibitory effects on the aforementioned cancer types, curcumin can also inhibit HepG2 cell proliferation and induced apoptosis by downregulating glypican-3 expression and Wnt/β-catenin pathway,^336^ significantly suppressing the levels of Wnt3a, recombinant low density lipoprotein receptor related protein 6 (LRP6), p-LRP6, β-catenin, p-β-catenin, c-Myc, and surviving to induce apoptosis of gastric carcinoma cells (SNU-1, SNU-5, and AGS),^337^ and exerting protective effects on chronic tobacco smoke exposure mediated urocystic EMT and acquisition of bladder CSCs through inhibiting Wnt/β-catenin pathway.^338^ In the mechanism of treating gastric cancer with resveratrol, runt-related transcription factor 3 and caudal-related homeobox TCF 2 expression levels are upregulated to inhibit the proliferation, along with the restrain of β-catenin and TCF-4.^339^

##### Alkaloids

Berberine can also downregulate lincROR, inducing the inactivation of Wnt/β-catenin signaling in vitro and in vivo and leading to the CRC cell cycle arrest and apoptosis.^340^ Piperine inhibits the translocation of β-catenin to the nucleus and might suppress the binding of TCF/LEF to the DNA, thus suppresses Wnt/β–catenin pathway to show an anti-CRC effect.^341^

##### Terpenoids

Tong et al. reported artemisinin notably diminishes tumor growth in A549 xenograft model via downregulated the expression of Wnt5-a/b, LRP6, disheveled, dsh homolog 2 (Dvl2), and β-catenin (including nanog, sox2, OCT3/4, and cyclin D1).^342^ The findings of Mandal et al. indicated ursolic acid upregulates secreted frizzled related protein 4, and inhibited miR-499a-5p to inhibit Wnt/β-catenin signaling thus targeted breast CSCs.^343^ Li et al. explored the relationship between andrographolide and macrophage polarization in breast cancer.^344^ The in vivo studies displayed that andrographolide restrained the growth of MDA-MB-231 and HCC1806 human breast tumor xenografts and 4T-1 mammary gland tumors through tumor-associated macrophages, and this effect was closely related with the inhibition of Wnt5a/β-catenin pathway, indicated tumor-associated macrophages might be a potential novel therapeutic target for breast cancer.

#### NF-κB signaling pathway

There are two main pathways that regulate the transcriptional activity of NF-κB signaling proteins, with the first one was known as a canonical pathway, plays an important role in the control of innate immunity and inflammation.^345,346^ Compared to canonical pathway, the activation of noncanonical pathway is slow and persistent,^347,348^ and exhibits a crucial role in controlling the development, organization, and function of secondary lymphoid organs, as well as the maturation and survival of B cells.^345^ NF-κB pathway plays a vital role in inflammation-related diseases. It is suggested inflammation is a contributing factor to most solid and hematopoietic malignancies,^349^ and the activation of NF-κB has been proposed to be a major factor in linking inflammation and cancer development.^350^ The high expression level of NF-κB in cancer-related tissues leads to the aggregation of pro-inflammatory cytokines and the development of the tumorigenic microenvironment, which in turn cause the occurrence and development of tumors.^350,351^ In addition, NF-κB signaling also plays a central role in the metabolic response of tumor cells by coordinating metabolic processes, affecting glycolysis, glutaminolysis, etc.^352^

##### Polyphenols

Quercetin treatment provides chemoprevention on oral squamous cell carcinoma mainly by suppressing NF-ĸB signaling pathway via downregulating NF-ĸB p50 and p65.^353^ Snail is a critical transcription factor in regulating EMT in cancer, and patients with higher expression of Snail usually have shorter survival.^354^ Snail also can be regulated by NF-κB, both colon cancer cells and HCC cells have been found NF-κB/Snail signaling pathway can be inhibited by apigenin treatment.^355,356^ In addition, apigenin administration downregulated NF-κB transcription activity, inhibitor of NF-κB (IκB)-α phosphorylation, transcription of p65 and p50 to nucleus, and inhibitor of κB kinase (IKK)-β expression in pancreatic cancer cells and xenograft mouse model.^357^ EGCG inhibits bladder cancer SW780 cell proliferation and migration both in vitro and in vivo via downregulation of NF-κB p65 and MMP-9.^358^ Sah et al. further investigated the effect of EGCG on T24 cells, and it was found EGCG blocked the IL-1β stimulated ROS production, in turn restraining NF-κB signaling and anti-invasion effects by inhibiting the expression of urokinase-type plasminogen activator (uPA) receptor,^359^ the glycosylphosphatidylinositol (GPI)-anchored cell membrane receptors that have vital roles in cell invasion and metastasis of bladder cancer.^360^ By deactivating NF-κB p65, EGCG can repress nasopharyngeal cancer stem cell self-renewal and migration and reverses EMT.^361^ Marquardt et al. displayed the growth-suppressive effects of curcumin on hepatoma cells was dependent on the extent of NF-κB inhibition, and emphasized the potential of NF-κB targeting to effectively consume CSCs in liver cancer. Meanwhile, it is pointed out that HCC patients with poor prognosis may benefit from curcumin treatment, and specific disruption of NF-κB signaling might be a potential therapeutic method for HCC patients with poor prognosis.^362^ Curcumin can also modulate the dysregulation of miR-200c, miR-21, miR-let7c, miR-26a, and miR-125b in advanced thyroid cancer, which are associated with regulating cell differentiation and NF-κB activity, as well as decrease NF-κB p65 activity.^363^ Plenty of studies have shown autocrine growth hormone signaling cause EMT and trigger a metastatic profile by increasing occludin and fibronectin expression levels in breast cancer.^364,365^ With the treatment of curcumin, the invasion and metastasis of breast cancer cells can be inhibited by autocrine growth hormone-mediated targeting both canonical and noncanonical NF-κB signaling.^366,367^ In addition, curcumin can also play anticancer role against gastric cancer,^368^ pancreatic cancer,^369^ NSCLC,^370^ and CRC^371^ via regulating NF-κB signaling. VEGF is a key regulator of angiogenesis, and IL-8 is a regulation protein involved in tumorigenic activities in cancers, resveratrol can modulate activity of VEGF and IL-8 in SKOV-3 cell aggregates via significant attenuation of the expression of NF-κB, p-NF-κB, and proliferating cell nuclear antigen.^372^

##### Alkaloids

Li et al. demonstrated berberine can constrict the growth of HepG2 cells by promoting apoptosis through the NF-κB p65 pathway.^373^ Homoharringtonine exhibits an anti-inflammatory activity, and can attenuate dextran sulphate sodium (DSS)-induced colitis by inhibiting macrophage-associated NF-κB activation (downregulate p-p65 and p-IκBα) and M1 polarization, which could be an option for the treatment of ulcerative colitis (UC) or prevention of CRC.^374^ A unique molecular mechanism of homoharringtonine was reported by Chen et al. that by directly binding NF-κB repressing factor (NKRF), several NF-κB target genes including MYC, MMP can be regulated.^375^ By strengthens the interaction of p65-NKRF, and interferes with p65-p50 complex formation, homoharringtonine can attenuate the transactivation activity of p65 on MYC gene in acute myeloid leukemia (AML), moreover, the expression of a frequently mutated and/or highly expressed gene, named KIT, can be significantly diminished, which indicated patients with MYC and KIT overexpression could achieve a favorable response to homoharringtonine treatment.^375^

##### Terpenoids

Zhang et al. found andrographolide can inhibit proliferation of SW620 cells through the toll-like receptor 4 (TLR4)/NF-κB/MMP-9 signaling pathway of decreasing the expression of TLR4, MyD88, p65, and MMP-9.^376^ In another type of CRC cell of HCT116, andrographolide attenuated TNF-α-induced IL-8 via inhibition of NADPH oxidase/ROS/NF-κB signaling and then suppresses angiogenesis in tumor microenvironment.^377^ Andrographolide remarkably constricted the expression of p65 and p-p65 in MCF-7 cells and tumor tissues of MMTV-PyMT mice to deactivate NF-κB pathway to inhibit the expression of miR-21-5p, thus stimulating programmed cell death 4 expression to abate luminal-like breast cancer growth, metastasis, and invasion.^378^ Li et al. investigated the anticancer effect of ursolic acid in four types of gastric cancer cells including BGC-823, HGC-27, AGS, and MGC-803. Different types of cells have different responses to ursolic acid, the expression of N-cadherin, vimentin, Snail, Twist p-Axl, p-IKKα/β, and p-NF-κB were downregulated in BGC-823 and MGC-803 cells, meanwhile, the expression of N-cadherin, Snail, p-Axl, and p-IKKα/β were diminished in xenograft model rats, indicating the anticancer effect of ursolic acid was conducted by the attenuation of EMT, which was associated with the regulation of Axl/NF-κB pathway.^379^ Su et al. discovered artemisinin exert preventive effects on *H. pylori*-induced gastric cancer both in vivo and in vitro, these effects were closely related to the inhibition of NF-κB signaling of the decreasing expression of p-p65 and p-IκB-α, as well as restraining the downstream inflammatory factors of IL-8 and TNF-α.^380^

#### JAK/STAT signaling pathway

The JAK/STAT signaling pathway is considered as one of the central communication nodes in cellular function, it forms a rapid membrane-nucleus signal transduction module, and induces the expression of various key mediators in cancer and inflammation.^381–384^ Blocking the JAK/STAT signaling in cancer cells can inhibit the expression of target genes that control basic cellular functions and hinder cancer cells from escaping growth control mechanisms, thus, antagonizing JAK/STAT pathway might prevent the transformation of precancerous lesions into malignant tumors.^385,386^ In addition, plenty of studies have shown that activation of the JAK/STAT pathway plays significant role in the homeostasis of the immune system.^387,388^ Inhibition of JAK/STAT signaling on the restrain of pro-inflammatory responses and auto-immune conditions, appears to be a promising strategy for preventing disease progress, particularly in skin diseases.^389^

##### Polyphenols

Igbe et al. proposed quercetin stimulated the inhibitory effect of IFN-α on HCC (HepG2 and Huh-7) cells proliferation by inhibiting Src homology domain 2 containing tyrosine phosphatase 2 activation of the JAK/STAT pathway. Interestingly, only the tyrosine phosphorylation of STAT1, but not STAT3 can be significantly raised by quercetin.^390^ In addition, quercetin can also suppress p-JAK2 and p-STAT3 to deactivate JAK/STAT signaling in LM3 cells, as well as inhibit LM3 cell tumor growth in vivo.^391^ Ko’s team found apigenin can inhibit the proliferation of MDA-MB-453 cells by upregulating the levels of caspase-3 and caspase-8 to induce the cleavage of PARP, and blocking the activation of p-JAK2, p-STAT3, and the nuclear staining of STAT3.^392^ They further studied another breast cancer cell line of BT474, and found apigenin can also effectively inhibit its proliferation in a dose- and time-dependent manner through downregulating the expression of p-JAK1, p-JAK2, and p-STAT3 and decreasing the production of STAT3 target genes such as VEGF and MMP-9 to prevent or treat HER-2-overexpressing breast cancer.^393^ Torres et al. demonstrated EGCG transcriptional regulated ES-2 ovarian CSC molecular signature in tumorspheres and induced a pro-apoptotic phenotype.^394^ Further mechanistic research indicated EGCG mimic as a JAK/STAT3 inhibitor to regulate the acquisition of CSC phenotype and chemotactic response of ovarian cancer tumorspheres, which supported the chemopreventive benefit of EGCG in cancer.^394^ Liu et al. evaluated the potential chemoprevention and therapeutic effect of curcumin in esophageal squamous cell carcinoma (ESCC) patient-derived xenograft models.^395^ It was indicated that curcumin inhibited JAK2 and arrested the activation of STAT3, leading to diminished expression of STAT3-regulated genes and increased apoptosis in ESCC.^395^ Moreover, prophylactic administration of curcumin was significantly more effective in inhibiting 75% of patient-derived xenograft models than giving curcumin only after the innoculation of tumors, indicating curcumin might be an effective chemoprevention agent for ESCC. Curcumin might be a chemoprevention agent for CRC, as it can suppress the activation of dendritic cells by diminishing the phosphorylation of JAK2, STAT3, and STAT6, elevating the downstream proteins of suppressor of cytokine signaling (SOCS) 1, SOCS3, and protein inhibitor of activated STAT3 to restore immunologic balance and to treat colitis in an effective manner.^396^ Curcumin can inhibit the growth and recurrence of various tumors by adjusting JAK/STAT signaling pathway. In papillary thyroid carcinoma of BCPAP, TPC-1, and SW1736 cells, curcumin blocks G2/M phase, increases apoptotic rate, and downregulates p-JAK2 and p-STAT3 to inactivate JAK/STAT signaling.^397,398^ Curcumin suppresses invasiveness and vasculogenic mimicry of squamous cell carcinoma of the larynx through the inhibition the levels of JAK2, p-STAT-3, as well as endothelial nitric oxide synthase related with cell growth and apoptosis, MMP2 and VEGF associated with antiangiogenic.^399^ In osteosarcoma, the inhibition of the proliferation and migration of MG-63 cells by curcumin is through the restraining p-JAK2 and p-STAT3 to induce the arrest of the G0/G1 phase and apoptosis.^400^ In addition, curcumin can reduce fascin expression through inactivation JAK/STAT signaling to exhibit as a potential cancer chemoprevention agent for suppressing metastasis and recurrence of ovary cancer.^401^ Resveratrol alleviates inflammatory bowel disease (IBD) via the reduction of O-GlcNAcylation of STAT3 in intestinal epithelial cells, thus preventing its phosphorylation and abating the activity of the JAK2/STAT3 pathway.^402^ Resveratrol can also restrain the proliferation and invasive migration ability of GBM cells, as well as improve the inflammatory response of glioblastoma by inhibiting JAK2/STAT3 pathway and impairing the activation of NLRP3 inflammasomes.^403^

##### Alkaloids

Berberine inhibits JAK2/STAT3 pathway through reducing the expression of IL-6, thereby causing G0/G1 phase cell arrest and apoptosis in gastric cancer cell in vitro and in vivo.^404^ Through upregulation of miR-17-5p, berberine exhibits anti-bladder cancer effect via inactivating JAK1/STAT3 signaling. MiR-17-5p can directly bind to the 3’ UTR of JAK1 and STAT3, and block the expressions of JAK1, p-STAT3, and STAT3, thus provide supporting evidence for berberine to prevent the progression of bladder cancer.^205^ Homoharringtonine can prevent cells growth, cell viability, and induce cell apoptosis through mitochondria pathway in gefitinib-resistant NSCLC cell lines.^405^ Mechanistically, homoharringtonine retards IL-6-induced STAT3 tyrosine 705 phosphorylation and deactivates JAK1/STAT3 signaling.^405^ Wherein, cells with EGFR T790M mutation are more sensitive to homoharringtonine treatment.

#### Hippo signaling pathway

Hippo signaling plays a crucial role in regulating cell proliferation, differentiation, and survival, has been evidenced to contribute to the progression of various diseases, especially cancer.^406^ The Hippo pathway can be activated by a sequence of phosphorylation events. When cancer occurs, the Hippo signaling becomes inactive, the unphosphorylated yes-associated protein (YAP)/transcriptional co-activator with PDZ-binding motif (TAZ) enters the nucleus, and binds to the transcriptional enhanced associate domains (TEADs) transcription family, synergistically promoting the expression of target genes, enhancing cell proliferation, resistance to apoptosis, invasion and metastasis.^407,408^ In addition, multiple studies have demonstrated the role of the Hippo pathway in regulating host immunity,^409–411^ and it also has been proved to regulate the immune checkpoint molecule PD-L1.^412–414^ The development of targeted Hippo pathway for cancer chemoprevention and treatment by natural compounds is still in its infancy stages with a promising future.

##### Polyphenols

As a highly recurrent cancer, triple-negative breast cancer (TNBC) lacks effective prevention measures.^415^ Li et al. demonstrated the inhibitory effect of apigenin on stemness features of TNBC cells in vitro and in vivo.^416^ It was found apigenin protected organism by reducing the activity of YAP/TAZ and the expression of target genes such as connective tissue growth factor (CTGF) and cysteine-rich protein, angiogenic inducer 61 (CYR61), as well as corrupting the YAP/TAZ-TEADs protein-protein interactions.^416^ Apigenin reduces YAP expression to lessen migration and invasion by adjusting the expression of the EMT markers, and assisted the autophagy of HCC cells by modulating the expression of autophagy-related genes.^417^ As a common cancer type of head and neck, tongue squamous cell carcinoma can be induced to apoptosis by EGCG via declining the Hippo-related protein levels of TAZ, large tumor suppressor homolog 1 (LATS1), MOB kinase activator 1 (MOB1).^418^ Kruppel-like factor 5 (KLF5) has been reported to exert carcinogenic effects in various cancers.^419–421^ Curcumin behaves an effective suppression on tumor growth and pro-proliferative YAP/TAZ/KLF5/cyclin D1 axis, thus promoting KLF5 proteasome-dependent degradation in bladder cancer.^422^ Curcumin also play an anti-pancreatic cancer role by downregulating YAP and TAZ.^423^ In addition, curcumin can also inhibit autophagy in CRC and stemness in NSCLC by inhibiting nuclear cytoplasm translocation of YAP or TAZ alone, thus activating Hippo signaling.^424,425^ It was known persistent liver fibrosis can progress to HCC.^426^ Li et al. found resveratrol heightened the activation of Hippo signaling and attenuated the expression of YAP and TAZ to contribute the apoptosis of hepatic stellate cells.^427^ A novel mechanism was found by Xu et al. that ST6 beta-galactosamide alpha-2,6-sialyltranferase 2 (ST6GAL2) can inactivate Hippo signaling and promote tumorigenesis of follicular thyroid cancer, and resveratrol can reverse this process by suppressing the expression of ST6GAL2 and promoting p-mammalian STE20-like protein kinase (MST) 1/2, p-LATS1, and p-YAP, which provide a preventive possibility for patients with difficult thyroid cancer diagnosis.^428^ The cell cycle arrest and apoptosis of HCT116 cells can be also abated by resveratrol via upregulation p-YAP and downregulation of total YAP protein, and reduced the mRNA expression of the YAP signaling downstream genes CTGF and CYR61, which was partially mediated by the interaction between YAP and TEAD.^429^ Resveratrol exhibits a beneficial effects on breast cancer cell invasion by suppressing RhoA, leading to the activation of LATS1 and phosphorylation of YAP.^430^ Besides, resveratrol can also inhibit EMT in gastric cancer cells through Hippo–YAP pathway.^431^

##### Alkaloids

Homoharringtonine significantly enhances the phosphorylation levels of MST1/2, MOB1, LATS1, YAP, as well as salvador homolog 1 to activate Hippo signaling thus induce HCC cell cycle arrest at S phase and promote apoptosis.^432^ Ursolic acid diminishes the proliferation and metastasis of gastric cancer via the modulation of upregulating MST1, MST2, LATS1, and p-YAP through ras association (RalGDS/AF-6) domain family 1, suggesting ursolic acid might be a potential chemopreventive and therapeutic agent for gastric cancer.^433^ Li et al. disclosed artemisinin impaired mitochondrial respiration, induced ROS production, suppressed aerobic glycolysis, as well as regulated p-YAP and cytoplasmic retention thus inhibiting HCC cell growth, migration and invasion.^434^

#### Hedgehog pathway

The Hedgehog signaling pathway implicated in embryonic development, tissue patterning, and wound healing.^435^ Hedgehog signaling can divided into canonical and noncanonical pathway to elicit various cellular response. The canonical signaling pathway involves binding Hedgehog ligands to patched (PTCH) receptor to relieve the suppress of Smoothened transducer (SMO) by transmembrane receptor PTCH, and allowing SMO entering the primary cilium, thus activating lima-associated oncogene (GLI) transcription factors.^436^ In noncanonical Hedgehog pathway, it is largely attributed to crosstalk with other signaling cascades, partially by affecting the activity of GLI transcription factors.^437^ The imbalance of Hedgehog signaling pathway has been emphasized in cancer, and it is currently believed that one-third of malignant tumors rely on the abnormal function of this pathway.^436,438^

##### Polyphenols

Zhang et al. confirmed that genistein can inhibit cell proliferation, and induced apoptosis of nasopharyngeal CSCs via suppressing the tumorsphere formation capacity, decreasing the number of EpCAM^+^ cells, and downregulating the expression of nasopharyngeal CSCs markers, as well as suppressing Sonic Hedgehog (SHH) signaling mainly by downregulating the levels of SHH, SMO, and GLI 1.^439^ Genistein also shows an inhibition on renal CSCs through SHH pathway by diminishing SHH, SMO, GLI 1, and GLI2.^440^ In gastric CSCs, the levels of CD44, SHH, PTCH1, and GLI 1 are significantly reduced by genistein.^441^ It was considered that genistein could be an effect cancer therapy by modulating CSCs characteristic. The CSCs markers of CD44, CD133, OCT4, ALDH1A1, and Nanog were downregulated by EGCG, and the decreased levels of the components in Hedgehog pathway mediated the inhibitory effect of EGCG on bladder CSCs.^442^ This research team also studied the effect of curcumin on bladder CSCs. Curcumin can also suppress CSC properties through downregulating the expression of SHH, SMO, GLI 1, and GLI2 in SHH pathway thus diminishing the proliferation and enhancing apoptosis of bladder CSCs.^443^ Li et al. demonstrated curcumin decreased the expression of EMT and stemness in MDA-MB-231 mammospheres, which was related to the downregulation of GLI 1, GLI2, PTCH1, and SMO, especially GLI 1. Meanwhile, GLI 1 expression was significantly reduced in nucleus and cytoplasm, and it was found vimentin was interacted with GLI 1, which might be a downstream target of GLI 1 to suppress EMT and stemness.^444^ In addition, curcumin displays an important role in restraining hypoxia-induced pancreatic cancer metastasis via suppressing Hedgehog signaling pathway.^445^ It was reported resveratrol can reverse gastric cancer cell (SGC‑7901) proliferation, migration, invasive capacities, as well as EMT changes via the deactivation of Hedgehog pathway with the expression of decreased SHH, SMO, GLI 1.^446,447^ In pancreatic cancer of MIA PaCa-2 cell line, resveratrol induces apoptosis of MIA PaCa-2 cells in a dose-dependent manner, and the levels of Indian Hedgehog, PTCH, and SMO were reduced;^448^ in BxPC-3 and Panc-1 cell lines, resveratrol also suppresses hypoxia-induced expression of metastatic-related factors, uPA and MMP2, as well as markedly inhibits hypoxia-mediated activation of the Hedgehog signaling pathway via downregulating SHH, SMO, and GLI 1.^449^ Therefore, the changes in the same signaling pathway may vary among different cell lines of the same type of cancer, thus, research on multi-cellular lines is necessary. Resveratrol also has a good effect on improving gynecological tumors, such as ovarian cancer and cervical cancer. Ferraresi er al. displayed the significant role of Hedgehog downstream effector polycomb complex protein BMI-1 (BMI-1) in mediating lysophosphatidic acid pro-tumorigenic activities through inhibition of autophagy, and the capability of resveratrol to prevent Hedgehog pathway and BMI-1 activation to rescue autophagy and dampen the malignant features of ovarian cancer cells.^450^ The Cancer Genome Atlas data pointed patients with low expression of Hedgehog pathway/EMT-related genes have a better prognosis indicating resveratrol can as an adjuvant therapeutic for ovarian cancer.^450^ In cervical cancer, resveratrol inhibits the expression of SHH, SMO, GLI 1 to induce apoptosis, and constricts the migration and invasion of the HeLa cells.^451^ In addition, Hedgehog pathway can be inhibited by resveratrol via downregulating the protein of PTCH, SMO, and GLI 1 in a dose- and time- dependent manner in HCT116 cells.^452^ It was found resveratrol restrained cell proliferation and induced cell apoptosis of renal CSCs, as well as the Hedgehog pathway mediated the suppressive effects of resveratrol.^453^

##### Alkaloids

Research by Wang et al. showed that berberine significantly inhibited Hedgehog pathway in medulloblastoma, meanwhile, berberine cannot affect transcriptional factors activities induced by TNF-α and prostaglandin E_2_, indicated its selective activation of Hedgehog pathway. Furthermore, berberine inhibits the Hedgehog pathway by targeting SMO, the most successful molecular target for developing Hedgehog pathway anticancer drugs.^454^ It was reported berberine exerts strong potential in treating CRC with reducing the levels of SHH, PTCH1, SMO, GLI 1, enhancing the expression of suppressor of fused in vitro and in vivo, as well as retarding c-Myc downstream of Hedgehog signaling, and effectively improve the pathological profile of subcutaneous HCT116 xenograft tumor. The study also evaluates the toxicity of berberine on zebrafish, and points out that long-term and excessive consumption of berberine may cause cardiac and hepatic toxicity.^455^

#### Signaling pathway-associated crosstalk

Various studies have shown that no pathway exists independently, and direct regulation or mutual influence between signaling pathways and targets can enable cells to integrate different environmental signals and make more precise responses to meet the overall needs of the body.^301^

It was reported genistein can reduce the upregulation of interferon-γ (IFN-γ)/JAK1/STAT1 and INF-γ/toll-like receptor-4/NF-κB signaling pathways and modulate interferon regulatory factor-1/calcium-independent nitric oxide synthase/ nitric oxide (NO) and IL-6/JAK2/STAT3/COX-2 pathways and consequently, reduce the levels of TNF-α and IL-1β to preserve colon function, thus treating UC.^456^ In this study, genistein exhibited similar therapeutic effects as sulfasalazine (the agent most widely prescribed and standard therapy for IBD treatment), and the combination of the two agents yielded a superior benefit. Quercetin has been shown to suppress chronic stress-induced TNBC cell proliferation and migration by blocking the β_2_-adrenergic receptor/ERK1/2 pathway.^457^ Xia et al. demonstrated apigenin suppressed IL-1β-induced urokinase-type plasminogen activator receptor expression by inhibiting ERK1/2/JNK pathway and ERK1/2/JNK-dependent transcription factor AP-1 and NF-кB to exert anti-invasion effects in T24 cells.^458^ Kassouri et al. reported EGCG can induce transcriptomic program by increasing C-C motif chemokine ligand 5, cystatin 8, and interferon-induced transmembrane protein 3 characterized by reduced proliferative and stem-like features, which included the downregulation of Notch and CBFA2T3 pathways in macrophage-like differentiated human HL60 promyelocytic leukemia cells.^459^ Curcumin can effectively abolish the characteristic of lung CSC, and inhibit Wnt/β-catenin and SHH pathway to exhibit its prevention effect on lung cancer.^331^ Kurzava Kendall et al. found the epigenetic effects of resveratrol on oncogenic signaling in breast cancer. Resveratrol led to a DNA methylation increase within GLI2 and WNT4 enhancers, and the downregulation of CCND1 and CYR61, the common targets share by both of Hedgehog and Wnt signaling.^460^ The regulation of resveratrol on epigenetics may be a new strategy for the prevention of breast cancer. Li et al. verified berberine markedly decreased p-AKT1 expression and disturbances ERK/MAPK as well as p38 and JNK/MAPK pathways to inhibit migration and induce mitochondrial apoptosis in diverse thyroid carcinoma cells.^461^ In addition, berberine can also inhibit the progression of gastric cancer by regulating the crosstalk of adenosine 5‘-monophosphate (AMP)-activated protein kinase (AMPK) and Wnt signaling pathways.^462^ Berberine could downregulate hepatocyte nuclear factor 4α through activating AMPK signaling, and further downregulate Wnt5 and β-catenin to attenuate the growth, invasion and metastasis of gastric cancer. Cheng et al. determined homoharringtonine as an anti-STAT3 agent by regulating the crosstalk between STAT3 and Wnt signaling to achieve preventing CRC progression and recurrence.^463^ Homoharringtonine remarkably inhibited STAT3 expression and reduced epidermal growth factor-mediated β-catenin expression, leading to the inhibition of Wnt signaling to breakdown cancer stem-like tumorspheres in selective EGFR-positive CRC. Piperine can also prevent CRC by regulating the crosstalk between Nrf-2 and NF-κB pathways. Rehman et al. investigated prophylactic treatment of piperine can activate Nrf-2 pathway which triggers antioxidant response mediators of hemeo xygenase-1, glutathione, superoxide dismutase, etc., and block NF-κB signaling and its downstream molecules of COX-2, TNF-α, IL-6, etc. to prophylactic treatment of CRC.^464^ Ursolic acid can not only increase the activity of glycogen synthase kinase by downregulating PI3K/Akt signaling pathway, but also inhibit inflammation and prevent the progress of breast cancer by downregulating NF-κB signaling.^465^

In general, partial studies are limited to exploring the mechanism of natural compounds in cancer prevention from in vitro cell models, and we found the effects of the same natural compound on different type cell lines of the same cancer are not completely consistent, which may be due to different cell phenotypes. Further researches on multiple types of cell models should be strengthened. Besides, in vitro research cannot be limited to cell lines, as more complex in vitro cancer models have been developed, such as organ-chips, which can simulate the pathophysiology of cancer and conserve organ microenvironment, allowing researchers to study the effects of drugs on cancer in a controlled environment.^466^ With the continuous advancement of CRISPR-Cas technology, gene manipulation can now be performed on different types of organoids, and patient-derived organoids and organoids co-culture model systems can also as excellent model with tumor microenvironment.^467–470^ In addition, further in vivo studies are needed to verify the results, and currently, in vivo research usually adopt xenograft tumor mice as subjects, but far from perfect in vivo model. Moreover, current researches usually employed inhibitors or activators of a specific target for validation, which may not be able to remove interference factors from other signaling pathways. The technologies of CRISPR-Cas,^471^ site-specific recombinase,^472^ and induced expression (small molecule and virus) can more effectively capture human genetics within mice in a time-tunable and tissue-specific manner. Besides utilizing classical techniques, combining novel technologies and models will better facilitate the study of natural compounds mechanisms and discover key targets for cancer prevention.

### Targeting immune checkpoints

**Fig. 4:**
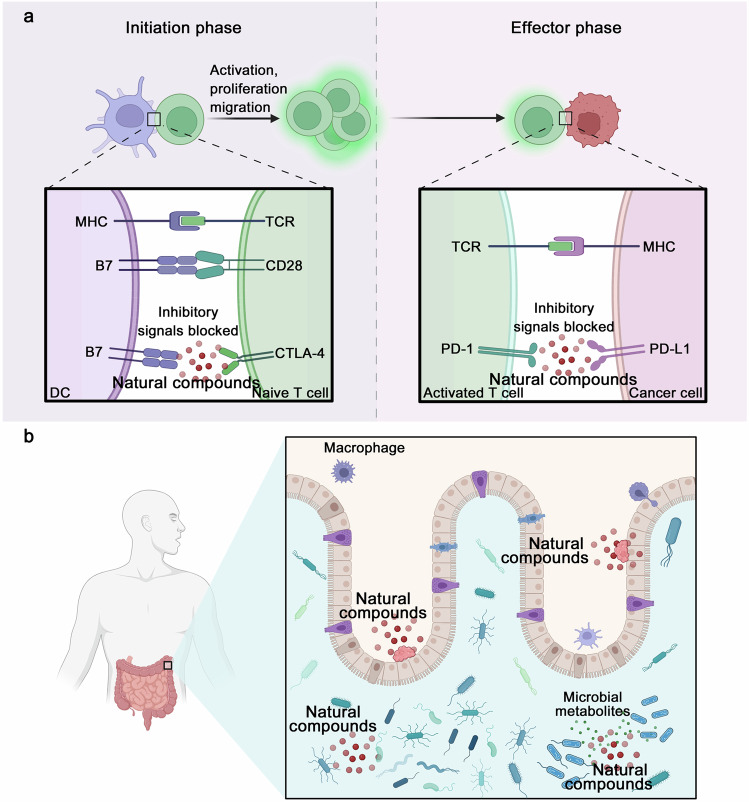
Natural compounds targeting immune checkpoints and gut microbiome to serve as cancer chemopreventive agents. **a** Targeting immune checkpoints. T cells recognize antigens presented by the major histocompatibility complex (MHC) on the surface of cancer cells through their T-cell receptor (TCR). However, this signal is not sufficient to initiate T-cell response and requires a second signal transmitted by the costimulatory molecules of B7. The interaction between cytotoxic T-lymphocyte-associated antigen-4 (CTLA-4) and costimulatory molecules mainly occurs during the initiation phase of T-cell responses. Programmed death-1 (PD-1) inhibitory receptors are expressed by T cells during long-term antigen exposure and exert negative regulation on T cells during their association with programmed death ligand-1 (PD-L1), and the PD-1 interaction occurs during the effector phase of T-cell responses. The inhibitory effect of natural compounds on tumor can be achieved by interfering with the interaction between PD-L1 or CTLA-4 with their corresponding receptors, and blocking the interaction of related signaling pathways, promoting T-cell activation, thus restoring immune cell function and promoting a strong tumor immune response. **b** Targeting gut microbiome. The imbalance of gut microbiota is closely related to the development of cancer, tumor growth may lead to local disruption of the barrier, resulting in microbial invasion and immune monitoring disrupted. The gut microbiome regulates various host processes, including metabolism, inflammation, and immunity. Natural compounds can improve cancer-related metabolism, inflammation, and immunity by remodeling gut microbiota homeostasis. DC dendritic cell. This figure was created with Biorender.com, and adapted from Antoni Ribas.^482^

Natural compounds can not only act on traditional signaling pathways, but also exert cancer chemoprevention by affecting immune checkpoints (Fig. 4a). Through the years, unsatisfactory outcomes of cancer treatment are partially due to systemic immune unresponsiveness or immunosuppression to cancer. The immune system dysfunction in tumors leads to malignant cells evading immune surveillance, immune recognition, and eradication.^473^ The emergence of immunotherapy provides a revolutionary new approach for the treatment of cancer. The purpose of cancer immunotherapy is to equip patients with cancer-fighting immunity, utilize their immune system to eradicate cancer and prevent recurrence.^474,475^ It was the emergence of immunotherapy that led to the 2018 Nobel Prize in Physiology or Medicine was awarded to Drs James P. Allison and Tasuku Honjo for their work in this field. Tumor immunotherapy mainly includes immune checkpoint inhibitors (ICIs), cellular immunotherapy, and tumor vaccines. Among them, ICIs are the most widely used tumor immunotherapy methods at present, and FDA has approved antibodies against programmed cell death-1 (PD-1), programmed death ligand-1 (PD-L1), and cytotoxic T-lymphocyte-associated antigen-4 (CTLA-4) for various solid tumors, which proved the effectiveness and prospects of immunotherapy as an anticancer approach.

#### Targeting the PD-1/PD-L1 axis

PD-1 is one of the co-inhibitory receptors expressed on T cells, B cells, and type 2 tumor-associated macrophages.^476^ PD-L1 is the ligand of PD-1, expressed widely on immune cells, tumor cells and other tumor microenvironment cells,^477^ and PD-1/PD-L1 axis plays crucial role in physiological immune homeostasis and is considered as a means for tumor cells to evade immune surveillance.^476,478–480^ An accumulating body of research indicates that the activation of PD-1/PD-L1 axis negatively modulates T-cell-mediated immune responses in peripheral tissues to protect tissues from immune-mediated tissue damage.^479,481,482^ Currently, nivolumab, pembrolizumab, tislelizumab, atezolizumab, durvalumab, and avelumab have been approved for clinical use in the treatment of melanoma, NSCLC, etc. Evidence suggests that natural compounds have potential immunomodulatory effects and can regulate the PD-1/PD-L1 axis.

Quercetin can highly binds with PD-L1 and inhibit the binding of glycosylated PD-1/PD-L1, thus promote the activation of T cells by blocking the interaction of PD-1/PD-L1, which shows exciting potential as a cancer chemopreventive agent.^133^ Xu et al. reported apigenin and curcumin significantly inhibited IFN-γ-induced PD-L1 upregulation, reduced p-STAT1, enhanced T-cell-mediated melanoma cell killing.^483^ In vivo studies have shown apigenin strongly suppressed tumor growth and boosted T-cell immunity via inhibiting PD-L1 expression in host dendritic cells.^483^ The dual effect of apigenin on the inhibition of PD-L1 expression provides new insights into the anticancer effect of apigenin and exhibits potential clinical significance. Lim et al. determined TNF-α is the main factor triggering cancer cell immune suppression against T-cell surveillance through stable PD-L1, and COP9 signalosome subunit 5 (CSN5) is a necessary factor in this process, with the ability to inhibit the ubiquitination and degradation of PD-L1.^484^ Besides, curcumin as a CSN5 inhibitor diminished PD-L1 expression and benefited anti-CTLA-4 therapy, suggesting curcumin might be a practical adjuvant to enhance immune-based therapies in breast cancer.^484^ Evidence suggests resveratrol act as inhibitor of glyco-PD-L1-processing enzymes to modulate N-linked glycan decoration of PD-L1, and interact with inner surface of PD-L1 to directly target PD-L1, which provide novel mean to restore T cells function.^485^ Resveratrol can also activate Sirtuin 1 deacetylase to deacetylate and stabilize the transcription factor Snail, which, in turn restrain the transcription of Axin2, thus lead to an enhanced binding of β-catenin/TCF and PD-L1 promoter, and display its role in inhibiting T-cell function in lung cancer.^486^ Liu et al. discovered berberine as a novel negative regulator of PD-L1 in NSCLC, it exerted antitumor effect through diminishing PD-L1 expression via specific binding to Glu76 of CSN5 and inhibiting its deubiquitination, as well as activating tumor-infiltrating T cells, which provided a theoretical basis for the potential application of berberine as a small-molecule inhibitor to disrupt PD-L1-mediated immunosuppression.^487^ Andrographolide diminishes PD-L1 expression via inducing p62-depending selective autophagy by modulating STAT3, besides, andrographolide potentiates the antitumor effect of anti-PD-1 mAb immunotherapy by stimulating the infiltration and function of CD8^+^ T cells.^488^ Kang et al. unveiled ursolic acid exhibited antitumor activity by repressing PD-L1 expression via EGFR/JAK2/STAT3 pathway, as well as downregulating the bindings of STAT3 to MMP2 and PD-L1 promoters in NSCLC.^489^

#### Targeting CTLA-4

CTLA-4 is also a classic immune checkpoint molecule, acting as a CD28 homolog, has a strong binding affinity to the ligands CD80 (B7-1) and CD86 (B7-2),^490,491^ thus affects T cells in the priming phase of T cells activation and decreasing T-cell immune response.^492,493^ In addition, CTLA-4 is also participate in other aspects of immune control, especially the regulatory T cells (Tregs) exhibit constitutive expression of CTLA-4, which is considered vital for their inhibitory effect.^493,494^ It was reported the decrease of CTLA-4 expression on CD4^+^CD25^+^ Treg with curcumin was associated with the declining of Treg-mediated inhibitory activity.^495^ Resvertrol can elevate the reduced Treg-related genes expression of CTLA-4 and TGF-β, thus restoring the function of Tregs.^496^

Immunotherapy has become a promising treatment method and has made exciting progress, but there still exist several problems. For example, the patient’s responsiveness is not high and there are fewer beneficiaries. Although patients have a positive response to immunotherapy, some patients might quickly develop resistance and experience pseudo-progression, and severe irAEs. In light of natural compounds that can act on immune checkpoints and have the advantage of reducing chemotherapeutic side effects, its combination with ICIs may enhance the efficacy and reduce related irAEs, thereby assisting in cancer chemoprevention.

### Gut microbiome regulation

With the expansion of research in the field of oncology, manipulating the gut microbiota to induce positive therapeutic effects in vivo has opened the door to novel technologies for cancer prevention and treatment. The gut microbiome regulates various host processes, including metabolism, inflammation, and immunity (Fig. 4b).^497–500^ Increasingly evidences indicated that the microbiome can also affect the development of cancer with the outcome may be positive or negative.^501–503^ Some microbes have been proven to have pro-tumorigenic effects, such as *H. pylori*, a primary carcinogen, contribute to gastric cancer and mucosa-associated lymphoid tissue lymphomas.^504,505^ *Fusobacterium nucleatum* and *Escherichia coli* are closely related to the onset and development of CRC.^506,507^ In addition, tumor growth may lead to local disruption of the barrier, resulting in microbial invasion, the pro-inflammatory and immunosuppressive effects of immune monitoring can be also disrupted.^497,508^

The gut microbiota plays crucial role in regulating cancer treatment outcomes, particularly in chemotherapy and immunotherapy.^509,510^ Chemotherapy drugs can reduce the diversity of the microbiome in the body by affecting host immune system, leading to a decrease in its therapeutic effects.^499^ While, the microbiome can also enhance the efficacy of chemotherapy drugs through metabolism, enzyme degradation, and immune regulation.^511^ Moreover, chemotherapeutic drug resistance is also related to the microbiota.^512^ Cancer immunotherapy has emerged a promising method for treating cancer patients, and there exists significant differences in the microbial diversity and composition of fecal samples between immunotherapy responders and non-responders, indicating that changes in clinical response may be related to the gut microbiome.^513,514^ Meanwhile, unremitting efforts were employed to predict immunotherapy responses through microbiome, and it helps in guiding the selection of appropriate cancer treatment methods.^515^ Hence, regulating microbiota and improving cancer-related microecological imbalances serve new strategies for preventing and treating cancer. It is worth noting that some natural compounds possess low bioavailability, but the influence of gut microbiota cannot be ignored when exploring their mechanisms of action.

Quercetin can enrich the abundance of *Akkermansia muciniphilia* to enhance the anticancer effect of cyclophosphamide, as well as increase the mobilization frequency of T cells and NK cells and reduce the frequency of Tregs during cyclophosphamide treatment, which indicates that quercetin can mobilize vital antitumor immune cells to enhance the antitumor effect of cyclophosphamide, reflecting the chemoprevention potential of quercetin in TNBC.^516^ Apigenin can effectively improve DSS-induced UC through regulating the abundance of *Akkermansia, Turicibacter, Klebsiella, Romboutsia*, etc., to reshape the disordered gut microbiota thus ameliorated colon injury, the expression of zonula occludens-1, claudin-1, and occludin were also upregulated to restore the integrity of the intestinal barrier.^517^ In further azoxymethane/DSS-induced CRC model, apigenin exhibits anti-CRC effect via influencing the abundance of *Firmicutes* and *Actinobacteria*.^518^ Furthermore, by reducing the gut microbiota and fecal transplantation, the role of apigenin in regulating gut microbiota was further confirmed. During the progression of colitis to CRC, curcumin reduces the burden of colon tumors, and increases the relative abundance of *Lactobacillales* and decreased *Coriobacterales* order.^519^ Resveratrol can influence UC mice of intestinal microfora, improve intestinal microbiota by increasing diversity and uniformity of cecal microbiota, thus exerting a protective effect on the intestines.^520^ The prevention and treatment mechanisms of berberine for CRC are currently relatively plentiful. Evidence show berberine recovers DSS-induced UC in a microbial-dependent manner, with it maintaining the structure and function of the intestinal mucosal barrier, regulating intestinal mucosal immune homeostasis, activating Wnt/β-catenin pathway,^521^ FXR/TGR5 signaling, increasing unconjugated bile acids and secondary bile acids in the gastrointestinal tract,^522^ activating aromatic hydrocarbon receptors^523^ to exert its protective effect in the colon. In further CRC mice model studies, berberine can still reduce the β-diversity of gut microbiota, enrich the probiotic community and reduce pathogenic microorganisms to relieve CRC progression, and the related fecal metabolism results also support the effect of berberine on gut microbiota.^524,525^ Berberine can also performance anti-breast cancer and anti-HCC role by regulating the abundance and diversity of microorganisms, as well as the production of microbiota-derived butyric acid.^526,527^ The prevention effect of ursolic acid on UC is related to abate the richness of gut microbiota and avoid the inflammatory response caused by intestinal epithelial barrier disruption.^528^

The preventive effect of gut microbiota on cancer is usually influenced by the interactions between microbiota, tumor, immune system, and metabolism. The flourishing development of next-generation sequencing and bioinformatics has led to a significant increase in research on the association between gut microbiota and cancer. However, most current researches only explored the impact of microbial communities on phylum or genus level, and have not used isolation and cultivation methods for in-depth research. Another controversial issue exists whether the fecal transplantation applied for manufacture gnotobiotic mice faithfully replicate the microbiota of donors? The future development of the microbiome is expected to identify individual microbial species that lead to cancer phenotypes and reveal their potential mechanisms, and to modify dominant microbiota for personalized treatment. Emerging spatial multi-omics technologies and organoid research can help uncover the aforementioned issues from genes to phenotypes. Microbiome research is in the ascendant, there are still mountains of black boxes to be opened for preventing cancer by revealing the impact of the microbiome on cancer hosts. The multiple advantages of natural compounds are constantly being exploited in regulating gut microbiota, and more and more candidate natural compounds that can serve as microbiota regulators to prevent the development of cancer.

## Drug delivery system assists in natural compounds-based cancer chemoprevention

**Fig. 5:**
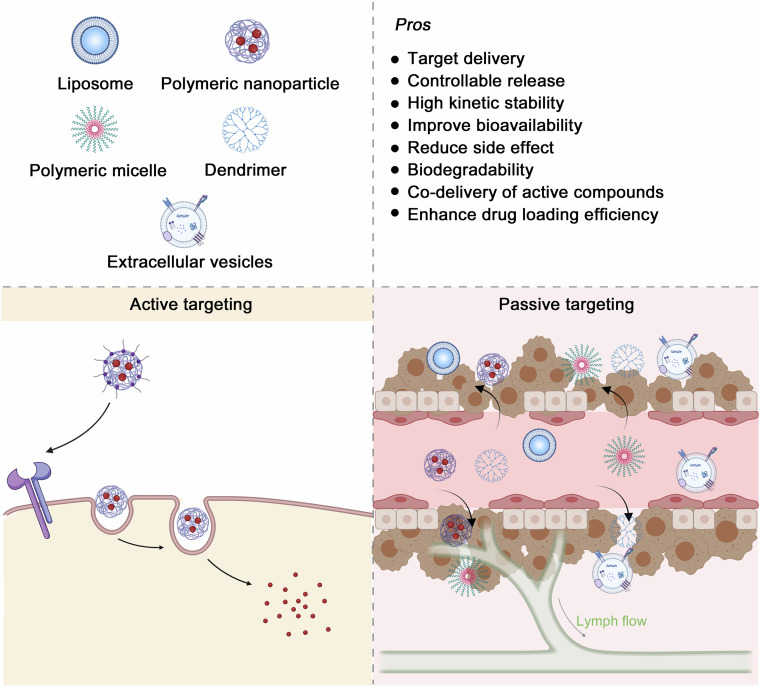
Drug delivery systems assist in natural compounds-based cancer chemoprevention. Diversity delivery platforms (mainly including but not limited to liposomes, polymeric nanocarriers, extracellular vesicles) enhance bioavailability and reducing toxicity of natural compounds, through active-targeting ligand decorated and passive targeting via the enhanced permeability and retention effect. This figure was created with Biorender.com, and adapted from Riya Khetan et al.^532^

Although these natural compounds exhibited various excellent antitumor effects in preclinical studies, most of them usually characterized as low bioavailability, poor targeting, and fast metabolic rate, which greatly hinder their further clinical application. With the introduction of novel drug delivery systems, drug delivery systems-chemoprevention therapies have established their potential with enhancing target ability, reducing side effects and drug resistance to provide better efficacy and safety, and has become a promising strategy for effective cancer treatment (Fig. 5).

### Nanotechnology polishing up for cancer chemoprevention

Loading natural compounds into a certain size of nanocarrier can alter the biological distribution of drugs, increase in vivo stability, prolong the blood circulation time, and achieve better accumulation of drugs at the tumor site through passive targeting, due to tumor angiogenesis with leaky blood vasculature, and by active targeting via binding to proteins overexpressed on the surface of tumor cells.^529,530^ By manipulating particle size and surface, controlling and sustaining release of drugs, siting specific targeting, using of multiple delivery routes of administration to improve the bioavailability, targetability, and controllability,^531–533^ which greatly improved the shortcomings of natural compounds in clinical applications, and amplified the pharmacological advantages. Currently, the widely studied nanomaterial-delivery systems of natural compounds mainly include liposomes and polymeric nanoparticles.

#### Liposomes

Liposomes are spherical vesicles composed of lipid bilayers, which creating two microenvironments, thus, lipophilic or hydrophilic molecules all can be encapsulated in the lipid bilayers or aqueous cores of liposomes.^534^ Due to the similarity in composition with the cell membrane, liposomes have the ability to diffuse across the cell membrane and have certain advantages as carriers for targeted drugs.^535^ Increasingly evidence suggests liposome drug delivery system increase anticancer activity,^536–538^ inhibit cancer cell proliferation,^539^ induce tumor cell apoptosis,^540,541^ etc. In particular, liposomes are considered an ideal carrier for mediating effective cancer immunotherapy by activating immune responses.^542,543^ Liposomal nanomedicine has been recognized as the most successful nanomedicine delivery system. The first liposome formulation to successfully obtain FDA approval for cancer treatment was Doxil^®^ (doxorubicin hydrochloride liposome), employed to treat ovarian cancer and AIDS-related Kaposi’s sarcoma.^544^ Doxil^®^ improved the pharmacokinetic properties of free doxorubicin and reduced its life-threatening toxicity.^544^ Marqibo^®^ (vincristine sulfate liposomes injection) was approved for the treatment of leukemia, which can reduce the severe neurotoxicity and gastrointestinal toxicity of vincristine.^545^ Jing et al. developed a folic acid-modified quercetin liposome that can further enhance the in vivo antitumor effect of quercetin.^546^ Zhang et al. prepared a dry powder of curcumin liposomes, which is more suitable for pulmonary delivery, and A549 cells showed significantly higher uptake of curcumin liposomes than free curcumin, and existed lower cytotoxicity to normal cells.^547^ Jhaveri et al. assembled transferrin moieties-modified resveratrol liposome to passive and active targeting treat glioblastoma. The modified resveratrol liposome exhibited more cytotoxic and induced higher levels of apoptosis accompanied by activation of caspases 3/7, and more effective to inhibit tumor growth and improve survival in mice than that of free resveratrol or resveratrol liposome.^548^

#### Polymeric nanocarriers

Polymeric nanoparticle is a class of polymer nanocarriers with conjugated or encapsulated drugs, exhibiting various structures. Polymer nanoparticles provide multifunctionality through the utilize of polymers with different chemical compositions, hydrophilic-lipophilic balance, charge and physical structures.^549^ In addition, the ability to control the degradation or decomposition of polymeric nanoparticles endows them with the ability to control drug delivery time over a wider range, which made polymeric nanoparticles attractive as therapeutic delivery carriers.^549^ The multiple surface functional groups of polymeric nanoparticles can be further modified by antibodies, peptides, and other targeting ligands, thereby promoting drug accumulation at the target site and mediating effective drug internalization in target cells, which can not only improve therapeutic efficacy, but also reduce potential adverse reactions of chemotherapeutics, and displayed great potential in preventing cancers.^550–552^ Abraxane^®^, also known as nab-paclitaxel, was consists of a nanosuspension of human serum albumin loaded with paclitaxel, used for the treatment of breast cancer, etc. The co-delivery polymeric nanoparticles of paclitaxel and curcumin exhibited an increasingly cellular uptake in breast cancer cells, and inducing cytotoxicity in breast cancer cells via the apoptotic pathway by blocking the G2/M phase of the cell cycle.^553^ In addition, polymeric nanoparticles can significantly inhibit tumor growth in vivo, prolong the survival time, reduce glucose metabolism in tumor tissues.^553^ Co-delivery of CPT and curcumin by cationic polymeric nanoparticles can also enhance synergistic effects to CRC.^554^

Polymeric micelles are composed of amphiphilic polymers, and can self-assemble in aqueous environments. These amphiphilic polymers are constructed by different polymer blocks, which can be optimized by adjusting the hydrophobic/lipophilic balance, size, drug loading capacity, micellar ability.^555–557^ Compared to other drug delivery systems, the size of polymeric micelles allows for more effective extravasation through the leaking vascular system, and the hydrophilic polymeric coating also prevents them from being recognized by the reticuloendothelial system during circulation and can be taken to the liver and spleen for clearance.^558^ Genexol^®^PM is the first successfully marketed polymeric micelles loading paclitaxel, it was approved for the treatment of metastatic breast cancer, etc.^559^ Apart from Genexol^®^PM, the number of successfully marketed polymeric micelles is limited, and although there are many ongoing clinical trials, the results have not yet been announced. Gong et al. prepared curcumin polymeric micelles and studied its anti-lung cancer effects, compared with free curcumin, the cellular uptake, in vitro cytotoxicity and anti-angiogenesis effect were increased in polymeric micelles, and continuous release behavior and slow extravasation behavior were also observed.^560^ Moreover, curcumin polymeric micelles were more effective in suppressing tumor growth and prolonging survival in LL/2 tumor models.^560^ The curcumin polymeric micelles were also be proven to be effective in preventing the development of CRC in vitro and in vivo mechanismly due to the inhibition of tumor proliferation and angiogenesis and increased apoptosis of tumor cells.^561^ The co-delivery of resveratrol and docetaxel via polymeric micelles can improve the treatment of drug-resistant tumors, and the effect is superior to the individual use of each drug.^562^ Besides, co-delivery of resveratrol and quercetin polymeric micelles can alleviate cardiotoxicity induced by doxorubicin.^563^

Dendrimers is a unique class of macromolecules with highly branched three-dimensional structures, and their shape and size can be precisely controlled, they exhibit an exponential number of dendritic branches radiating out from the central nucleus.^24,564^ It consists of three main components: a core or nucleus, an inner layer composed of repeating molecular units called dendrons, and the terminal functional groups on the surface.^565,566^ Dendrimers can carry bioactive compounds through covalent bonds or via ion interactions or adsorption within the internal space of nanostructures, thus demonstrating great potential as carriers for anticancer drugs.^565,567^ Gallien et al. encapsulated curcumin in surface-modified polyamidoamine dendrimers, and the in vitro studies have shown that the curcumin dendrimers effectively reduced viability of glioblastoma cell lines, while, unencapsulated curcumin was ineffective.^568^ Shen et al. developed a low-polyamidoamine dendrimers of ursolic acid conjugate modified with lactobionic acid, which could abate the migration and adhesion of SMMC-7721 cells by restraining metastasis-related protein MMP-9 expression, as well as effectively inhibit the tumor growth in H22 mice model.^569^ However, this study only used MTT assay as a detection method and only conducted cellular-level research, lacking in in vivo research results.

### Extracellular vesicles (EVs)

Although nanotechnology-based drug delivery systems have provided advantages for therapeutic delivery and achieved encouraging results, there are still many limitations in terms of biosafety issues and low biocompatibility. With the rapid development of the field of natural drug carrier systems, one of the most prominent examples is EVs. EVs are natural nano-size lipid bilayer vesicles secreted from all cell types,^570,571^ which can divide as three main subtypes based on their biogenesis: exosomes, microvesicles and apoptotic bodies.^572–574^ Exosomes have a size ranging from 40 to 160 nm (averaging 100 nm), and formed by invagination of the cytoplasmic membrane and inward of payload sprouts to form multivesicular bodies, and then fuse with the cell membrane and eventually release into the extracellular environment.^575^ Microvesicles (50–1000 nm) sprout outward directly from the plasma membrane, and apoptotic bodies (1000–5000 nm) are released by cells undergoing apoptosis through membrane foaming and rupture.^576^ EVs are biological origin membrane structures, and exhibit low toxicity, high biocompatibility, and low immunogenicity.^577^ In addition, due to its targeting ability to transfer bioactive molecules to target cells, it is beneficial to increase the concentration of drugs in target cells and reduce toxic side effects. Moreover, its lipid bilayer structure provide superior modification flexibility and biofilm permeability.^578^ Thus, EVs offer an alternative strategy and have become a promising vehicle for drug delivery, and engineered EVs, modified by genes or chemicals can enhance their targeting ability and loading efficiency, thereby improving the anticancer activity. EVs have been applied to deliver natural compounds to enhance their cancer prevention performance.

Paclitaxel can be loaded into EVs of various cells to enhance their cytotoxicity and reduce drug resistance. In a study, paclitaxel was loaded into human normal pancreatic duct cell line hTERT-HPNE-derived EVs and enhanced its effect in pancreatic ductal adenocarcinoma (PDAC) cells through a clathrin-dependent endocytosis pathway.^579^ Melzer et al. demonstrated bone marrow mesenchymal stroma/stem-like cell-derived EVs loaded with paclitaxel showed stronger antitumor effects than that of human umbilical vein endothelial cells loaded with paclitaxel.^580^ Wang et al. indicated the exosomes from M1-polarized macrophages act as carrier to deliver paclitaxel can enhance paclitaxel antitumor activity by activating macrophages-mediated inflammation.^581^ Chen et al. anchored ligands onto milk-derived EVs with the allowance of attachment of a substantial amount of transferrin, which significantly enhanced cellular uptake and induced pronounced cytotoxic effects when loaded with paclitaxel.^582^ Zhu et al. prepared c(RGDyK)-modified and paclitaxel-loaded embryonic stem cells–exosomes of cRGD-Exo-paclitaxel, the engineered exosomes exhibited more efficiently to glioblastoma versus free drug alone and drug-loaded embryonic stem cells–exosomes.^583^ Zheng et al. engineered exosomes derived from T cells expressing the chimeric antigen receptor (CAR-Exos), and paclitaxel was encapsulated into CAR-Exos (PTX@CAR-Exos) and administered via inhalation to an orthotopic lung cancer mouse model.^584^ The inhaled PTX@CAR-Exos accumulated within the tumor area, and prolonged survival with increased efficacy. In addition, it reprogrammed the tumor microenvironment and reversed the immunosuppression. Yang et al. developed a patient-derived exosome CPT delivery platform as personalized therapeutic modality for cervical cancer treatment. This therapeutic approach modulated the cell cycle and enhancing the sensitivity of tumor cells to radiotherapy, thus achieved significant antitumor effects.^585^

When curcumin was encapsulated into milk-derived EVs, it was shown enhanced antiproliferative activity against multiple cancer cell lines, especially enhanced higher antitumor activity in cervical tumor xenograft model.^586^ Jia et al. loaded superparamagnetic iron oxide nanoparticles (SPIONs) and curcumin into exosomes and then conjugated the exosome membrane with neuropilin-1-targeted SC peptide to obtain glioma-targeting exosomes.^587^ These exosomes provided good results for the synergistic anti-glioma effect with SPION-mediated magnetic flow hyperthermia and curcumin-mediated therapy. The engineered exosomes provide a potential pathway for improving the simultaneous diagnosis and treatment of gliomas. In addition, two clinical trials aim to encapsulate curcumin into plant-derived exosomes for the prevention/treatment of CRC (NCT01294072 and NCT04879810), but no data have been released. Deng et al. engineered human placental mesenchymal stem cell-derived exosomes loaded with berberine for UC therapy, and these exosomes concurrently demonstrated anti-inflammatory and antioxidant activities, contributing to the mitigation of UC with the possible mechanism of modulating MAPK signaling pathway, and the therapeutic effect was superior than that of berberine alone.^588^ Salek et al. loaded berberine to immature dendritic cells-derived EVs, and evaluated the antitumor activity and antiangiogenic properties in breast cancer cells and human umbilical vein endothelial cells.^589^ Berberine-loaded EVs upregulated the efficacy of free berberine in inhibiting cell proliferation, cell migration, capillary-like formation, and NO release to prevent tumorigenesis and angiogenesis. In summary, as a natural medicine delivery system, although there is currently limited clinical research on natural compounds-loaded EVs, multiple studies have shown exciting and encouraging results, and the potentiality of EVs is without doubt.

The rapid development of drug delivery systems can effectively improve the low bioavailability of natural compounds, while, there are still many obstacles from laboratory research to reliable and effective clinical applications. The current evaluation of cancer drug delivery systems mainly relies on xenograft tumor models, which cannot accurately predict the therapeutic effect of formulations in advanced cancer or metastatic tumors.^590^ The use of nude mouse models cannot evaluate the immune response against tumor cells or drug delivery systems.^591^ Therefore, using a spontaneous cancer model may be a better choice for evaluating drug delivery systems. In addition, studies have shown that nanotechnology-based delivery systems are more effective in small animal tumor models than in human tumors,^543,592^ and organoids can be attempted for evaluation. Cancers are closely related to the imbalance of the gut microbiota, and natural compounds can exert its effects by affecting the gut microbiota, therefore, targeted drug delivery systems should not be limited to protein ligands. Researches have shown that multiple parameters such as particle size, surface charge, particle shape, porosity, and biodegradability can affect the toxicity of nanotechnology-based drug delivery systems,^593^ thus, the biocompatibility, toxicology, long-term stability, and in vivo metabolic degradation pathways should be comprehensively evaluated.^535^ At present, the preparation of most nanotechnology-based formulations are completed in laboratories with sophisticated processes and difficult to apply in practice, which means the processes and technologies for large-scale production and quality control still need breakthroughs. In addition, the large-scale purification, reproducible production, optimal storage conditions (including storage buffer and storage container), and cycling stability for biological for natural drug carrier systems remain challenges. With the continuous advancement of precision medicine, drug delivery systems also need to be combined with personalized medicine. The addition of technologies such as gene editing and artificial intelligence (AI) may provide revolutionary changes for cancer prevention.

## Natural compounds improve the efficacy of chemotherapies

### Increasing chemotherapeutic sensitivity

Drug resistance remains a major obstacle in cancer prevention and treatment, and seeking strategies to overcome tumor resistance has always been a focus in the field of oncology. It is encouraging that natural compounds with different chemical structures and pharmacological effects are effective substances in assisting combat drug resistance.^594–596^ Currently, natural compounds as the fourth generation of multi-drug-resistant inhibitors have attracted the attention of researchers.^594^ Natural compounds reverse cancer multi-drug resistance mainly via regulating drug efflux, DNA repair, tumor microenvironment, and related signaling pathways.^597–599^

Quercetin can inhibit the activation of androgen receptors, PI3K/Akt signaling, and reduce the expression of hnRNPA1 and AR-V7, as well as P‑glycoprotein (P-gp) to enhance the sensitivity for prostate cancer.^600,601^ In addition, quercetin could enhance the therapeutic effect of conventional chemotherapy drugs on multi-drug-resistant BEL/5-fluorouracil (5-FU) cells overexpressing ABCB1, ABCC1 and ABCC2.^602^ Apigenin is an ideal adjuvant to improve doxorubicin resistance in breast cancer through regulating JAK2/STAT3/multi-drug resistance 1 (MDR1) axis to inhibit the expression of MDR1, multi-drug resistance-associated proteins, and P-gp.^603,604^ Curcumin can be used as a sensitizer to enhances the efficacy of gefitinib by inhibiting Sp1 and blocking its interaction with HADC1, downregulating EGFR activity, and this synergistic effect is confirmed as autophagy-dependent.^605^ In the study of drug resistance in CRC, curcumin modulates TET1-NKD-Wnt signaling to suppress the EMT progress, and also regulates CXC-chemokine/NF-κB signaling to sensitize drug-resistant cells to chemotherapies.^606^ By inhibiting the Akt cascade via activating PTEN, resveratrol attenuates invasive biological features, synergizes with doxorubicin to inhibit gastric tumor growth, and reverses its resistance by inhibiting EMT.^607^ It was reported andrographolide can target to Bax and trigger mitochondrial-mediated apoptosis, thus synergize the cytotoxic effect and reverse resistance of 5-FU on CRC.^608^ Ursolic acid restrains proliferation and reverses drug resistance of ovarian CSCs by downregulating ABCG2 via constricting the expression of hypoxia-inducible factor-1α.^609^ As a key mediator of drug resistance in breast cancer, HuR can be translocated to the nucleus by ursolic acid and decreased MDR1 expression.^610^

The sensitization mechanism of the same natural compound to chemotherapy drugs varies for different types of cancer cells, in addition to the heterogeneity of tumors, all of these hindered the positive outcomes of natural compounds. In light of the multi-target characteristic of natural compounds and its complex mechanism of action, future researches should shift the focus from in vitro studies to in vivo studies. Besides, in order to introduce more effective treatment methods for reversing tumor drug resistance into clinical practice, discovery of more effective and low toxicity ingredients is indeed crucial, which can be combined with AI-driven drug discovery, computer-aided drug design, high-throughput screening, etc. technologies to accelerate the development of active substances.

### Reducing chemotherapeutic side effects

Due to the limited specificity of chemotherapy drugs, cancer patients often experience adverse reactions or toxicity during treatment, including myelosuppression, gastrointestinal toxicity, cardiotoxicity, hepatotoxicity, neurotoxicity, ototoxicity, etc.^611–613^ Due to these side effects, patients may reduce the medication dosage or even stop treatment, leading to cancer metastasis or recurrence, seriously affecting their quality of life and survival time. Therefore, it is necessary to explore effective adjuvant strategies to prevent and reduce the side effects caused by chemotherapy. Numerous studies have shown that natural compounds can effectively attenuate the side effects caused by chemotherapy, which undoubtedly provides a potential means for preventing cancer progression.^614–617^

Cancer therapy has evolved from traditional therapies to the use of targeted therapy and immunotherapy. However, the cardiotoxicity associated with these therapies has always existed. Currently, dexrazoxane is the only FDA-approved option for preventing cardiotoxicity caused by anthracycline chemotherapy drugs.^618^ However, this is insufficient as dexrazoxane is not suitable for addressing all cardiotoxicity and may add additional issues to patient care and treatment.^619^ Curcumin, resveratrol, quercetin can significantly reduce the levels of cardiotoxicity markers (creatine kinase and lactate dehydrogenase, etc.) by increasing antioxidant capacity, protecting the myocardium from damage caused by chemotherapy drugs.^134,620–622^ He et al. proposed ferroptosis is involved in the occurrence of doxorubicin-induced cardiotoxicity, EGCG treatment can effectively inhibit oxidative stress and abnormal lipid metabolism, and maintain mitochondrial function by upregulating AMPKα2 and activating adaptive autophagy, thereby reducing doxorubicin-induced cardiotoxicity.^623^ Moreover, this protective effect is similar to that of the iron chelator dexrazoxane, the only drug approved by the FDA for the clinical treatment of doxorubicin-induced cardiotoxicity.

A chemotherapy drug is usually not limited to producing only one type of side effect, gastrointestinal toxicity, myelosuppression, and nephrotoxicity are also adverse reactions usually caused by chemotherapy drugs. 5-FU is the first-line chemotherapeutic for treating CRC, and the mucosal immune damage increases the possibility of secondary pulmonary infection in the host.^624^ Curcumin can also protect intestinal mucosa via IL-6/STAT3 signaling pathway.^625^ Quercetin and its nano-emulsion formulation were also reported to inhibit 5-FU-induced intestinal mucositis by suppressing the formation of ROS, downregulating the expression of NF-κB and hypoxia-induced factor-1α.^626^

Cisplatin is widely used for the treatment of various solid tumors, and mainly cleared by kidney through glomerular filtration and tubular excretion, thus easy to accumulate in the kidney and lead to nephrotoxicity, which cause negative impacts on clinical outcomes.^627,628^ Quercetin attenuates cyclophosphamide-induced nephrotoxicity via modulating the crosstalk of MAPK/ERK and NF-κB signaling.^629^ EGCG also demonstrates its chemopreventive effect on cisplatin-based ototoxicity as it can restore hearing and outer hair cells in the basal region of the cochlea, mechanismly associated with inhibition of ERK1/2, STAT1, and STAT3.^630^ Curcumin can also prevent cisplatin-induced nephrotoxicity by improving mitochondrial bioenergetics, ultrastructure, redox balance, and SIRT3 levels.^631^ In addition, curcumin can effectively reduce the concentration of cisplatin in the kidney by improving uric acid, plasma phosphorus, urinary creatinine, and creatinine clearance rate.^632^ In this study, the researchers also compared the preventive effect of melatonin on cisplatin-induced nephrotoxicity. Although there was no statistical analysis of the therapeutic effects of curcumin and melatonin, it can be found from the comparison of the above data results that melatonin has a weaker renal prevention effect than curcumin. Moreover, the combined use of curcumin and melatonin has a better preventive effect than the use of curcumin or melatonin alone. Besides, curcumin can not only prevent nephrotoxicity caused by cisplatin, but also alleviate ototoxicity by targeting p-STAT3 and Nrf-2 signaling.^633,634^

A number of studies disclosed the benefit of natural compounds for the treatment of tumors, which significantly reduce the side effects caused by chemotherapy drugs. Moreover, these natural compounds have almost no serious side effects and are relatively inexpensive and easy to obtain. However, most current studies did not set up a positive group for effective preventive drugs, making it impossible to evaluate the efficacy of natural compounds compared to existing drugs. Although natural compounds have made great progress in preventing and adverse reactions induced by chemotherapy agents, more clinical studies are needed to further confirm their preventive effects on side effects. In addition, reliable biomarkers are wanted for early identification and monitoring of toxic side effects of chemotherapy agents.

### Development of resistance against natural compounds

Despite the important role of natural compounds in cancer prevention and therapy, phenomena of resistance have already occurred. The current research on natural compounds resistance is mainly focused on marketed drugs, including CPT, vinblastine, and paclitaxel. One of the direct mechanisms for developing drug resistance is to increase the expression of ATP-binding cassette transporter family proteins, such as P-gp, breast cancer resistance protein (BCRP) to enhance drug efflux, the overexpression of efflux transporters result in the efflux of paclitaxel, vinblastine, vinblastine, and CPT from cells, thereby reducing therapeutic efficacy.^635–638^

Metabolizing enzymes of Cytochrome P450 (CYP) enzymes contributes to drug metabolism, and invasive cancer cells typically exhibit high levels of CYP. CYP3A4 and CYP2C8 are closely related to paclitaxel resistance.^639,640^ High levels of CYP enhance the metabolism of paclitaxel, reduce its concentration in cancer cells, thus develop drug resistance, and limit therapeutic efficacy. EMT is associated with cancer metastasis and along with invasion, it also participates in drug resistance.^641,642^ It was reported in A549 cells resistant to paclitaxel, Cathepsin L upregulated the expression of EMT-associated transcription factors Snail, Slug, zinc-finger-enhancer-binding protein (ZEB) 1, and ZEB2, downregulated E-cadherin to promote EMT.^643^ Hypoxia as an environmental stress, can activate tumor cell-related genes, and accelerate angiogenesis and invasion.^644^ Hypoxia induced upregulation of intracellular and extracellular gp96 and increased paclitaxel resistance, and accelerated EMT.^645^ In addition, a series of signaling pathways involved in tumor biological processes are also associated with drug resistance. Paclitaxel, vinca alkaloids have been shown to activate NF-κB pathway through downregulating IκBα and promoting the nuclear translocation of NF-κB thus contributing to chemoresistance.^646,647^ Activated PI3K/Akt signaling often contributes to the increased resistance in cancer cells,^648^ and miR-20a and miR-200c were found to upregulate the levels of Akt1 and p-Akt1 to activate PI3K/Akt pathway, thus contributing to paclitaxel resistance.^649^ Downregulation of Notch signaling could also inhibit the EMT process and increase the sensitivity of cervical cancer cells to paclitaxel.^650^ Park et al. demonstrated the prolonged stress associated with paclitaxel treatment stimulates p38 MAPK/p53 network and induces the transcription of EGFR, which activates the EGFR pathway and paclitaxel resistance in NSCLC.^651^ Brosseau et al. reported the overexpression of YAP/TEAD signaling was involved in the resistance to paclitaxel chemotherapy in lung cancer.^652^

Besides, autophagic flux can be driven by paclitaxel to promote resistance in ovarian cancer,^653^ and non-coding RNA can regulate resistance, such as lncRNA H19,^654,655^ MA-linc1,^656^ lincROR,^657^ lncRNA CCAT1,^658^ NONHSAT141924,^659^ miR34a,^660^ miR135b,^661^ miR-155,^662^ and miR199a.^663^ Here, we mainly lists the resistant mechanism of CPT, paclitaxel, and vinca alkaloids, due to the fact that most natural compounds have not been used in clinical for cancer therapy, and current research typically focuses on alleviating resistance with other chemotherapy drugs. With the continuous use of these natural compounds, their resistance might be overcome through the following strategies. The use of inhibitors targeting drug resistance, such as cyclosporine A, can inhibit the function of P-gp, reduce natural compounds being pumped out of cells by P-gp, increase intracellular drug concentration, and enhance the effect on drug-resistant cells. Verapamil and elacridar, the ABCB1 inhibitors, can also reverse resistance through reducing the efflux of drugs.^664^ The combination of different natural compounds or chemotherapeutics may enhance the inhibition of cancer cells by targeting multiple targets, thereby overcoming the resistance of a single natural compound. Natural compounds processed by drug delivery systems is prone to be effectively delivered into cancer cells thus overcoming resistance.^665^ Moreover, drug delivery systems can achieve targeted delivery, increase local drug concentration, and enhance the effect on drug-resistant cells. It is also possible to reduce drug resistance by improving the physicochemical properties of natural compounds processed by drug delivery systems, made it prone to be effectively delivered into cancer cells thus overcoming resistance, such as preparing them as prodrugs or modifying their structures to enhance their absorption and distribution.

## Natural compounds serving as drugs against cancers in clinical

### The current clinical research status of cancer chemoprevention with natural compounds

The potential applications of various natural compounds in preventing cancer have been extensively studied. Increasingly in vitro and in vivo data indicate the excellent efficacies on cancer prevention, prompting scientists to conduct clinical trials. However, currently, few clinical studies using natural compounds alone as a preventive agent, mostly in combination with natural compounds or in combination with chemotherapy drugs. Moreover, these studies mainly focused on the pharmacokinetics, efficiency, and safety of natural compounds. At present, there are a large number of natural compounds-related studies registered on ClinicalTrials.gov, but most of the completed projects have not published their results, and most of the studies are in phase I/II clinical trials, with only a few studies in phase III. Therefore, there is still a long way to go in verifying the chemopreventive effect of natural compounds, as not all results indicate that natural compounds is beneficial for cancer chemoprevention. The existing clinical studies on natural compounds have many common issues: small sample size, not the multicenter clinical research, single-arm or phase II trial without a comparator, lack of follow-up studies or short follow-up time, or the dosage may be ineffective. In addition, the patient is unwilling to undergo invasive or radiation-damaging examinations such as colonoscopy, gastroscopy, computed tomography, etc. at the end of treatment. These factors constrain the true efficacy of natural compounds, and with people’s understanding and attention to health, these issues will be overcome in future. In the next section, we listed the clinical research with research results in the past decade (Table 2).

**Table 2:** Clinical trials of natural compounds in cancer prevention

Natural compound	Register trial code	Phase	Therapeutic modalities	Participants enrolled	Status	Study Type	Reference
Curcumin	IRCT20180802040678N1	III	Used alone	70	Unknown	Interventional	^666^
Curcumin	NCT03211104	Unknown	Used alone	97	Completed	Interventional	^667^
Curcumin	NCT00641147	II	Used alone	44	Completed	Interventional	^668^
Curcumin	NRC-9626	Unknown	Used alone	70	Unknown	Interventional	^671^
Berberine	NCT02226185	II/III	Used alone	1108	Completed	Interventional	^673^
Genistein	NCT01985763	I/II	Combination therapy	14	Completed	Interventional	^674^
EGCG	NCT01481818	I	Combination therapy	24	Unknown	Interventional	^676^
EGCG	NCT02577393	II	Combination therapy	83	Completed	Interventional	^677^
EGCG	NCT01481818	II	Combination therapy	51	Unknown	Interventional	^678^
EGCG	NCT01481818	I	Combination therapy	20	Completed	Interventional	^679^
EGCG	NCT02580279	II	Combination therapy	180	Completed	Interventional	^680^
Curcumin	NCT01320436	III	Combination therapy	50	Completed	Interventional	^681^
Curcumin	NCT00192842	II	Combination therapy	17	Completed	Interventional	^682^
Curcumin	UMIN ID 000001386	I/II	Combination therapy	21	Unknown	Interventional	^683^
Curcumin	Unknown	Unknown	Combination therapy	160	Unknown	Interventional	^684^
Curcumin	Unknown	I	Combination therapy	14	Unknown	Interventional	^685^
Curcumin	NCT01859858	I	Combination therapy	28	Completed	Interventional	^686^
Curcumin	NCT03072992	II	Combination therapy	150	Completed	Interventional	^687^
Curcumin	Unknown	II	Combination therapy	52	Unknown	Interventional	^688^
Resveratrol	NCT01317199	I	Combination therapy	14	Completed	Interventional	^690^
Resveratrol	NCT01317199	II	Combination therapy	125	Completed	Interventional	^691^
Resveratrol	ANZCTR number: 366895	Unknown	Combination therapy	31	Unknown	Interventional	^692^
CPT	NCT02010567	I/II	Combination therapy	32	Terminated	Interventional	^693^
CPT	NCT02187302	II	Combination therapy	115	Completed	Interventional	^694^
CPT	NCT03531827	II	Combination therapy	4	Terminated	Interventional	^695^
Vinblastine	NCT00808639	II	Combination therapy	39	Completed	Interventional	^696^
Vinblastine	NCT01812369	III	Combination therapy	500	Unknown	Interventional	^697,698^
Homoharringtonine	Unknown	Unknown	Combination therapy	29	Unknown	Observational	^699^
Homoharringtonine	ChiCTR-IPR-15006816	Unknown	Combination therapy	1258	Unknown	Interventional	^700^
Homoharringtonine	ChiCTR2000029841	II	Combination therapy	21	Unknown	Interventional	^701^
Homoharringtonine	NCT04424147	II	Combination therapy	96	Completed	Interventional	^702^
Homoharringtonine	NCT04424147	II	Combination therapy	58	Completed	Interventional	^703^
Homoharringtonine	NCT04248595	II	Combination therapy	20	Unknown	Interventional	^704^

### Clinical trials with a single natural compound on cancer

#### Curcumin

In a randomized double-blind placebo-controlled trial, patients with liver cirrhosis were randomly divided into two groups to receive 1000 mg/day curcumin or placebo for 3 months, beneficial effects of curcumin supplementation were observed in downregulating disease activity scores and severity of cirrhosis in patients.^666^ Although it provided evidence that curcumin has a beneficial effect and has the potential to prevent the progression of liver cirrhosis, and reducing the development of liver cancer, this study only used liver enzyme activity to evaluate liver status, without conducting computed tomography, magnetic resonance imaging, or liver biopsy. Besides, it is reported oral administration of curcumin for 6 months (1440 mg/day) did not significantly affect the overall cessation time of intermittent androgen deprivation in prostate cancer patients, but the intake of curcumin significantly inhibited the elevation of prostate-specific antigen (PSA) during this period, indicated curcumin has potential beneficial effects on prostate cancer patients.^667^ Although this study failed to demonstrate the difference of the off-treatment duration in the curcumin group, it is the first clinical study to use curcumin alone on prostate cancer. If the dosage and duration of curcumin administration were increased in this study, better results might be achieved. Cruz-Correa et al. evaluated the efficacy and safety of curcumin in treatment of intestinal adenomas in 44 patients with FAP, patients were randomly assigned to curcumin group (3000 mg/day) and placebo group, and continued to receive treatment for 12 months.^668^ At the end of treatment, there was no significant difference in the mean number of polyps between curcumin group (22.6; 95% CI, 12.1–33.1) and placebo group (18.6; 95% CI, 9.3–27.8); so did the polyp size between the curcumin group (2.3 mm; 95% CI, 1.8–2.8) and the placebo group (2.1 mm; 95% CI, 1.5–2.7). The insufficient dosage of curcumin might be the main factor leading to insufficient regression of adenomas. While it is gratifying that after 12 months of treatment with curcumin, the tolerance was good and there was no significant difference in the incidence of adverse events compared to the placebo group, indicating the safety of long-term use of curcumin. UC and Crohn’s disease is a risk factor for CRC.^669,670^ Sadeghi et al. conducted a randomized double-blind clinical trial to assess the preventive effect of curcumin, 70 patients with mild-to-moderate UC were randomly assigned to curcumin (1500 mg/day) or placebo intake for 8 weeks, and it was found taking curcumin supplements combined with medication treatment can significantly improve clinical outcomes and quality of life in patients with mild-to-moderate UC.^671^

#### Berberine

It is widely accepted that adenoma-carcinoma sequence represent the roadmap of most CRC arise.^672^ A multicenter, double-blinded, randomized controlled study was identified orally administrated with 0.3 g berberine twice a day was safe and can be effectively reduce the risk of recurrence of colorectal adenomas, and can be used as an option for postoperative chemoprevention after polypectomy.^673^ During the 2 years of follow-up, 155 (36%) participants in the berberine group and 216 (47%) in the placebo group were found to have recurrent adenoma (relative RR, 0.77; *P* = 0.001), revealed berberine reduced the risk of recurrence.

### Application of natural compounds in clinical combination/adjuvant therapy for cancer prevention

#### Genistein

Given the anti-CRC activity of genistein, a phase I/II pilot study was executed to estimate the safety and tolerability of genistein in combination with FOLFOX or FOLFOX-bevacizumab.^674^ In the 13 evaluable patients, RR in cycle 6 was 46%, with best overall response of 61.5%, median PFS of 11.5 months, and 15.4% patients were found in progression of disease. Seven of these patients continued to undergo surgical resection of the primary tumor (if not previously removed) and metastatic disease. In a previous study, the PFS was reported to be 9.4 months in chemotherapy combined with bevacizumab, and 8.0 months in the chemotherapy group alone,^675^ it suggested genistein in combination with chemotherapy may have potential to improve the therapeutic efficacy and chemoprevention of CRC.

#### EGCG

Acute radiation-induced esophagitis (ARIE) is a major dose-dependent side effect in unresectable stage III NSCLC and esophageal cancer patients receiving radiotherapy and chemotherapy, which may lead to unplanned treatment interruptions, and severe reduction in tumor control and survival rates. A phase I study of EGCG therapy protection of the esophagus from damage induced by chemoradiotherapy displayed 22 NSCLC patients had a rapid regression of esophagitis to level 0/1 in 24 patients within 2 weeks, the pain score also decreased from an initial average of 4.58 to 1.13 per week.^676^ In further phase II study, EGCG administration was found to be superior to traditional therapeutic applications in preventing ARIE, and the average adjusted esophagitis index of prophylactic EGCG (3.56 ± 2.90) was significantly lower than that of therapeutic EGCG (5.19 ± 2.73), indicated the prophylactic application of EGCG had an advantage over therapeutic use in adjuvant therapy for lung cancer.^677^ In another single-arm study related to esophageal cancer, the ARIE exhibited significant declines after EGCG treatment.^678^ Radiation-induced oral mucositis has a dismal outcome with limited treatment options, and EGCG mouthwash can effectively alleviate the integrity of oral mucosa in patients receiving head and neck radiotherapy.^679^ The 20 enrolled patients did not develop grade 3 or higher mucositis, and the score of mucositis-related pain was significantly reduced after administration EGCG mouthwash, and the recommended dose is determined to be 1760 μmol/L. Radiation dermatitis (RID) is the most common adverse event of radiotherapy for breast cancer, which may lead to the interruption of treatment, and then affect the progress of cancer. A phase II clinical trial enrolled 180 breast cancer patients of whom 165 [EGCG (660 μmol/L), *n* = 111; placebo, *n* = 54] were evaluable for efficacy.^680^ The occurrence of grade 2 or higher RID was significantly lower in the EGCG group (50.5%; 95% CI, 41.2–59.8%) than that in the placebo group (72.2%; 95% CI, 60.3–84.1%). This study once again confirms that prophylactic use of EGCG is an effective adjuvant therapy for cancer.

#### Curcumin

In a study of 50 mesalamine-treated patients with active mild-to-moderate UC, addition of curcumin to the treatment group resulted in better induction of clinical and endoscopic relief compared to the placebo group with no adverse reactions, indicated curcumin can be a safe and promising UC treatment drug and cancer chemopreventive agent.^681^ In addition, multiple oncology clinical studies have explored curcumin can be safely administered with standard chemotherapy agents such as gemcitabine,^682,683^ 5-FU,^684^ docetaxel,^685^ etc. Gbolahan et al. investigated the combination of curcumin with irinotecan which share the same metabolic enzymes of UDP-glucuronosyltransferases.^686^ It was found the combination of up to 4 g of Meriva^®^ (lectinized curcumin) and intravenous injection irinotecan was well-tolerated, and there was no increase in irinotecan-related adverse reaction. Another parallel-group comparative clinical study was designed to estimate the efficacy and safety of curcumin (CUC-01, 300 mg) combined with paclitaxel as compared to paclitaxel plus placebo in patients with metastatic and advanced breast cancer.^687^ After 12 weeks of treatment, a significant difference in the auxiliary analysis of the change in Response Evaluation Criteria in Solid Tumors (RECIST) score and ORR (51% vs. 33%) compared to that in the placebo group, indicated the greater benefit than the placebo. The median PFS in the curcumin group (27.0 weeks) was 2.4 weeks longer than that in the placebo group (24.6 weeks). In addition, a slight beneficial effect on reducing fatigue was also be discovered. Pastorelli et al. conducted a phase II study to investigate Meriva^®^ as complementary therapy of advanced pancreatic cancer of gemcitabine.^688^ It was found the disease control rate was 61.3% with a median PFS of 8.4 months, and a median OS of 10.2 months (95%CI, 8.8–11.7). The OS observed in this study was higher than that observed with gemcitabine alone (OS, 5.9 months),^689^ suggesting the complementary adminstration of phytosome complex of curcumin could increase the therapy of gemcitabine in advanced pancreatic cancer.

#### Resveratrol

Paller et al. conducted a phase I study of MPX (containing ellagic acid, quercetin, and resveratrol) on patients with biochemically recurrent prostate cancer (BRPC) to estimate its safety, tolerability, and dose determination.^690^ The cohort (*n* = 14) had a median follow-up of 19.2 months, and suggested 4000 mg of MPX was safe for future research. In the phase II trial of MPX in BRPC, 125 patients form multi-cancer were treated for at least 6 months.^691^ Although MPX did not significantly prolong prostate-specific antigen doubling time (PSADT) in BRPC patients, it is worth noting that PSADT pre-to-post increase was significant in the 27 genotyped patients with SOD2 Alanine/Alanine genotype (rs4880 T > C polymorphism) of MPX group (6.4 months), with the control group of 1.8 months. In another 12 month randomized pilot study of combination phytotherapy (containing turmeric, resveratrol, green tea, and broccoli) in BRPC patients,^692^ it was found the phytotherapetic intervention was well-tolerated, and the treatment group experienced a non-significant increase in the log-slope of PSADT (pre treatment and post-treatment was 10.2 and 5.5 months, respectively), while the placebo group experienced no change in the log-slope of PSADT (pre treatment and post-treatment was 10.8 and 10.9 months, respectively). It suggested this phytotherapeutic intervention is feasible in BRPC patients.

#### CPT

The toxicity of CPT greatly limits its application, current clinical research mostly focuses on evaluating the effectiveness of NLG207 (formerly CRLX101), novel cyclodextrin-containing polymer conjugate of CPT. A phase I/II trial of CRLX101 with capecitabine and radiotherapy as neoadjuvant treatment for locally advanced rectal cancer was conducted by Sanoff el al. The maximum tolerated dose and recommended phase 2 dose of weekly administration of CRLX101 was determined to be 15 mg/m^2^, and 6/31 (19%) of patients achieved overall pathologic CR, with an additional 18/31 (58%) patients experienced a moderate response. Therefore, the addition of CRLX101 to capecitabine-based CRT might be a feasible combination strategy with good tolerance for locally advanced rectal cancer patients.^693^ The team of Haas conducted a phase II trial of CRLX101 in combination with bevacizumab versus standard of care (approved targeted agents in the third-/fourth-line setting) in 111 patients with metastatic renal cell carcinoma.^694^ However, there was no significant difference in PFS between the two groups with CRLX101 plus bevacizumab group (3.7 months; 95% CI, 2.0–4.3) and standard of care group (3.9 months; 95% CI, 2.2–5.4). Schmidt et al. conducted another clinical trial of NLG207 with enzalutamide in treating advanced metastatic castration-resistant prostate cancer post-enzalutamide. In this study, a total of 4 patients were included, but only 2 patients were able to evaluate for toxicity, and 2 patients had a decrease in PSA levels compared to baseline. This study suggests that the combination of 12 mg/m^2^ NLG207 and 160 mg enzalutamide is an intolerable regimen in metastatic castration-resistant prostate cancer patients who have undergone multiple treatments, with the probable bladder toxicity caused by long-tern exposure to CPT in the bladder, even at a low concentration.^695^

#### Vinblastine

Methotrexate, vinblastine, doxorubicin, and cisplatin (MVAC) was considered as one of the most effective chemotherapy regimens for eurothelial cancer. Choueirii et al. explored the efficacy and safety of neoadjuvant dose-dense MVAC (dd-MVAC) with pegfilgrastim support in muscle-invasive urothelial cancer (MIUC).^696^ It was concluded this chemotherapy regimen was effective as 49% patients achieved pathologic response on cystectomy, and 1-year disease-free survival was 89% vs. 67% for patients who achieved pathologic response compared with those who did not. Moreover, dd-MVAC chemotherapy is also effective in nonmetastatic muscle-invasive bladder cancer (MIBC) patients. A randomized phase III controlled study comparing the efficacy of dd-MVAC or gemcitabine and cisplatin (GC) in MIBC patients was designed.^697,698^ The dd-MVAC arm was associated with a significantly longer time to tumor progression (TTP, 3-year rate: 69% vs. 58%,), and dd-MVAC also improved 3-years PFS over GC arm (66% vs. 56%), suggested dd-MVAC regimen is superior and it should be considered as a treatment option for MIBC patients in the future.

#### Homoharringtonine

The clinical trials of homoharringtonine is mainly focused on the treatment of leukemia. The HAG regimen with a low-dose homoharringtonine, cytarabine, and granulocyte colony-stimulating factor was evaluated by Liu et al. used as an induction therapy for untreated AML patients. The CR was 65.5% (19/29), with ORR of 86.1%.^699^ There were 9 cases of recurrence in CR patients, and the median disease-free survival of 19 CR patients were 12 months, with the median OS of 20 months.^699^ Homoharringtonine-based regimen is also beneficial to childhood AML. A multicenter, randomized clinical trial was performed with 1258 patients, the overall CR of the homoharringtonine-based (H arm) was remarkable higher than that in the etoposide-based (E arm) (79.9% vs. 73.9%), and according to the intention-to-treat analysis, the 3-year OS was 69.2%, 62.8% in H arm and E arm, respectively; the 3-year event-free survival was 61.1%, 53.4% in H arm and E arm, respectively.^700^ It is worth noting that in the subgroup analysis, patients with AML_1_-ETO positivity in the H + ATRA-based arm showed a better 3-year event-free survival compared with those in the E + cytarabine-based arm (73.6% vs. 52.8%).^700^ The homoharringtonine-based regimen may be a better choice for treating newly diagnosed children with AML, especially those with AML_1_-ETO. Refractory/relapsed acute myeloid leukemia (R/R AML) is a very challenging complication in AML treatment, with the prognosis dismal. The addition of homoharringtonine might bring new hope to R/R AML patients. A BHA regimen consisted with bortezomib, homoharringtonine, and cytarabine was evaluated for patients with R/R AML. After one course of BHA treatment, the ORR of 21 patients was 52.4%, with composite complete remission rate [CRc, CR plus CR with incomplete hematologic recover (CRi)] of 38.1%, and 9 cases harbored FLT3 mutations exhibited an astonishing higher ORR than that without FLT3 mutations (66.7% vs. 16.7%), indicated BHA regimen as a well-tolerated and effective chemotherapy for patients with R/R AML, especially for FLT3 mutations.^701^ Another drug combination of venetoclax, azacytidine, and homoharringtonine (VAH) was executed by Jin et al. to investigate the activity and tolerability. The CRc of 96 enrolled patients was 70.8%, with ORR of 78.1%, in the patients with CRc, measurable residual disease (MRD)-negative was achieved in 58.8%. The median follow-up time was 14.7 months, with the median OS was 22.1 months, as well as event-free survival was 14.3 months.^702^ In another study, sorafenib was added to VAH combination to be used as a salvage therapy for R/R AML patients, and the CRc was 76.5% (39/51), with ORR of 82.4% (42/51), the median follow-up of 17.7 months and the OS was 18.1 months.^703^ The therapy of VAH and combination therapy of sorafenib and VAH both achieved a high CRc and well-tolerance, suggest this regimen might be a suitable treatment option for R/R AML. Li et al. reported a phase II clinical trial for azacitidine with homoharringtonine, idarubicin, and cytarabine (HIA) in treating newly diagnosed AML patients.^704^ The results indicated that the combination therapy has high efficacy for young or elderly patients fit for intensive chemotherapy as first-line induction therapy with 95% (19/20) of patients gained ORR, 89.5% (17/19) had undetectable minimal residual disease. The median OS and RFS were not achieved during the follow-up period (median follow-up was 733 days), and the estimated 2-year OS and RFS were 87.5% and 87.1%, respectively, demonstrated this regimen was an effective and well-tolerance for previously untreated young or fit older patients with AML.^704^

Currently, compared to synthetic drugs/marketed drugs, there are relatively few studies on natural compounds-based cancer treatment/prevention with available data, and the administration and follow-up time is shorter. Due to the long time required to determine the benefits of cancer chemoprevention, longer administration and follow-up periods are necessary. Long-term intervention also increases the likelihood of research subjects being lost to follow-up, so it is recommended to have a large sample size as much as possible. The current effectiveness evaluation of cancer chemoprevention lacks clear biomarkers, although it can be assessed by detecting tumor markers or gene expression levels, these indicators may lack specificity and sensitivity for cancer prevention, making it difficult to assess the safety of long-term effects and determining the optimal dosage and duration of use of preventive agents. In addition, necessary imaging examinations will make the results more objective, and the advantages of the main results are significantly higher than the form of survey questionnaire. Moreover, as the long-term use of preventive agents may lead to the development of drug resistance and reduce the preventive effect, it is necessary to conduct in-depth research and evaluation on long-term safety.

## Research strategies for the discovery of low abundance and high anticancer activity natural compounds

**Fig. 6:**
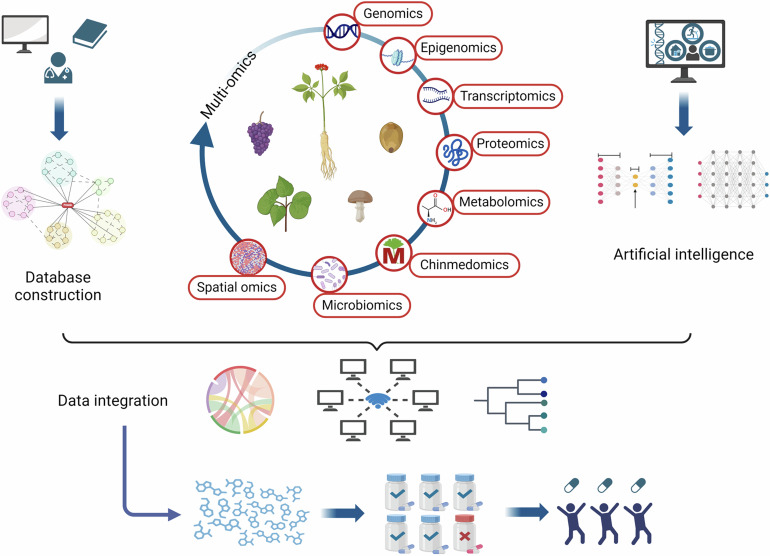
Research strategies for the discovery of low-abundance and high anticancer activity natural compound. Here we simply show through constructing the database via literature search and experience inheritance, omics technologies integration method based on molecular network construction at different levels of each omics, and AI (machine learning and deep learning)-driven assist the discovery of low-abundance and high anticancer activity natural compound. The ultimate goal of data integration is to discover low-abundance and high-activity anticancer components from complex natural product system and elucidate their biological significance. This figure was created with BioRender.com

The investigation on anticancer drugs has never stopped searching for better and less side effect agents. In addition to chemically synthesized anticancer compounds, natural compounds have been proven to be a highly promising alternative source for the discovery of anticancer drugs, and facilitate drug discovery by providing novel and unique compounds.^705–707^ Compared to typical synthetic small molecules, natural compounds are rich in biologically active compounds covering a wider chemical space.^705^ They serve as both a direct source of drugs and leading compounds.^708^ Currently, multitude of high abundance components have been repeatedly discovered, however, natural compounds with low abundance but high activity abound in nature, and they were considered to be important treasure trove for drug development. Due to the low abundance of these ingredients, they are difficult to be analyzed and detected or far below the detection limit of instruments, and are even insufficient for biological evaluation in multiple consecutive phenotypes or target-based assays.^709^ Nevertheless, they may exert a significant role in cancer prevention and treatment, and act as leading compounds for novel drug discovery. With the continuous innovation and cross fusion of modern analytical technology, bioinformatics, AI, multi-omics and other technologies, the discovery of low-abundance and high-activity anticancer ingredients has been greatly promoted (Fig. 6).^705,710^

### Natural compounds databases inspired leading compounds

The digital revolution is driving significant changes in a novel mean for storing, distributing, and using information. Massive amounts of data are organized, standardized, and stored as database, providing valuable resources for researchers. The establishment of database can better administrate, analyze, collect, and investigate information on chemical composition, structure, properties, biological activity, etc., and assist in various stages of drug discovery and development.^711^ There currently exists specialized natural compound databases, such as LOTUS,^712^ NPASS,^713^ Super Natural II,^714^ etc., which involved structural information, physical and chemical properties, and cross-references with other resources. Besides, herbal resource databases that include natural compounds and targets of action are helpful, such as TCMSP,^715^ TCMID,^716^ SymMap,^717^ BATMAN-TCM 2.0,^718^ etc. Universal compound databases of DrugBank, ChemSpider,^719^ PubChem,^720^ also presented abundant resources for the discovery of natural compounds. However, the current pattern of natural compounds-related databases is highly fragmented, repetitive, and difficult to integrate. It was reported over 120 different natural product databases were available including commercial and non-commercial resource.^721^ Therefore, in order to fully utilize the information in the database, it is necessary to coordinate and integrate the data in database, which is crucial for both users and developers. The cost of using commercial databases is also a barrier, although some companies can offer discounts to some researchers or institutions, it is expected to significantly reduce this barrier by creating high-quality FAIR-compliant (with the principles of findable, accessible, interoperable and reusable) resources.^722–724^ Increasingly scientists are using more precise analysis and extensive calculation methods to mine data from massive databases, accelerating the discovery of low-abundance and high anticancer activity natural compounds.

### Omics technologies assisted acceleration

Historically, bioactivity-guided separation was the main method to isolate and identify individual ingredients from complex natural compound mixtures.^725^ Due to the time-consuming, low-abundance components are prone to disappear during the separation process, currently, scientists have shifted away from bioactivity-guided separation to omics technologies to guide natural compounds discovery.^725,726^ Various high-throughput screening techniques, represented by genomics, transcriptomics, proteomics, metabolomics, chinmedomics, spatial multi-omics, etc., can efficiently and quickly reveal detailed biological information of diseases and drug efficacy, playing significant role in promoting human disease research.^727^ With the reduction of detection costs and the rapid development of high-throughput analytical instruments, the advantages of omics technologies in guiding natural compound discovery are gradually emerging.^726^ Each type of omics data provides differentially expressed disease-related molecules that can serve as biomarkers during disease progression and as indicators of changes that occur in the body under the influence of internal and external environments.^728–730^ Omics science focuses on the interactions between systematicity, integrity, and interactions within internal environment of living organisms, exploring the relationships between genes, environment, and phenotype, and thus assisting in the discovery of bioactive natural compounds.^731^ Omics technologies can assist in the discovery of natural compounds from the following aspects: 1) explore known structures with novel functions, 2) known functions with novel structures, 3) unknown structures with unknown functions.^732^ Li et al. employed network pharmacology to predict tricin is a bioactive component of Weijing formula, and discovered key differential metabolites sphingosine-1-phosphate and its upstream metabolic enzymes sphingosinase 1 and sphingosinase 2 by metabolomics, it was verified through further experiments that tricin mainly inhibits NSCLC tumor growth by suppressing PRKCA/SPHK/S1P signaling.^733^ Through screening the natural compound database, Hao et al. identified a diterpenoid compound named nodosin, might have potential anti-bladder cancer activity. Through the joint analysis of transcriptomics and proteomics, nodosin was found can prevent cell proliferation by inducing apoptosis, blocking the S phase cell cycle, destroying the cytoskeleton to inhibit migration, and significantly inhibit the tumor size of xenograft tumor mice.^734^ In addition, chinmedomics was considered as an effective strategy to discover bioactive natural compounds by integrating serum pharmacochemistry of traditional Chinese medicine, metabolomics, big data analysis, and bioinformatics.^735–741^

### AI-enabled exploration

In the era of big data, the volume and diversity of data are dramatically increasing. The global data volume has increased from ~1.5 zettabytes in 2009 to an estimated 175 zettabytes by 2025.^722,742^ With the emergence of new technologies and open data initiatives, computational methodologies have brought a paradigm shift in the field of drug discovery, providing indispensable tools for the entire process of drug development. These technologies greatly reduce costs and enhance the effectiveness of the process of identifying and producing new medications.^743^ The rapid development of AI with the wider accessibility of supercomputing resources, and graphics processing unit-accelerated computing made large-scale computer simulation become feasible.^744^ AI provides powerful technologies for extensive data analysis and identifying compounds with high therapeutic potential to accelerate the drug discovery process. Machine learning and deep learning are the mainly subsets of AI, and they are increasingly being applied to drug discovery pipelines, mainly because they can facilitate data-driven modeling processes to learn from large datasets.^745,746^ Through the use of algorithms, statistical models, and deep neural networks to study and execute virtual drug screening, predict chemical structures, drug-related parameters, activities, and targets of action, the accuracy of predicting anticancer potential of natural compounds has been greatly enhanced.^709,747,748^

Li et al. exploited a web server named CDRUG with a relative frequency-weighted fingerprint to describe the molecules, and a hybrid score was introduced to test molecular similarity.^749^ Subsequently, they screened the 21334 compounds from TCM Database@Taiwan, and a total of 5278 compounds were predicted exhibiting anticancer activity, with 75% of them were highly similar to approved anticancer medications, and 346 of them exerted high activity in 60 cell line tests.^750^ Rayan et al. employed ISE algorithm to construct a prediction model to detect natural compounds with anticancer activity with 2892 natural compounds as the test subjects, and 12 natural compounds (neoechinulin, colchicine, piperolactam, etc.) were disclosed as potential anticancer candidates.^751^ Furthermore, there are few researches reported on these screened natural compounds, and further research is needed to test their activity.^751^ Nguyen et al. used machine learning approach incorporated with evolutionary computer to construct a computing framework, termed iANP-EC, to identify natural compounds with anticancer activity. By screening of collected 997 compounds, it was proved that iANP-EC was a stable, robust, and effective framework with an AUC value of 0.9193.^752^ Ouyang et al. developed an AI-powered omics strategy for innovative drug discovery to take pyroptosis therapy for TNBC as a paradigm.^753^ By using large-scale TNBC queues and drug databases for data mining, a neural network for regulating biological factors was established, which can quickly screen and optimize compound with pyroptosis drugs, and the combination of glycyrrhetinic acid and mitoxantrone has been ultimately identified as candidate drug.^753^ Furthermore, a nanococrystal of glycyrrhetinic acid and mitoxantrone was formed to improve its solubility and exhibit stronger antitumor activity than the combination of abraxane and anti-PD-1 drugs. Despite the powerful capabilities of AI, its use for potential natural compounds drug discovery is still in the infant stage, and many issues still need to be addressed. For example, the challenges from data, such as the scale, growth, diversity, and uncertainty of data. With the increasing availability of high-quality and ideal labeled training data, more appropriate models will be created for the discovery of anticancer bioactive natural compounds, which will also lead to the discovery of more natural compounds, thereby improving the diversity of training data, further promote the development of the field. In addition, a well-maintained and accessible database is crucial for developing, testing, and establishing AI frameworks. It is also necessary to strengthen the close cooperation between computer and biology/pharmacy experts, so that the developed AI models can truly meet the needs of reality, and can be effectively validated and continuously adjusted to demonstrate strong performance in the real world and be widely used by researchers.

Based on natural compounds-related databases, omics techniques, AI, and bioinformatics technologies, they effectively associate natural compounds with targets and diseases. Especially the datasets generated by databases and multi-omics technologies are massive and complex, making them suitable for joint application with AI. The combination use can greatly improve data analysis, biological interpretation, and predictive statistical modeling.^754^ However, each stage of joint application brings unique challenges that require specialized methods and close collaboration among various researchers to overcome existing bottlenecks. Firstly, coordinating data scale is challenging as multidimensional data units vary depending on omics types and instruments.^755,756^ In addition, the significant differences in metabolite concentrations and the presence of isomers complicates the process of finding meaningful correlation with other omics data, and the influence of dietary and environmental factors increases this complexity.^757^ Therefore, choosing appropriate data integration method is crucial, meanwhile, the interpretation of multi-omics data results requires the employment of appropriate interpretation models to analyze important variables under disease background. Although AI can quickly adapt to large amounts of new data, it still needs to be monitored and updated to ensure the accuracy of analysis. Moreover, AI requires substantial computational resources and professional knowledge, which are not easily accessible in the existing medical environment. Although the rapid development of sequencing and spectroscopic technologies, as well as the availability of computational tools have stimulated research on natural compounds worldwide, there is currently limited research on the discovery of active ingredients from multi-omics aspect.

## Moving toward precision cancer chemoprevention

Although there are diversified strategies for cancer prevention currently, the preventive effect is not ideal. Due to individual differences in behavior, lifestyle, and environmental exposure, as well as the presence of tumor heterogeneity, gene mutations of different cancer cells in the same patient may vary. Moreover, in the process of cancer prevention and treatment, new mutations or fusion genes might occur, which may disrupt the targets of drugs and render chemopreventive agents ineffective.^758^ Patients also have different reactions to medications, resulting in poor preventive effects.^759^ Precision medicine is defined as tailoring clinical strategies based on genomic, genetic, behavioral, and environmental backgrounds of individual patients.^758,760–762^ With the development of molecular biology, high-throughput multi-omics, bioinformatics and AI, precision therapy is exerting an increasingly prominence role in oncology.^763–765^ Personalized cancer chemoprevention has the potential to improve prevention effectiveness, prolong patient survival time, and enhance their quality of life. Cancer chemoprevention is also undergoing revolutionary changes, shifting towards precision prevention, gradually improving effectiveness, and patient-centeredness. The United States Preventive Services Task Force (USPSTF) has incorporated precision prevention into evidence-based recommendations at the population level.^766–768^ The individuals age between 50-59 who have a life expectancy and risk of cardiovascular disease of at least 10 years, and are willing to take low-dose aspirin daily and are not at risk of bleeding, the USPSTF recommended to use low-dose aspirin to prevent cardiovascular disease and CRC.^769^ In addition, tamoxifen and raloxifene are proved to have significant efficacy in preventing ER-positive breast cancer.^770,771^ However, studies have shown that SERMs have potential hazards in prevention, which will increase the risk of uterine cancer among patients taking tamoxifen. In order to eliminate this hazard, a breast cancer prevention trial is investigating the efficacy of tamoxifen delivered through breast skin to achieve local prevention (NCT02993159).

In addition to individual differences in metabolic enzymes, individual characteristics that affect drug transporter action are important factors affecting natural compounds and other agent response. For example, BCRP is a key transport protein, which is encoded by ABCG2 gene and participates in the special function of patients in drug transport.^772^ There is a common genetic polymorphism in the ABCG2 gene worldwide, the common ABCG2 mutations can affect the efficacy of cancer drug therapy and the occurrence of adverse drug reactions, leading to changes in drug reactions and disease risk. Apigenin has good binding affinity with BCRP,^773^ compared with normal rats, the expression of BCRP/ABCG2 protein in the liver and intestine was significantly reduced after apigenin pretreatment, which can improve the systemic bioavailability and absorption rate of dasatinib.^774^ When compared to using doxorubicin alone, exposure to 2 μmol/L apigenin can increase the accumulation of doxorubicin by 29%.^775^ This indicates that the differences in ABCG2 genes may have impact on the chemoprevention of natural compounds. Cytokine gene polymorphism plays key role in immune regulation,^776,777^ for example, IL-10 is an important anti-inflammatory cytokine, and plays crucial role in maintaining intestinal homeostasis and balancing gut microbiota with the digestive system.^778^ The single nucleotide polymorphisms in IL genes may dramatically affect protein expression levels or alter their function, leading to gastritis or ulcer, and eventually promote cancer occurrence.^779^ McCann et al. indicated rs1800896 single nucleotide polymorphism in the promoter region of the IL-10 gene linked with the reduction of IL-10 and severity of IBD severity.^780^ Curcumin can attenuate the reduction of IL-10 mutations in a dose-dependent manner and alleviate the severity of IBD, thus, IBD patients with tested gene variants may benefit from increased intake of curcumin. Cancer genome sequencing revealed widespread genetic heterogeneity and profound genomic instability in advanced tumors,^781^ therefore, gene sequencing is necessary for precision medicine, as it can provide more information about gene mutations and is crucial for selecting more effective drugs and dosages.

The current progress in cancer chemoprevention is still limited, but generous work is still being carried out, researchers are actively exploring biomarkers for various types of cancer and translating them into clinical applications. The rapid development of spatial omics approaches further enable comprehensively understand the interactions between cells from a multidimensional perspective. In addition, the continuous development of patient-derived organoids and organoids co-culture model systems, the structure, unique cancer characteristics, and genetic features of tumors are faithfully reproduced, which can better guide personalized prevention. With the continuous in-depth research on preventive agents that can be used for cancer chemoprevention, such as natural compounds, and the publication of multiple related clinical trial results in the future, more cancer chemoprevention agents will assist achieve personalized prevention for individuals, providing unprecedented opportunities for people, and improving the current dilemma in oncology.

## Conclusion and future perspectives

**Fig. 7:**
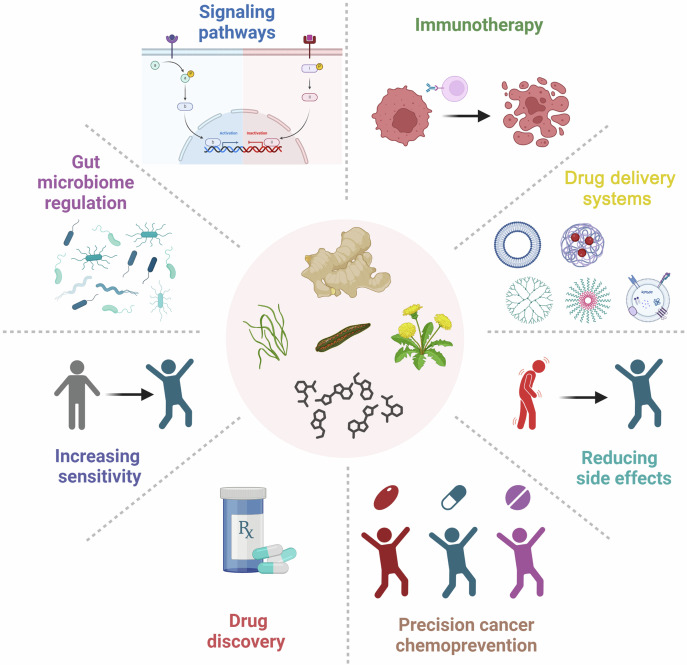
The roles of natural compounds in chemoprevention. Natural compounds have shown great promise in cancer chemoprevention by targeting on signaling pathways, immune checkpoints and gut microbiota, reducing adverse reactions, as well as improving chemotherapy drug sensitivity. In addition, drug delivery systems also enhance the bioavailability of natural compounds, making them more robust for cancer prevention. Natural compounds are not only treasure reservoir for drug discovery, but will also achieve precise cancer chemoprevention in the future to better serve human health. This figure was created with BioRender.com

Cancer chemoprevention represents a promising strategy for mitigating the incidence and mortality rates associated with tumor development. Discovering novel, safe, and effective chemopreventive agents is crucial for gaining more acceptance of cancer chemoprevention. The development and application of preventive agents have gradually shifted from drugs with a single target and stronger side effects to natural compounds with less toxicity regulated by multiple pathways and targets. The multi-target and multi-pathway properties of natural compounds enable them to effectively counteract the biological complexity of cancer, indicating natural compounds provide extensive and promising resources for cancer chemoprevention (Fig. 7). However, cancer prevention agents based on natural compounds still have a long way to go before clinical translation. Most chemopreventive agents are currently in the preclinical research stage, and the preventive effects of natural compounds on cancer can be easily discovered in cell lines or animal models, however, it is difficult to apply the results as translational evidence for direct entry into clinical trials. Considering the duration of clinical trials for discovering cancer chemopreventive drugs, selecting appropriate preclinical research models that fully simulate the process of human tumor occurrence and development may result in greater success of candidate compounds in clinical trials. Burgeoning technologies such as patient-derived organoids and organoid co-culture model systems can be combined. Moreover, most studies evaluate only the short-term effects of natural compounds, and the mid-term/long-term effects should also be examined. In addition, the appropriate dosage is also crucial for the success of clinical trials, because depending solely on dietary cancer chemoprevention in high-risk populations appears unreasonable.^782^ Therefore, conducting phase 0 and phase I clinical trials on potential natural compounds is necessary. Appropriate clinical endpoints (including the potential to utilize surrogate, early, or quality-of-life endpoints) appear to be equally important for the development of novel preventive agents, as evidenced by past experiences, such as the approval of drugs for AK treatment and the accelerated approval of celecoxib, it should be tailored to specific disease environments to make endpoints clinically meaningful.^15^

Natural compounds generally possess characteristics of low bioavailability, and the development of synthetic analogs with better pharmacokinetic properties using natural compounds as scaffold is an alternative method. For organs with unique characteristics, local administration can be attempted for cancer prevention during research, and the efficacy obtained may be more significant than that obtained by systemic administration, for example, inhalation administration can be attempted for lung cancer. In addition, the development of novel drug delivery systems and building new drug loading platforms to accurately control drug-targeted delivery to targets, along with limiting systemic toxicity, is also a development direction.

The definition of high-risk populations without obvious characteristics, the uncertainty of the time from precancerous lesions to invasive cancer, metastasis, and recurrence have hindered chemoprevention research. Screening reliable biomarkers is of great significance for promoting the field of cancer chemoprevention, which assisting in screening high-risk populations, evaluating the effectiveness of cancer prevention agents, and assessing prognosis. The risk and prediction models established with the help of AI and big data also provide assistance for preclinical and clinical research on cancer chemoprevention. The development of cancer chemoprevention agents involves several disciplines, and it was advocated for data sharing between institutions. Nonetheless, determining how to better protect patient privacy is also an important task.

To summarize, the development of novel cancer chemopreventive agents is an alternative method to alleviate the burden caused by cancer, and natural compounds have excellent broad prospect as cancer chemopreventive agents, it provides a window of opportunity for the discovery of cancer chemopreventive agents, regardless of numerous obstacles. Although a few approved cancer chemopreventive agents are available today, the discovery and validation results of cancer chemopreventive agents are not exciting, while, without taking a single step, one can travel thousands of miles. With the continuous emergence of various technologies and the collaborative efforts of personnel from various disciplines, we believe that cancer chemoprevention can inspire people greatly.

## References

[1] Bray, F. et al. Global cancer statistics 2022: GLOBOCAN estimates of incidence and mortality worldwide for 36 cancers in 185 countries. *CA Cancer J. Clin.* **74**, 229–263 (2024). 38572751 10.3322/caac.21834

[2] Ginsburg, O. et al. The role of genomics in global cancer prevention. *Nat. Rev. Clin. Oncol.* **18**, 116–128 (2021). 32973296 10.1038/s41571-020-0428-5

[3] Gotay, C., Dummer, T. & Spinelli, J. Cancer risk: prevention is crucial. *Science* **347**, 728 (2015). 25656659 10.1126/science.aaa6462

[4] Page, I. H. Low cholesterol–low fat diets in prevention and treatment of atherosclerosis. *Med. Clin. North Am.* **36**, 195–199 (1952). 14909804

[5] Serrano, D., Lazzeroni, M. & Bonanni, B. Cancer chemoprevention: much has been done, but there is still much to do. State of the art and possible new approaches. *Mol. Oncol.* **9**, 1008–1017 (2015). 25556583 10.1016/j.molonc.2014.12.006 PMC5528739

[6] Meyskens, F. L., Jr. et al. Cancer Prevention: obstacles, challenges and the road ahead. *J. Natl. Cancer Inst.* **108**, djv309 (2016). 10.1093/jnci/djv309 PMC4907357 26547931

[7] Penny, L. K. & Wallace, H. M. The challenges for cancer chemoprevention. *Chem. Soc. Rev.* **44**, 8836–8847 (2015). 26595684 10.1039/c5cs00705d

[8] Menter, D. G. & Bresalier, R. S. An Aspirin a day: new pharmacological developments and cancer chemoprevention. *Annu. Rev. Pharm. Toxicol.* **63**, 165–186 (2023). 10.1146/annurev-pharmtox-052020-023107 36202092

[9] Patra, S. et al. Chemotherapeutic efficacy of curcumin and resveratrol against cancer: chemoprevention, chemoprotection, drug synergism and clinical pharmacokinetics. *Semin. Cancer Biol.* **73**, 310–320 (2021). 33152486 10.1016/j.semcancer.2020.10.010

[10] Cai, H. et al. Cancer chemoprevention: evidence of a nonlinear dose response for the protective effects of resveratrol in humans and mice. *Sci. Transl. Med.* **7**, 298ra117 (2015). 26223300 10.1126/scitranslmed.aaa7619 PMC4827609

[11] William, W. N. Jr., Heymach, J. V., Kim, E. S. & Lippman, S. M. Molecular targets for cancer chemoprevention. *Nat. Rev. Drug Discov.* **8**, 213–225 (2009). 19247304 10.1038/nrd2663

[12] Baena Ruiz, R. & Salinas Hernandez, P. Cancer chemoprevention by dietary phytochemicals: epidemiological evidence. *Maturitas* **94**, 13–19 (2016). 27823732 10.1016/j.maturitas.2016.08.004

[13] Dunn, B. K. & Kramer, B. S. Cancer prevention: lessons learned and future directions. *Trends Cancer* **2**, 713–722 (2016). 28138568 10.1016/j.trecan.2016.11.003 PMC5271581

[14] Maresso, K. C. et al. Molecular cancer prevention: current status and future directions. *CA Cancer J. Clin.* **65**, 345–383 (2015). 26284997 10.3322/caac.21287 PMC4820069

[15] Reynolds, A. R. et al. A view on drug development for cancer prevention. *Cancer Discov.* **13**, 1058–1083 (2023). 37067191 10.1158/2159-8290.CD-22-0776

[16] Newman, D. J. & Cragg, G. M. Natural products as sources of new drugs over the nearly four decades from 01/1981 to 09/2019. *J. Nat. Prod.* **83**, 770–803 (2020). 32162523 10.1021/acs.jnatprod.9b01285

[17] Surh, Y. J. Cancer chemoprevention with dietary phytochemicals. *Nat. Rev. Cancer* **3**, 768–780 (2003). 14570043 10.1038/nrc1189

[18] Amin, A. R., Kucuk, O., Khuri, F. R. & Shin, D. M. Perspectives for cancer prevention with natural compounds. *J. Clin. Oncol.* **27**, 2712–2725 (2009). 19414669 10.1200/JCO.2008.20.6235 PMC2690394

[19] Katz, L. & Baltz, R. H. Natural product discovery: past, present, and future. *J. Ind. Microbiol. Biotechnol.* **43**, 155–176 (2016). 26739136 10.1007/s10295-015-1723-5

[20] G, M. S. et al. Cancer chemoprevention: a strategic approach using phytochemicals. *Front. Pharm.* **12**, 809308 (2021). 10.3389/fphar.2021.809308 PMC8793885 35095521

[21] Shu, L. et al. Phytochemicals: cancer chemoprevention and suppression of tumor onset and metastasis. *Cancer Metastasis Rev.* **29**, 483–502 (2010). 20798979 10.1007/s10555-010-9239-y

[22] Russo, G. L., Spagnuolo, C. & Russo, M. Reassessing the role of phytochemicals in cancer chemoprevention. *Biochem. Pharmacol.* **228**, 116165 (2024). 38527559 10.1016/j.bcp.2024.116165

[23] Mize, B. K. et al. Discovery and development of botanical natural products and their analogues as therapeutics for ovarian cancer. *Nat. Prod. Rep.* **40**, 1250–1270 (2023). 37387219 10.1039/d2np00091a PMC10448539

[24] Mignani, S. et al. Dendrimers in combination with natural products and analogues as anti-cancer agents. *Chem. Soc. Rev.* **47**, 514–532 (2018). 29154385 10.1039/c7cs00550d

[25] Fisher, B. et al. Tamoxifen for prevention of breast cancer: report of the National Surgical Adjuvant Breast and Bowel Project P-1 Study. *J. Natl. Cancer Inst.* **90**, 1371–1388 (1998). 9747868 10.1093/jnci/90.18.1371

[26] Cuzick, J. et al. Overview of the main outcomes in breast-cancer prevention trials. *Lancet* **361**, 296–300 (2003). 12559863 10.1016/S0140-6736(03)12342-2

[27] Cuzick, J. et al. Long-term results of tamoxifen prophylaxis for breast cancer–96-month follow-up of the randomized IBIS-I trial. *J. Natl. Cancer Inst.* **99**, 272–282 (2007). 17312304 10.1093/jnci/djk049

[28] Vogel, V. G. et al. Update of the National Surgical Adjuvant Breast and Bowel Project Study of Tamoxifen and Raloxifene (STAR) P-2 Trial: preventing breast cancer. *Cancer Prev. Res.* **3**, 696–706 (2010). 10.1158/1940-6207.CAPR-10-0076 PMC2935331 20404000

[29] Moen, M. D. & Keating, G. M. Raloxifene: a review of its use in the prevention of invasive breast cancer. *Drugs* **68**, 2059–2083 (2008). 18778124 10.2165/00003495-200868140-00008

[30] Goss, P. E. et al. Exemestane for breast-cancer prevention in postmenopausal women. *New Engl. J. Med.* **364**, 2381–2391 (2011). 21639806 10.1056/NEJMoa1103507

[31] Cuzick, J. et al. Use of anastrozole for breast cancer prevention (IBIS-II): long-term results of a randomised controlled trial. *Lancet* **395**, 117–122 (2020). 31839281 10.1016/S0140-6736(19)32955-1 PMC6961114

[32] Patterson, S. L., Colbert Maresso, K. & Hawk, E. Cancer chemoprevention: successes and failures. *Clin. Chem.* **59**, 94–101 (2013). 23150056 10.1373/clinchem.2012.185389

[33] Lebwohl, M. et al. Long-term follow-up study of ingenol mebutate gel for the treatment of actinic keratoses. *JAMA Dermatol.* **149**, 666–670 (2013). 23553119 10.1001/jamadermatol.2013.2766

[34] Martin, G. & Swanson, N. Clinical findings using ingenol mebutate gel to treat actinic keratoses. *J. Am. Acad. Dermatol.* **68**, S39–S48 (2013). 23228305 10.1016/j.jaad.2012.09.050

[35] Pinsky, C. M. et al. Intravesical administration of bacillus Calmette-Guerin in patients with recurrent superficial carcinoma of the urinary bladder: report of a prospective, randomized trial. *Cancer Treat. Rep.* **69**, 47–53 (1985). 3881177

[36] Morales, A., Eidinger, D. & Bruce, A. W. Intracavitary Bacillus Calmette-Guerin in the treatment of superficial bladder tumors. *J. Urol.* **116**, 180–183 (1976). 820877 10.1016/s0022-5347(17)58737-6

[37] Lamm, D. L. et al. Bacillus Calmette-Guerin immunotherapy of superficial bladder cancer. *J. Urol.* **124**, 38–40 (1980). 6997513 10.1016/s0022-5347(17)55282-9

[38] Steinberg, G. et al. Efficacy and safety of valrubicin for the treatment of Bacillus Calmette-Guerin refractory carcinoma in situ of the bladder. The Valrubicin Study Group. *J. Urol.* **163**, 761–767 (2000). 10687972

[39] Cookson, M. S. et al. Use of intravesical valrubicin in clinical practice for treatment of nonmuscle-invasive bladder cancer, including carcinoma in situ of the bladder. *Ther. Adv. Urol.* **6**, 181–191 (2014). 25276228 10.1177/1756287214541798 PMC4144261

[40] Balar, A. V. et al. Pembrolizumab monotherapy for the treatment of high-risk non-muscle-invasive bladder cancer unresponsive to BCG (KEYNOTE-057): an open-label, single-arm, multicentre, phase 2 study. *Lancet Oncol.* **22**, 919–930 (2021). 34051177 10.1016/S1470-2045(21)00147-9

[41] Overholt, B. F. et al. Photodynamic therapy with porfimer sodium for ablation of high-grade dysplasia in Barrett’s esophagus: international, partially blinded, randomized phase III trial. *Gastrointest. Endosc.* **62**, 488–498 (2005). 16185958 10.1016/j.gie.2005.06.047

[42] Malagon, T., Franco, E. L., Tejada, R. & Vaccarella, S. Epidemiology of HPV-associated cancers past, present and future: towards prevention and elimination. *Nat. Rev. Clin. Oncol.* **21**, 522–538 (2024). 38760499 10.1038/s41571-024-00904-z

[43] Schiffman, M. et al. Carcinogenic human papillomavirus infection. *Nat. Rev. Dis. Prim.* **2**, 16086 (2016). 27905473 10.1038/nrdp.2016.86

[44] de Martel, C., Plummer, M., Vignat, J. & Franceschi, S. Worldwide burden of cancer attributable to HPV by site, country and HPV type. *Int. J. Cancer* **141**, 664–670 (2017). 28369882 10.1002/ijc.30716 PMC5520228

[45] Li, N. et al. Human papillomavirus type distribution in 30,848 invasive cervical cancers worldwide: Variation by geographical region, histological type and year of publication. *Int J. Cancer* **128**, 927–935 (2011). 20473886 10.1002/ijc.25396

[46] Finn, O. J. The dawn of vaccines for cancer prevention. *Nat. Rev. Immunol.* **18**, 183–194 (2018). 29279613 10.1038/nri.2017.140

[47] de Sanjose, S. & Delany-Moretlwe, S. HPV vaccines can be the hallmark of cancer prevention. *Lancet* **394**, 450–451 (2019). 31255298 10.1016/S0140-6736(19)30549-5

[48] Organization, W. H. Human papillomavirus (HPV) vaccination coverage. https://immunizationdata.who.int/global/wiise-detail-page/human-papillomavirus-(hpv)-vaccination-coverage (2024).

[49] Organization, W. H. Global strategy to accelerate the elimination of cervical cancer as a public health problem. https://www.who.int/publications/i/item/9789240014107 (2020).

[50] Rumgay, H. et al. Global, regional and national burden of primary liver cancer by subtype. *Eur. J. Cancer* **161**, 108–118 (2022). 34942552 10.1016/j.ejca.2021.11.023

[51] de Martel, C. et al. Global burden of cancer attributable to infections in 2018: a worldwide incidence analysis. *Lancet Glob. Health* **8**, e180–e190 (2020). 31862245 10.1016/S2214-109X(19)30488-7

[52] Chang, M. H. et al. Long-term effects of hepatitis B immunization of infants in preventing liver cancer. *Gastroenterology* **151**, 472–480.e471 (2016). 27269245 10.1053/j.gastro.2016.05.048

[53] Harpaz, R. et al. Elimination of new chronic hepatitis B virus infections: results of the Alaska immunization program. *J. Infect. Dis.* **181**, 413–418 (2000). 10669320 10.1086/315259

[54] Chien, Y. C. et al. Incomplete hepatitis B immunization, maternal carrier status, and increased risk of liver diseases: a 20-year cohort study of 3.8 million vaccinees. *Hepatology* **60**, 125–132 (2014). 24497203 10.1002/hep.27048

[55] Thompson, I. M. et al. The influence of finasteride on the development of prostate cancer. *New Engl. J. Med.* **349**, 215–224 (2003). 12824459 10.1056/NEJMoa030660

[56] Scardino, P. T. The prevention of prostate cancer–the dilemma continues. *New Engl. J. Med.* **349**, 297–299 (2003). 12824458 10.1056/NEJMe038109

[57] Logothetis, C. J. & Schellhammer, P. F. High-grade prostate cancer and the prostate cancer prevention trial. *Cancer Prev. Res.* **1**, 151–152 (2008). 10.1158/1940-6207.CAPR-08-0085 19138948

[58] Lotan, Y. et al. Implications of the prostate cancer prevention trial: a decision analysis model of survival outcomes. *J. Clin. Oncol.* **23**, 1911–1920 (2005). 15774783 10.1200/JCO.2005.03.137

[59] Lippman, S. M. & Lee, J. J. Reducing the “risk” of chemoprevention: defining and targeting high risk–2005 AACR Cancer Research and Prevention Foundation Award Lecture. *Cancer Res.* **66**, 2893–2903 (2006). 16540634 10.1158/0008-5472.CAN-05-4573

[60] Thompson, I. M. et al. Finasteride decreases the risk of prostatic intraepithelial neoplasia. *J. Urol.* **178**, 107–109 (2007). 17499284 10.1016/j.juro.2007.03.012

[61] Goodman, P. J. et al. Long-term effects of finasteride on prostate cancer mortality. *New Engl. J. Med.* **380**, 393–394 (2019). 30673548 10.1056/NEJMc1809961

[62] Sandler, R. S. et al. A randomized trial of aspirin to prevent colorectal adenomas in patients with previous colorectal cancer. *New Engl. J. Med.* **348**, 883–890 (2003). 12621132 10.1056/NEJMoa021633

[63] Logan, R. F. et al. Aspirin and folic acid for the prevention of recurrent colorectal adenomas. *Gastroenterology* **134**, 29–38 (2008). 18022173 10.1053/j.gastro.2007.10.014

[64] Rothwell, P. M. et al. Effect of daily aspirin on risk of cancer metastasis: a study of incident cancers during randomised controlled trials. *Lancet* **379**, 1591–1601 (2012). 22440947 10.1016/S0140-6736(12)60209-8

[65] Liang, P. S., Shaukat, A. & Crockett, S. D. AGA clinical practice update on chemoprevention for colorectal neoplasia: expert review. *Clin. Gastroenterol. Hepatol.* **19**, 1327–1336 (2021). 33581359 10.1016/j.cgh.2021.02.014

[66] Guirguis-Blake, J. M. et al. Aspirin use to prevent cardiovascular disease and colorectal cancer: updated evidence report and systematic review for the US Preventive Services Task Force. *JAMA***327**, 1585–1597 (2022).35471507 10.1001/jama.2022.3337

[67] Steinbach, G. et al. The effect of celecoxib, a cyclooxygenase-2 inhibitor, in familial adenomatous polyposis. *New Engl. J. Med.* **342**, 1946–1952 (2000). 10874062 10.1056/NEJM200006293422603

[68] Arber, N. et al. Celecoxib for the prevention of colorectal adenomatous polyps. *New Engl. J. Med.* **355**, 885–895 (2006). 16943401 10.1056/NEJMoa061652

[69] Giardiello, F. M. et al. Primary chemoprevention of familial adenomatous polyposis with sulindac. *New Engl. J. Med.* **346**, 1054–1059 (2002). 11932472 10.1056/NEJMoa012015 PMC2225537

[70] Meyskens, F. L. Jr. et al. Difluoromethylornithine plus sulindac for the prevention of sporadic colorectal adenomas: a randomized placebo-controlled, double-blind trial. *Cancer Prev. Res.* **1**, 32–38 (2008). 10.1158/1940-6207.CAPR-08-0042 PMC2562024 18841250

[71] Heer, E. et al. The efficacy of chemopreventive agents on the incidence of colorectal adenomas: a systematic review and network meta-analysis. *Prev. Med.* **162**, 107169 (2022). 35878711 10.1016/j.ypmed.2022.107169

[72] Lega, I. C. & Lipscombe L. L. Review: diabetes, obesity, and cancer-pathophysiology and clinical implications. *Endocr. Rev.* **41**, 33–52 (2020). 10.1210/endrev/bnz014 31722374

[73] Gandini, S. et al. Metformin and cancer risk and mortality: a systematic review and meta-analysis taking into account biases and confounders. *Cancer Prev. Res.* **7**, 867–885 (2014). 10.1158/1940-6207.CAPR-13-0424 PMC4154969 24985407

[74] Higurashi, T. et al. Metformin for chemoprevention of metachronous colorectal adenoma or polyps in post-polypectomy patients without diabetes: a multicentre double-blind, placebo-controlled, randomised phase 3 trial. *Lancet Oncol.* **17**, 475–483 (2016). 26947328 10.1016/S1470-2045(15)00565-3

[75] Liu, C. et al. New insights into the therapeutic potentials of statins in cancer. *Front. Pharm.* **14**, 1188926 (2023). 10.3389/fphar.2023.1188926 PMC10359995 37484027

[76] Mei, Z. et al. Effects of statins on cancer mortality and progression: a systematic review and meta-analysis of 95 cohorts including 1,111,407 individuals. *Int. J. Cancer* **140**, 1068–1081 (2017). 27859151 10.1002/ijc.30526

[77] Islam, M. M. et al. Statin use and the risk of hepatocellular carcinoma: a meta-analysis of observational studies. *Cancers* **12**, 671 (2020). 10.3390/cancers12030671 PMC7139959 32183029

[78] Facciorusso, A. et al. Statin use decreases the incidence of hepatocellular carcinoma: an updated meta-analysis. *Cancers* **12**, 874 (2020). 10.3390/cancers12040874 PMC7225931 32260179

[79] Zhang, X. et al. Association between statins types with incidence of liver cancer: an updated meta-analysis. *Curr. Med. Chem.* **31**, 762–775 (2024). 37393552 10.2174/0929867330666230701000400 PMC10661961

[80] Lin, J. R. et al. Regular use of aspirin and statins reduces the risk of cancer in individuals with systemic inflammatory diseases. *Cancer Res.* **84**, 1889–1897 (2024). 38536116 10.1158/0008-5472.CAN-23-2941

[81] Klein, E. A. et al. Vitamin E and the risk of prostate cancer: the Selenium and Vitamin E Cancer Prevention Trial (SELECT). *JAMA* **306**, 1549–1556 (2011). 21990298 10.1001/jama.2011.1437 PMC4169010

[82] Bryan, R. T. et al. Selenium and vitamin E for prevention of non-muscle-invasive bladder cancer recurrence and progression: a randomized clinical trial. *JAMA Netw. Open* **6**, e2337494 (2023). 37847504 10.1001/jamanetworkopen.2023.37494 PMC10582794

[83] Saavedra, D. & Crombet, T. CIMAvax-EGF: a new therapeutic vaccine for advanced non-small cell lung cancer patients. *Front. Immunol.* **8**, 269 (2017). 28348561 10.3389/fimmu.2017.00269 PMC5346887

[84] Flores Vega, Y. I. et al. Survival of NSCLC patients treated with cimavax-EGF as switch maintenance in the real-world scenario. *J. Cancer* **14**, 874–879 (2023). 37056397 10.7150/jca.67189 PMC10088885

[85] Rojas, L. A. et al. Personalized RNA neoantigen vaccines stimulate T cells in pancreatic cancer. *Nature* **618**, 144–150 (2023). 37165196 10.1038/s41586-023-06063-y PMC10171177

[86] Gainor, J. F. et al. T cell responses to individualized neoantigen therapy mRNA-4157 (V940) alone or in combination with pembrolizumab in the phase 1 KEYNOTE-603 study. *Cancer Discov.* **14**, 2209–2223 (2024). 10.1158/2159-8290.CD-24-0158 39115419

[87] Weber, J. S. et al. Individualised neoantigen therapy mRNA-4157 (V940) plus pembrolizumab versus pembrolizumab monotherapy in resected melanoma (KEYNOTE-942): a randomised, phase 2b study. *Lancet* **403**, 632–644 (2024). 38246194 10.1016/S0140-6736(23)02268-7

[88] Yarchoan, M. et al. Personalized neoantigen vaccine and pembrolizumab in advanced hepatocellular carcinoma: a phase 1/2 trial. *Nat. Med.* **30**, 1044–1053 (2024). 38584166 10.1038/s41591-024-02894-y PMC11031401

[89] Finn, R. S. et al. Pembrolizumab as second-line therapy in patients with advanced hepatocellular carcinoma in KEYNOTE-240: a randomized, double-blind, phase III trial. *J. Clin. Oncol.* **38**, 193–202 (2020). 31790344 10.1200/JCO.19.01307

[90] Gupta, B. et al. Plant lectins and their usage in preparing targeted nanovaccines for cancer immunotherapy. *Semin. Cancer Biol.* **80**, 87–106 (2022). 32068087 10.1016/j.semcancer.2020.02.005

[91] Yang, Y. & Lundqvist, A. Immunomodulatory effects of IL-2 and IL-15; implications for cancer immunotherapy. *Cancers* **12**, 3586 (2020). 10.3390/cancers12123586 PMC7761238 33266177

[92] Fishman, M. et al. Overall survival by clinical risk category for high dose interleukin-2 (HD IL-2) treated patients with metastatic renal cell cancer (mRCC): data from the PROCLAIM(SM) registry. *J. Immunother. Cancer* **7**, 84 (2019). 30917871 10.1186/s40425-019-0567-3 PMC6437874

[93] Brivio, F. et al. Pre-operative IL-2 immunoprophylaxis of cancer recurrence: long-term clinical results of a phase II study in radically operable colorectal cancer. *Oncol. Rep.* **6**, 1205–1207 (1999). 10523681 10.3892/or.6.6.1205

[94] Berraondo, P. et al. Cytokines in clinical cancer immunotherapy. *Br. J. Cancer* **120**, 6–15 (2019). 30413827 10.1038/s41416-018-0328-y PMC6325155

[95] Hudis, C. A. Trastuzumab–mechanism of action and use in clinical practice. *New Engl. J. Med.* **357**, 39–51 (2007). 17611206 10.1056/NEJMra043186

[96] Cameron, D. et al. 11 years’ follow-up of trastuzumab after adjuvant chemotherapy in HER2-positive early breast cancer: final analysis of the HERceptin Adjuvant (HERA) trial. *Lancet* **389**, 1195–1205 (2017). 28215665 10.1016/S0140-6736(16)32616-2 PMC5465633

[97] Perez, E. A. et al. Four-year follow-up of trastuzumab plus adjuvant chemotherapy for operable human epidermal growth factor receptor 2-positive breast cancer: joint analysis of data from NCCTG N9831 and NSABP B-31. *J. Clin. Oncol.* **29**, 3366–3373 (2011). 21768458 10.1200/JCO.2011.35.0868 PMC3164242

[98] Romond, E. H. et al. Trastuzumab plus adjuvant chemotherapy for operable HER2-positive breast cancer. *New Engl. J. Med.* **353**, 1673–1684 (2005). 16236738 10.1056/NEJMoa052122

[99] Maximiano, S., Magalhaes, P., Guerreiro, M. P. & Morgado, M. Trastuzumab in the treatment of breast cancer. *BioDrugs* **30**, 75–86 (2016). 26892619 10.1007/s40259-016-0162-9

[100] George, B. P., Chandran, R. & Abrahamse, H. Role of phytochemicals in cancer chemoprevention: insights. *Antioxidants* **10**, 1455 (2021). 10.3390/antiox10091455 PMC8466984 34573087

[101] Yarla, N. S. et al. Targeting arachidonic acid pathway by natural products for cancer prevention and therapy. *Semin. Cancer Biol.* **40-41**, 48–81 (2016). 26853158 10.1016/j.semcancer.2016.02.001

[102] Bishayee, A. & Sethi, G. Bioactive natural products in cancer prevention and therapy: progress and promise. *Semin. Cancer Biol.* **40-41**, 1–3 (2016). 27565447 10.1016/j.semcancer.2016.08.006

[103] Li, S. et al. Polyphenols as potential metabolism mechanisms regulators in liver protection and liver cancer prevention. *Cell Prolif.* **56**, e13346 (2023). 36229407 10.1111/cpr.13346 PMC9816945

[104] Patra, S. et al. Dietary polyphenols in chemoprevention and synergistic effect in cancer: clinical evidences and molecular mechanisms of action. *Phytomedicine* **90**, 153554 (2021). 34371479 10.1016/j.phymed.2021.153554

[105] Khan, H. et al. Targeting NF-kappaB signaling pathway in cancer by dietary polyphenols. *Crit. Rev. Food Sci. Nutr.* **60**, 2790–2800 (2020). 31512490 10.1080/10408398.2019.1661827

[106] Yan, L. & Spitznagel, E. L. Soy consumption and prostate cancer risk in men: a revisit of a meta-analysis. *Am. J. Clin. Nutr.* **89**, 1155–1163 (2009). 19211820 10.3945/ajcn.2008.27029

[107] Hwang, Y. W. et al. Soy food consumption and risk of prostate cancer: a meta-analysis of observational studies. *Nutr. Cancer* **61**, 598–606 (2009). 19838933 10.1080/01635580902825639

[108] Dong, J. Y. & Qin, L. Q. Soy isoflavones consumption and risk of breast cancer incidence or recurrence: a meta-analysis of prospective studies. *Breast Cancer Res. Treat.* **125**, 315–323 (2011). 21113655 10.1007/s10549-010-1270-8

[109] Wu, A. H., Yu, M. C., Tseng, C. C. & Pike, M. C. Epidemiology of soy exposures and breast cancer risk. *Br. J. Cancer* **98**, 9–14 (2008). 18182974 10.1038/sj.bjc.6604145 PMC2359677

[110] Chen, M. et al. Association between soy isoflavone intake and breast cancer risk for pre- and post-menopausal women: a meta-analysis of epidemiological studies. *PLoS ONE* **9**, e89288 (2014). 24586662 10.1371/journal.pone.0089288 PMC3930722

[111] Horia, E. & Watkins, B. A. Complementary actions of docosahexaenoic acid and genistein on COX-2, PGE2 and invasiveness in MDA-MB-231 breast cancer cells. *Carcinogenesis* **28**, 809–815 (2007). 17052999 10.1093/carcin/bgl183

[112] Chen, J. et al. Genistein induces apoptosis by the inactivation of the IGF-1R/p-Akt signaling pathway in MCF-7 human breast cancer cells. *Food Funct.* **6**, 995–1000 (2015). 25675448 10.1039/c4fo01141d

[113] Sergeev, I. N. Genistein induces Ca2+ -mediated, calpain/caspase-12-dependent apoptosis in breast cancer cells. *Biochem. Biophys. Res. Commun.* **321**, 462–467 (2004). 15358198 10.1016/j.bbrc.2004.06.173

[114] Liang, Y. S. et al. Genistein and daidzein induce apoptosis of colon cancer cells by inhibiting the accumulation of lipid droplets. *Food Nutr. Res.* **62**, 1384 (2018). 10.29219/fnr.v62.1384 PMC5965345 29849534

[115] Hsiao, Y. C. et al. Genistein induces apoptosis in vitro and has antitumor activity against human leukemia HL-60 cancer cell xenograft growth in vivo. *Environ. Toxicol.* **34**, 443–456 (2019). 30618158 10.1002/tox.22698

[116] Kaushik, S. et al. Genistein potentiates Centchroman induced antineoplasticity in breast cancer via PI3K/Akt deactivation and ROS dependent induction of apoptosis. *Life Sci.* **239**, 117073 (2019). 31751581 10.1016/j.lfs.2019.117073

[117] Tang, Q. et al. Genistein and AG1024 synergistically increase the radiosensitivity of prostate cancer cells. *Oncol. Rep.* **40**, 579–588 (2018). 29901146 10.3892/or.2018.6468 PMC6072286

[118] Hu, X. et al. Genistein-induced DNA damage is repaired by nonhomologous end joining and homologous recombination in TK6 cells. *J. Cell Physiol.* **234**, 2683–2692 (2019). 30070703 10.1002/jcp.27082

[119] Bilir, B. et al. Effects of genistein supplementation on genome-wide DNA methylation and gene expression in patients with localized prostate cancer. *Int. J. Oncol.* **51**, 223–234 (2017). 28560383 10.3892/ijo.2017.4017 PMC5467777

[120] Liu, X. et al. Genistein mediates the selective radiosensitizing effect in NSCLC A549 cells via inhibiting methylation of the keap1 gene promoter region. *Oncotarget* **7**, 27267–27279 (2016). 27029077 10.18632/oncotarget.8403 PMC5053648

[121] Romagnolo, D. F. et al. Genistein prevents BRCA1 CpG methylation and proliferation in human breast cancer cells with activated aromatic hydrocarbon receptor. *Curr. Dev. Nutr.* **1**, e000562 (2017). 29955703 10.3945/cdn.117.000562 PMC5998349

[122] Hirata, H. et al. Editorial expression of concern: genistein downregulates onco-miR-1260b and upregulates sFRP1 and Smad4 via demethylation and histone modification in prostate cancer cells. *Br. J. Cancer* **129**, 735 (2023). 37507546 10.1038/s41416-023-02365-0 PMC10421853

[123] Qin, H. et al. miR-363-3p/PTEN is involved in the regulation of lipid metabolism by genistein in HepG2 cells via ERbeta. *Phytomedicine* **115**, 154839 (2023). 37121060 10.1016/j.phymed.2023.154839

[124] Hirata, H. et al. Genistein downregulates onco-miR-1260b and upregulates sFRP1 and Smad4 via demethylation and histone modification in prostate cancer cells. *Br. J. Cancer* **110**, 1645–1654 (2014). 24504368 10.1038/bjc.2014.48 PMC3960620

[125] Yang, X. et al. Genistein restricts the epithelial mesenchymal transformation (EMT) and stemness of hepatocellular carcinoma via upregulating miR-1275 to inhibit the EIF5A2/PI3K/Akt pathway. *Biology* **11**, 1383 (2022). 10.3390/biology11101383 PMC9598820 36290289

[126] Yu, Y. et al. Soy isoflavone genistein inhibits hsa_circ_0031250/miR-873-5p/FOXM1 axis to suppress non-small-cell lung cancer progression. *IUBMB Life* **73**, 92–107 (2021). 33159503 10.1002/iub.2404

[127] Nechuta, S. J. et al. Soy food intake after diagnosis of breast cancer and survival: an in-depth analysis of combined evidence from cohort studies of US and Chinese women. *Am. J. Clin. Nutr.* **96**, 123–132 (2012). 22648714 10.3945/ajcn.112.035972 PMC3374736

[128] Konstantinou, E. K. et al. Molecular pathways of genistein activity in breast cancer cells. *Int. J. Mol. Sci.* **25**, 5556 (2024). 10.3390/ijms25105556 PMC11122029 38791595

[129] Pons, D. G. et al. The phytoestrogen genistein affects inflammatory-related genes expression depending on the ERalpha/ERbeta ratio in breast cancer cells. *Int. J. Food Sci. Nutr.* **70**, 941–949 (2019). 30945577 10.1080/09637486.2019.1597025

[130] Boretti, A. Quercetin as a cancer chemopreventive or chemotherapeutic agent: where we stand. *Phytother. Res.* **37**, 1227–1231 (2023). 36444390 10.1002/ptr.7699

[131] Zhou, B. et al. Quercetin inhibits DNA damage responses to induce apoptosis via SIRT5/PI3K/AKT pathway in non-small cell lung cancer. *Biomed. Pharmacother.* **165**, 115071 (2023). 37390710 10.1016/j.biopha.2023.115071

[132] Ding, L. et al. Quercetin induces ferroptosis in gastric cancer cells by targeting SLC1A5 and regulating the p-Camk2/p-DRP1 and NRF2/GPX4 Axes. *Free Radic. Biol. Med.* **213**, 150–163 (2024). 38190923 10.1016/j.freeradbiomed.2024.01.002

[133] Jing, L. et al. Quercetin inhibiting the PD-1/PD-L1 interaction for immune-enhancing cancer chemopreventive agent. *Phytother. Res.* **35**, 6441–6451 (2021). 34560814 10.1002/ptr.7297

[134] Zhang, P. et al. Quercetin attenuates the cardiotoxicity of doxorubicin-cyclophosphamide regimen and potentiates its chemotherapeutic effect against triple-negative breast cancer. *Phytother. Res.* **36**, 551–561 (2022). 34951067 10.1002/ptr.7342

[135] Chen, D. et al. Targeting the radiation-induced ARv7-mediated circNHS/miR-512-5p/XRCC5 signaling with quercetin increases prostate cancer radiosensitivity. *J. Exp. Clin. Cancer Res.* **41**, 235 (2022). 35918767 10.1186/s13046-022-02287-4 PMC9347162

[136] Biswas, P. et al. A comprehensive analysis and anti-cancer activities of quercetin in ROS-mediated cancer and cancer stem cells. *Int. J. Mol. Sci.* **23**, 11746 (2022). 10.3390/ijms231911746 PMC9569933 36233051

[137] Houghton, M. J. et al. Quercetin preserves redox status and stimulates mitochondrial function in metabolically-stressed HepG2 cells. *Free Radic. Biol. Med.* **129**, 296–309 (2018). 30266680 10.1016/j.freeradbiomed.2018.09.037

[138] Fosso, E. et al. Quercetin’s dual mode of action to counteract the Sp1-miR-27a axis in colorectal cancer cells. *Antioxidants* **12**, 1547 (2023). 10.3390/antiox12081547 PMC10451631 37627542

[139] Ye, Q., Zeng, Z., Liang, X. & Li, W. Quercetin suppresses retinoblastoma cell proliferation and invasion and facilitates oxidative stress-induced apoptosis through the miR-137/FNDC5 axis. *Environ. Res.* **237**, 116934 (2023). 37598849 10.1016/j.envres.2023.116934

[140] Chai, R. et al. Quercetin inhibits proliferation of and induces apoptosis in non-small-cell lung carcinoma via the lncRNA SNHG7/miR-34a-5p pathway. *Immunopharmacol. Immunotoxicol.* **43**, 693–703 (2021). 34448661 10.1080/08923973.2021.1966032

[141] Feng, L. et al. Long non-coding RNA Malat1 increases the rescuing effect of quercetin on TNFalpha-impaired bone marrow stem cell osteogenesis and ovariectomy-induced osteoporosis. *Int. J. Mol. Sci*. **24**, 5965 (2023). 10.3390/ijms24065965 PMC10059267 36983039

[142] Lu, X., Chen, D., Yang, F. & Xing, N. Quercetin inhibits epithelial-to-mesenchymal transition (EMT) process and promotes apoptosis in prostate cancer via downregulating lncRNA MALAT1. *Cancer Manag. Res.* **12**, 1741–1750 (2020). 32210615 10.2147/CMAR.S241093 PMC7069588

[143] Harwood, M. et al. A critical review of the data related to the safety of quercetin and lack of evidence of in vivo toxicity, including lack of genotoxic/carcinogenic properties. *Food Chem. Toxicol.* **45**, 2179–2205 (2007). 17698276 10.1016/j.fct.2007.05.015

[144] Tang, S. M. et al. Pharmacological basis and new insights of quercetin action in respect to its anti-cancer effects. *Biomed. Pharmacother.* **121**, 109604 (2020). 31733570 10.1016/j.biopha.2019.109604

[145] Santangelo, R., Silvestrini, A. & Mancuso, C. Ginsenosides, catechins, quercetin and gut microbiota: Current evidence of challenging interactions. *Food Chem. Toxicol.* **123**, 42–49 (2019). 30336256 10.1016/j.fct.2018.10.042

[146] Xu, J., Chen, H. B. & Li, S. L. Understanding the molecular mechanisms of the interplay between herbal medicines and gut microbiota. *Med. Res. Rev.* **37**, 1140–1185 (2017). 28052344 10.1002/med.21431

[147] Cialdella-Kam, L. et al. Dose-response to 3 months of quercetin-containing supplements on metabolite and quercetin conjugate profile in adults. *Br. J. Nutr.* **109**, 1923–1933 (2013). 23151341 10.1017/S0007114512003972

[148] Ashrafizadeh, M. et al. Apigenin as tumor suppressor in cancers: biotherapeutic activity, nanodelivery, and mechanisms with emphasis on pancreatic cancer. *Front. Chem.* **8**, 829 (2020). 33195038 10.3389/fchem.2020.00829 PMC7593821

[149] El Shoubaky, G. A., Abdel-Daim, M. M., Mansour, M. H. & Salem, E. A. Isolation and identification of a flavone apigenin from marine red alga *Acanthophora spicifera* with antinociceptive and anti-inflammatory activities. *J. Exp. Neurosci.* **10**, 21–29 (2016). 26917974 10.4137/JEN.S25096 PMC4760667

[150] Amini, P. et al. Induction of cancer cell death by apigenin: a review on different cell death pathways. *Mini Rev. Med. Chem.* **23**, 1461–1478 (2023). 36658710 10.2174/1389557523666230119110744

[151] Fossatelli, L., Maroccia, Z., Fiorentini, C. & Bonucci, M. Resources for human health from the plant kingdom: the potential role of the flavonoid apigenin in cancer counteraction. *Int. J. Mol. Sci.* **25**, 251 (2023). 10.3390/ijms25010251 PMC10778966 38203418

[152] Gao, A. M., Zhang, X. Y., Hu, J. N. & Ke, Z. P. Apigenin sensitizes hepatocellular carcinoma cells to doxorubic through regulating miR-520b/ATG7 axis. *Chem. Biol. Interact.* **280**, 45–50 (2018). 29191453 10.1016/j.cbi.2017.11.020

[153] Xie, Q. et al. Apigenin inhibits growth of melanoma by suppressing miR-512-3p and promoting the G1 phase of cell cycle involving the p27 Kip1 protein. *Mol. Cell Biochem.* **477**, 1569–1582 (2022). 35194732 10.1007/s11010-022-04363-x

[154] Zhao, X. et al. Apigenin suppresses proliferation, invasion, and epithelial-mesenchymal transition of cervical carcinoma cells by regulation of miR-152/BRD4 axis. *Kaohsiung J. Med. Sci.* **37**, 583–593 (2021). 33611824 10.1002/kjm2.12370 PMC11896365

[155] Shi, C. et al. LINC00629, a KLF10-responsive lncRNA, promotes the anticancer effects of apigenin by decreasing Mcl1 stability in oral squamous cell carcinoma. *Aging* **14**, 9149–9166 (2022). 36445338 10.18632/aging.204396 PMC9740369

[156] Alam, M. et al. Epigallocatechin 3-gallate: from green tea to cancer therapeutics. *Food Chem.* **379**, 132135 (2022). 35063850 10.1016/j.foodchem.2022.132135

[157] Gan, R. Y., Li, H. B., Sui, Z. Q. & Corke, H. Absorption, metabolism, anti-cancer effect and molecular targets of epigallocatechin gallate (EGCG): an updated review. *Crit. Rev. Food Sci. Nutr.* **58**, 924–941 (2018). 27645804 10.1080/10408398.2016.1231168

[158] Talib, W. H. et al. Targeting cancer hallmarks with epigallocatechin gallate (EGCG): mechanistic basis and therapeutic targets. *Molecules* **29**, 1373 (2024). 10.3390/molecules29061373 PMC10976257 38543009

[159] Lambert, J. D. & Elias, R. J. The antioxidant and pro-oxidant activities of green tea polyphenols: a role in cancer prevention. *Arch. Biochem. Biophys.* **501**, 65–72 (2010). 20558130 10.1016/j.abb.2010.06.013 PMC2946098

[160] Khandelwal, A., Hall, J. A. & Blagg, B. S. Synthesis and structure-activity relationships of EGCG analogues, a recently identified Hsp90 inhibitor. *J. Org. Chem.* **78**, 7859–7884 (2013). 23834230 10.1021/jo401027r PMC4123816

[161] Chow, H. H. et al. Pharmacokinetics and safety of green tea polyphenols after multiple-dose administration of epigallocatechin gallate and polyphenon E in healthy individuals. *Clin. Cancer Res.* **9**, 3312–3319 (2003). 12960117

[162] de la Torre, R. et al. Safety and efficacy of cognitive training plus epigallocatechin-3-gallate in young adults with Down’s syndrome (TESDAD): a double-blind, randomised, placebo-controlled, phase 2 trial. *Lancet Neurol.* **15**, 801–810 (2016). 27302362 10.1016/S1474-4422(16)30034-5

[163] Andreu-Fernandez, V. et al. Bioavailability of epigallocatechin gallate administered with different nutritional strategies in healthy volunteers. *Antioxidants* **9**, 440 (2020). 10.3390/antiox9050440 PMC7278615 32438698

[164] Joshi, P. et al. Mechanism insights of curcumin and its analogues in cancer: an update. *Phytother. Res.* **37**, 5435–5463 (2023). 37649266 10.1002/ptr.7983

[165] Zang, W. B. et al. Curcumin hybrid molecules for the treatment of Alzheimer’s disease: structure and pharmacological activities. *Eur. J. Med. Chem.* **265**, 116070 (2024). 38134747 10.1016/j.ejmech.2023.116070

[166] Wang, W. et al. Curcumin in cancer therapy: exploring molecular mechanisms and overcoming clinical challenges. *Cancer Lett.* **570**, 216332 (2023). 37541540 10.1016/j.canlet.2023.216332

[167] Wang, M. et al. Potential mechanisms of action of curcumin for cancer prevention: focus on cellular signaling pathways and miRNAs. *Int. J. Biol. Sci.* **15**, 1200–1214 (2019). 31223280 10.7150/ijbs.33710 PMC6567807

[168] Shanmugam, M. K. et al. The multifaceted role of curcumin in cancer prevention and treatment. *Molecules* **20**, 2728–2769 (2015). 25665066 10.3390/molecules20022728 PMC6272781

[169] Lao, C. D. et al. Dose escalation of a curcuminoid formulation. *BMC Complement Alter. Med.* **6**, 10 (2006). 10.1186/1472-6882-6-10 PMC1434783 16545122

[170] Dhillon, N. et al. Phase II trial of curcumin in patients with advanced pancreatic cancer. *Clin. Cancer Res.* **14**, 4491–4499 (2008). 18628464 10.1158/1078-0432.CCR-08-0024

[171] Salehi, B. et al. The therapeutic potential of curcumin: a review of clinical trials. *Eur. J. Med. Chem.* **163**, 527–545 (2019). 30553144 10.1016/j.ejmech.2018.12.016

[172] Shi, J., Zeng, Q., Liu, Y. & Pan, Z. Alternaria sp. MG1, a resveratrol-producing fungus: isolation, identification, and optimal cultivation conditions for resveratrol production. *Appl. Microbiol. Biotechnol.* **95**, 369–379 (2012). 22526800 10.1007/s00253-012-4045-9

[173] Singh, A. P. et al. Health benefits of resveratrol: evidence from clinical studies. *Med. Res. Rev.* **39**, 1851–1891 (2019). 30741437 10.1002/med.21565

[174] Chen, X. et al. Resveratrol in disease prevention and health promotion: a role of the gut microbiome. *Crit. Rev. Food Sci. Nutr*. **64**, 5878–5895 (2024). 10.1080/10408398.2022.2159921 36591813

[175] Ren, B. et al. Resveratrol for cancer therapy: challenges and future perspectives. *Cancer Lett.* **515**, 63–72 (2021). 34052324 10.1016/j.canlet.2021.05.001

[176] Rauf, A. et al. Resveratrol as an anti-cancer agent: a review. *Crit. Rev. Food Sci. Nutr.* **58**, 1428–1447 (2018). 28001084 10.1080/10408398.2016.1263597

[177] Ashrafizadeh, M. et al. Natural products and phytochemical nanoformulations targeting mitochondria in oncotherapy: an updated review on resveratrol. *Biosci. Rep*. **40**, BSR20200257 (2020). 10.1042/BSR20200257 PMC7133519 32163546

[178] Brown, V. A. et al. Repeat dose study of the cancer chemopreventive agent resveratrol in healthy volunteers: safety, pharmacokinetics, and effect on the insulin-like growth factor axis. *Cancer Res.* **70**, 9003–9011 (2010). 20935227 10.1158/0008-5472.CAN-10-2364 PMC2982884

[179] Meng, X. et al. Urinary and plasma levels of resveratrol and quercetin in humans, mice, and rats after ingestion of pure compounds and grape juice. *J. Agric. Food Chem.* **52**, 935–942 (2004). 14969553 10.1021/jf030582e

[180] Almeida, L. et al. Pharmacokinetic and safety profile of trans-resveratrol in a rising multiple-dose study in healthy volunteers. *Mol. Nutr. Food Res.* **53**, S7–S15 (2009). 19194969 10.1002/mnfr.200800177

[181] la Porte, C. et al. Steady-State pharmacokinetics and tolerability of trans-resveratrol 2000 mg twice daily with food, quercetin and alcohol (ethanol) in healthy human subjects. *Clin. Pharmacokinet.* **49**, 449–454 (2010). 20528005 10.2165/11531820-000000000-00000

[182] Abd El-Mohsen, M. et al. Distribution of [3H]trans-resveratrol in rat tissues following oral administration. *Br. J. Nutr.* **96**, 62–70 (2006). 16869992 10.1079/bjn20061810

[183] Walle, T. et al. High absorption but very low bioavailability of oral resveratrol in humans. *Drug Metab. Dispos.* **32**, 1377–1382 (2004). 15333514 10.1124/dmd.104.000885

[184] Drabinska, N. & Jarocka-Cyrta, E. Crosstalk between resveratrol and gut barrier: a review. *Int. J. Mol. Sci*. **23**, 15279 (2022). 10.3390/ijms232315279 PMC9739931 36499603

[185] Prakash, V. et al. Resveratrol as a promising nutraceutical: implications in gut microbiota modulation, inflammatory disorders, and colorectal cancer. *Int. J. Mol. Sci*. **25**, 3370 (2024). 10.3390/ijms25063370 PMC10970219 38542344

[186] Wang, P. et al. Targeting microbiota-host interactions with resveratrol on cancer: effects and potential mechanisms of action. *Crit. Rev. Food Sci. Nutr.* **64**, 311–333 (2024). 35917112 10.1080/10408398.2022.2106180

[187] Di Dalmazi, G. et al. Promising role of alkaloids in the prevention and treatment of thyroid cancer and autoimmune thyroid disease: a comprehensive review of the current evidence. *Int. J. Mol. Sci.* **25**, 5395 (2024). 10.3390/ijms25105395 PMC11121374 38791433

[188] Mondal, A. et al. Alkaloids for cancer prevention and therapy: current progress and future perspectives. *Eur. J. Pharm.* **858**, 172472 (2019). 10.1016/j.ejphar.2019.172472 31228447

[189] Efferth, T. & Oesch, F. Repurposing of plant alkaloids for cancer therapy: pharmacology and toxicology. *Semin Cancer Biol.* **68**, 143–163 (2021). 31883912 10.1016/j.semcancer.2019.12.010

[190] Schultz, A. G. Camptothecin. *Chem. Rev.* **73**, 385–405 (1973). 4578778 10.1021/cr60284a004

[191] Wilkes, E. Vinblastine. *Lancet* **1**, 1390 (1964). 14184159

[192] Li, F., Jiang, T., Li, Q. & Ling, X. Camptothecin (CPT) and its derivatives are known to target topoisomerase I (Top1) as their mechanism of action: did we miss something in CPT analogue molecular targets for treating human disease such as cancer? *Am. J. Cancer Res.* **7**, 2350–2394 (2017). 29312794 PMC5752681

[193] Martino, E. et al. The long story of camptothecin: from traditional medicine to drugs. *Bioorg. Med. Chem. Lett.* **27**, 701–707 (2017). 28073672 10.1016/j.bmcl.2016.12.085

[194] Pommier, Y. Topoisomerase I inhibitors: camptothecins and beyond. *Nat. Rev. Cancer* **6**, 789–802 (2006). 16990856 10.1038/nrc1977

[195] Venditto, V. J. & Simanek, E. E. Cancer therapies utilizing the camptothecins: a review of the in vivo literature. *Mol. Pharm.* **7**, 307–349 (2010). 20108971 10.1021/mp900243b PMC3733266

[196] Chu, B. et al. Stimulus-responsive nano-prodrug strategies for cancer therapy: a focus on camptothecin delivery. *Small Methods* **8**, 2301271 (2024). 10.1002/smtd.202301271 38085682

[197] Moudi, M., Go, R., Yien, C. Y. & Nazre, M. Vinca alkaloids. *Int. J. Prev. Med.* **4**, 1231–1235 (2013). 24404355 PMC3883245

[198] Banyal, A. et al. Vinca alkaloids as a potential cancer therapeutics: recent update and future challenges. *3 Biotech* **13**, 211 (2023). 37251731 10.1007/s13205-023-03636-6 PMC10209376

[199] Haque, A., Rahman, M. A., Faizi, M. S. H. & Khan, M. S. Next generation antineoplastic agents: a review on structurally modified vinblastine (VBL) analogues. *Curr. Med. Chem.* **25**, 1650–1662 (2018). 28464783 10.2174/0929867324666170502123639

[200] Himes, R. H. Interactions of the Catharanthus (Vinca) alkaloids with tubulin and microtubules. *Pharm. Ther.* **51**, 257–267 (1991). 10.1016/0163-7258(91)90081-v 1784631

[201] Liu, Y. et al. Berberine diminishes cancer cell PD-L1 expression and facilitates antitumor immunity via inhibiting the deubiquitination activity of CSN5. *Acta Pharm. Sin. B* **10**, 2299–2312 (2020). 33354502 10.1016/j.apsb.2020.06.014 PMC7745128

[202] Kwon, S. & Chan, A. T. Extracting the benefits of berberine for colorectal cancer. *Lancet Gastroenterol. Hepatol.* **5**, 231–233 (2020). 31926919 10.1016/S2468-1253(19)30430-3

[203] Liu, L. et al. Berberine in combination with cisplatin induces necroptosis and apoptosis in ovarian cancer cells. *Biol. Res.* **52**, 37 (2019). 31319879 10.1186/s40659-019-0243-6 PMC6637630

[204] Zhang, Q. et al. Berberine represses human gastric cancer cell growth in vitro and in vivo by inducing cytostatic autophagy via inhibition of MAPK/mTOR/p70S6K and Akt signaling pathways. *Biomed. Pharmacother.* **128**, 110245 (2020). 32454290 10.1016/j.biopha.2020.110245

[205] Xia, Y. et al. Berberine suppresses bladder cancer cell proliferation by inhibiting JAK1-STAT3 signaling via upregulation of miR-17-5p. *Biochem Pharm.* **188**, 114575 (2021). 33887260 10.1016/j.bcp.2021.114575

[206] Deng, J. et al. Pre-administration of berberine exerts chemopreventive effects in AOM/DSS-induced colitis-associated carcinogenesis mice via modulating inflammation and intestinal microbiota. *Nutrients* **14**, 726 (2022). 10.3390/nu14040726 PMC8879943 35215376

[207] Tang, Z. et al. Berberine hydrochloride-loaded dung beetle chitosan/sodium alginate microspheres ameliorate DSS-induced colitis and regulate gut microorganisms in mice. *Int. J. Biol. Macromol.* **255**, 128219 (2024). 37981270 10.1016/j.ijbiomac.2023.128219

[208] Zheng, C. et al. Berberine inhibits dendritic cells differentiation in DSS-induced colitis by promoting *Bacteroides fragilis*. *Int. Immunopharmacol.* **101**, 108329 (2021). 34749293 10.1016/j.intimp.2021.108329

[209] Cui, H. et al. Berberine regulates Treg/Th17 balance to treat ulcerative colitis through modulating the gut microbiota in the colon. *Front. Pharm.* **9**, 571 (2018). 10.3389/fphar.2018.00571 PMC5991375 29904348

[210] Feng, Y. et al. Berberine: Potential preventive and therapeutic strategies for human colorectal cancer. *Cell Biochem. Funct.* **42**, e4033 (2024). 38742849 10.1002/cbf.4033

[211] Nazha, A., Kantarjian, H., Cortes, J. & Quintas-Cardama, A. Omacetaxine mepesuccinate (synribo)—newly launched in chronic myeloid leukemia. *Expert Opin. Pharmacother.* **14**, 1977–1986 (2013). 23875628 10.1517/14656566.2013.821464

[212] Bohlander, S. K. A new kid on the block for acute myeloid leukemia treatment? Homoharringtonine interferes with key pathways in acute myeloid leukemia cells. *Haematologica* **105**, 7–9 (2020). 31894095 10.3324/haematol.2019.234880 PMC6939538

[213] Khatua, S. et al. Homoharringtonine: updated insights into its efficacy in hematological malignancies, diverse cancers and other biomedical applications. *Eur. J. Med. Res.* **29**, 269 (2024). 38704602 10.1186/s40001-024-01856-x PMC11069164

[214] Lu, S. & Wang, J. Homoharringtonine and omacetaxine for myeloid hematological malignancies. *J. Hematol. Oncol.* **7**, 2 (2014). 24387717 10.1186/1756-8722-7-2 PMC3884015

[215] Zhou, H. et al. Targeting of phospho-eIF4E by homoharringtonine eradicates a distinct subset of human acute myeloid leukemia. *Leuk. Lymphoma* **61**, 1084–1096 (2020). 29334312 10.1080/10428194.2017.1390229

[216] Wu, Q. et al. Inhibition of bladder cancer growth with homoharringtonine by inactivating integrin alpha5/beta1-FAK/Src axis: a novel strategy for drug application. *Pharm. Res.* **188**, 106654 (2023). 10.1016/j.phrs.2023.106654 36640858

[217] Wang, L. B. et al. Homoharringtonine inhibited breast cancer cells growth via miR-18a-3p/AKT/mTOR signaling pathway. *Int. J. Biol. Sci.* **17**, 995–1009 (2021). 33867824 10.7150/ijbs.44907 PMC8040299

[218] Xi, C. et al. Repurposing homoharringtonine for thyroid cancer treatment through TIMP1/FAK/PI3K/AKT signaling pathway. *iScience* **27**, 109829 (2024). 38770133 10.1016/j.isci.2024.109829 PMC11103377

[219] Yakhni, M. et al. Homoharringtonine, an approved anti-leukemia drug, suppresses triple negative breast cancer growth through a rapid reduction of anti-apoptotic protein abundance. *Am. J. Cancer Res.* **9**, 1043–1060 (2019). 31218111 PMC6556597

[220] Porcu, E. et al. Identification of Homoharringtonine as a potent inhibitor of glioblastoma cell proliferation and migration. *Transl. Res.* **251**, 41–53 (2023). 35788055 10.1016/j.trsl.2022.06.017

[221] Yadav, S. S. et al. Therapeutic spectrum of piperine for clinical practice: a scoping review. *Crit. Rev. Food Sci. Nutr.* **63**, 5813–5840 (2023). 34996326 10.1080/10408398.2021.2024792

[222] Rather, R. A. & Bhagat, M. Cancer chemoprevention and piperine: molecular mechanisms and therapeutic opportunities. *Front. Cell Dev. Biol.* **6**, 10 (2018). 29497610 10.3389/fcell.2018.00010 PMC5818432

[223] Cui, T. et al. Piperine is a mechanism-based inactivator of CYP3A. *Drug Metab. Dispos.* **48**, 123–134 (2020). 31748224 10.1124/dmd.119.088955

[224] Pannu, N. & Bhatnagar, A. Combinatorial therapeutic effect of resveratrol and piperine on murine model of systemic lupus erythematosus. *Inflammopharmacology* **28**, 401–424 (2020). 31732838 10.1007/s10787-019-00662-w

[225] Tripathi, A. K., Ray, A. K. & Mishra, S. K. Molecular and pharmacological aspects of piperine as a potential molecule for disease prevention and management: evidence from clinical trials. *Beni-Suef Univ. J. Basic Appl. Sci.* **11**, 16 (2022). 35127957 10.1186/s43088-022-00196-1 PMC8796742

[226] Haq, I. U. et al. Piperine: a review of its biological effects. *Phytother. Res.* **35**, 680–700 (2021). 32929825 10.1002/ptr.6855

[227] Quijia, C. R. & Chorilli, M. Piperine for treating breast cancer: a review of molecular mechanisms, combination with anticancer drugs, and nanosystems. *Phytother. Res.* **36**, 147–163 (2022). 34559416 10.1002/ptr.7291

[228] Ahmed Khalil, A. et al. Recent developments and anticancer therapeutics of paclitaxel: an update. *Curr. Pharm. Des.* **28**, 3363–3373 (2022). 36330627 10.2174/1381612829666221102155212

[229] Zhu, L. & Chen, L. Progress in research on paclitaxel and tumor immunotherapy. *Cell Mol. Biol. Lett.* **24**, 40 (2019). 31223315 10.1186/s11658-019-0164-y PMC6567594

[230] Bernabeu, E. et al. Paclitaxel: what has been done and the challenges remain ahead. *Int. J. Pharm.* **526**, 474–495 (2017). 28501439 10.1016/j.ijpharm.2017.05.016

[231] Tang, W. et al. Paclitaxel nanoparticle awakens immune system to fight against cancer. *Nanoscale* **9**, 6529–6536 (2017). 28466929 10.1039/c6nr09895a

[232] Tu, Y. Artemisinin-A gift from traditional Chinese medicine to the world (Nobel lecture). *Angew. Chem. Int. Ed. Engl.* **55**, 10210–10226 (2016). 27488942 10.1002/anie.201601967

[233] Efferth, T. From ancient herb to modern drug: artemisia annua and artemisinin for cancer therapy. *Semin. Cancer Biol.* **46**, 65–83 (2017). 28254675 10.1016/j.semcancer.2017.02.009

[234] Wong, Y. K. et al. Artemisinin as an anticancer drug: recent advances in target profiling and mechanisms of action. *Med. Res. Rev.* **37**, 1492–1517 (2017). 28643446 10.1002/med.21446

[235] Efferth, T. et al. Enhancement of cytotoxicity of artemisinins toward cancer cells by ferrous iron. *Free Radic. Biol. Med.* **37**, 998–1009 (2004). 15336316 10.1016/j.freeradbiomed.2004.06.023

[236] Lai, H., Sasaki, T. & Singh, N. P. Targeted treatment of cancer with artemisinin and artemisinin-tagged iron-carrying compounds. *Expert Opin. Ther. Targets* **9**, 995–1007 (2005). 16185154 10.1517/14728222.9.5.995

[237] Lai, H., Sasaki, T., Singh, N. P. & Messay, A. Effects of artemisinin-tagged holotransferrin on cancer cells. *Life Sci.* **76**, 1267–1279 (2005). 15642597 10.1016/j.lfs.2004.08.020

[238] Zhu, S. et al. Ferroptosis: a novel mechanism of artemisinin and its derivatives in cancer therapy. *Curr. Med. Chem.* **28**, 329–345 (2021). 31965935 10.2174/0929867327666200121124404

[239] Posadino, A. M. et al. Medicinal and mechanistic overview of artemisinin in the treatment of human diseases. *Biomed. Pharmacother.* **163**, 114866 (2023). 37182516 10.1016/j.biopha.2023.114866

[240] Chen, G. Q. et al. Artemisinin compounds sensitize cancer cells to ferroptosis by regulating iron homeostasis. *Cell Death Differ.* **27**, 242–254 (2020). 31114026 10.1038/s41418-019-0352-3 PMC7205875

[241] Li, Z. et al. Artemisinin and its derivatives as a repurposing anticancer agent: what else do we need to do? *Molecules* **21**, 1331 (2016). 10.3390/molecules21101331 PMC6272993 27739410

[242] Kiani, B. H. et al. Artemisinin and its derivatives: a promising cancer therapy. *Mol. Biol. Rep.* **47**, 6321–6336 (2020). 32710388 10.1007/s11033-020-05669-z

[243] Soo, H. L. et al. Advances and challenges in developing andrographolide and its analogues as cancer therapeutic agents. *Drug Discov. Today* **24**, 1890–1898 (2019). 31154065 10.1016/j.drudis.2019.05.017

[244] Islam, M. T. Andrographolide, a new hope in the prevention and treatment of metabolic syndrome. *Front Pharm.* **8**, 571 (2017). 10.3389/fphar.2017.00571 PMC5572404 28878680

[245] Bera, R. et al. Pharmacokinetic analysis and tissue distribution of andrographolide in rat by a validated LC-MS/MS method. *Pharm. Biol.* **52**, 321–329 (2014). 24171780 10.3109/13880209.2013.836544

[246] Xu, L. et al. A simple and sensitive HPLC-ESI-MS/MS method for the determination of andrographolide in human plasma. *J. Chromatogr. B Anal. Technol. Biomed. Life Sci.* **877**, 502–506 (2009). 10.1016/j.jchromb.2008.12.065 19158000

[247] Sandhu, S. S. et al. Ursolic acid: a pentacyclic triterpenoid that exhibits anticancer therapeutic potential by modulating multiple oncogenic targets. *Biotechnol. Genet. Eng. Rev.* **39**, 729–759 (2023). 36600517 10.1080/02648725.2022.2162257

[248] Zou, J. et al. Ursolic acid in cancer treatment and metastatic chemoprevention: from synthesized derivatives to nanoformulations in preclinical studies. *Curr. Cancer Drug Targets* **19**, 245–256 (2019). 30332961 10.2174/1568009618666181016145940

[249] Nguyen, H. N. et al. Ursolic acid and related analogues: triterpenoids with broad health benefits. *Antioxidants* **10**, 1161 (2021). 10.3390/antiox10081161 PMC8388988 34439409

[250] Limami, Y. et al. Ursolic acid’s alluring journey: one triterpenoid vs. cancer hallmarks. *Molecules* **28**, 7897 (2023). 10.3390/molecules28237897 PMC10707789 38067626

[251] Chen, Q., Luo, S., Zhang, Y. & Chen, Z. Development of a liquid chromatography-mass spectrometry method for the determination of ursolic acid in rat plasma and tissue: application to the pharmacokinetic and tissue distribution study. *Anal. Bioanal. Chem.* **399**, 2877–2884 (2011). 21249342 10.1007/s00216-011-4651-x

[252] Iqbal, J. et al. Ursolic acid a promising candidate in the therapeutics of breast cancer: current status and future implications. *Biomed. Pharmacother.* **108**, 752–756 (2018). 30248543 10.1016/j.biopha.2018.09.096

[253] Chikara, S. et al. Oxidative stress and dietary phytochemicals: role in cancer chemoprevention and treatment. *Cancer Lett.* **413**, 122–134 (2018). 29113871 10.1016/j.canlet.2017.11.002

[254] Landis-Piwowar, K. R. & Iyer, N. R. Cancer chemoprevention: current state of the art. *Cancer Growth Metastasis* **7**, 19–25 (2014). 24987270 10.4137/CGM.S11288 PMC4064948

[255] Ma, L. et al. Plant natural products: promising resources for cancer chemoprevention. *Molecules* **26**, 933 (2021). 10.3390/molecules26040933 PMC7916513 33578780

[256] Braicu, C. et al. A comprehensive review on MAPK: a promising therapeutic target in cancer. *Cancers* **11**, 1618 (2019). 10.3390/cancers11101618 PMC6827047 31652660

[257] Park, H. B. & Baek, K. H. E3 ligases and deubiquitinating enzymes regulating the MAPK signaling pathway in cancers. *Biochim. Biophys. Acta Rev. Cancer* **1877**, 188736 (2022). 35589008 10.1016/j.bbcan.2022.188736

[258] Bahar, M. E., Kim, H. J. & Kim, D. R. Targeting the RAS/RAF/MAPK pathway for cancer therapy: from mechanism to clinical studies. *Signal Transduct. Target Ther.* **8**, 455 (2023). 38105263 10.1038/s41392-023-01705-z PMC10725898

[259] Davis, R. J. Signal transduction by the JNK group of MAP kinases. *Cell* **103**, 239–252 (2000). 11057897 10.1016/s0092-8674(00)00116-1

[260] Yue, J. & Lopez, J. M. Understanding MAPK signaling pathways in apoptosis. *Int. J. Mol. Sci*. **21**, 2346 (2020). 10.3390/ijms21072346 PMC7177758 32231094

[261] Johnson, G. L. & Lapadat, R. Mitogen-activated protein kinase pathways mediated by ERK, JNK, and p38 protein kinases. *Science* **298**, 1911–1912 (2002). 12471242 10.1126/science.1072682

[262] Wei, J. et al. MAPK signaling pathway-targeted marine compounds in cancer therapy. *J. Cancer Res Clin. Oncol.* **147**, 3–22 (2021). 33389079 10.1007/s00432-020-03460-y PMC11801988

[263] Yuan, J., Dong, X., Yap, J. & Hu, J. The MAPK and AMPK signalings: interplay and implication in targeted cancer therapy. *J. Hematol. Oncol.* **13**, 113 (2020). 32807225 10.1186/s13045-020-00949-4 PMC7433213

[264] Liu, F., Yang, X., Geng, M. & Huang, M. Targeting ERK, an Achilles’ Heel of the MAPK pathway, in cancer therapy. *Acta Pharm. Sin. B* **8**, 552–562 (2018). 30109180 10.1016/j.apsb.2018.01.008 PMC6089851

[265] Kciuk, M. et al. Metastasis and MAPK pathways. *Int. J. Mol. Sci.* **23**, 3847 (2022). 10.3390/ijms23073847 PMC8998814 35409206

[266] Sui, X. et al. p38 and JNK MAPK pathways control the balance of apoptosis and autophagy in response to chemotherapeutic agents. *Cancer Lett.* **344**, 174–179 (2014). 24333738 10.1016/j.canlet.2013.11.019

[267] Subauste, M. C. et al. Vinculin modulation of paxillin-FAK interactions regulates ERK to control survival and motility. *J. Cell Biol.* **165**, 371–381 (2004). 15138291 10.1083/jcb.200308011 PMC2172187

[268] Cui, S. et al. Genistein inhibits the growth and regulates the migration and invasion abilities of melanoma cells via the FAK/paxillin and MAPK pathways. *Oncotarget* **8**, 21674–21691 (2017). 28423510 10.18632/oncotarget.15535 PMC5400615

[269] Chen, C. et al. Genistein inhibits migration and invasion of cervical cancer HeLa cells by regulating FAK-paxillin and MAPK signaling pathways. *Taiwan J. Obstet. Gynecol.* **59**, 403–408 (2020). 32416888 10.1016/j.tjog.2020.03.012

[270] Briones-Orta, M. A. et al. Osteopontin splice variants and polymorphisms in cancer progression and prognosis. *Biochim. Biophys. Acta Rev. Cancer* **1868**, 93–108 A (2017). 28254527 10.1016/j.bbcan.2017.02.005

[271] Khongsti, K., Das, K. B. & Das, B. MAPK pathway and SIRT1 are involved in the down-regulation of secreted osteopontin expression by genistein in metastatic cancer cells. *Life Sci.* **265**, 118787 (2021). 33249095 10.1016/j.lfs.2020.118787

[272] Erdogan, S. et al. Midkine downregulation increases the efficacy of quercetin on prostate cancer stem cell survival and migration through PI3K/AKT and MAPK/ERK pathway. *Biomed. Pharmacother.* **107**, 793–805 (2018). 30142541 10.1016/j.biopha.2018.08.061

[273] Kim, S. H. et al. Antitumor and apoptotic effects of quercetin on human melanoma cells involving JNK/P38 MAPK signaling activation. *Eur. J. Pharm.* **860**, 172568 (2019). 10.1016/j.ejphar.2019.172568 31348906

[274] Xuan, Y. Z., Xu, L. Z., Jin, C. R. & Yang, K. J. Apigenin inhibits proliferation, migration, and invasion of human tongue carcinoma Tca8113 cells through regulating the MAPK signaling pathways. *Curr. Mol. Med.* **21**, 690–697 (2021). 33092506 10.2174/1566524020666201022120420

[275] Li, Y. et al. Apigenin, a flavonoid constituent derived from *P. villosa*, inhibits hepatocellular carcinoma cell growth by CyclinD1/CDK4 regulation via p38 MAPK-p21 signaling. *Pathol. Res. Pr.* **216**, 152701 (2020). 10.1016/j.prp.2019.152701 31780054

[276] Xiao, X. et al. (-)-Epigallocatechin-3-gallate induces cell apoptosis in chronic myeloid leukaemia by regulating Bcr/Abl-mediated p38-MAPK/JNK and JAK2/STAT3/AKT signalling pathways. *Clin. Exp. Pharm. Physiol.* **46**, 126–136 (2019). 10.1111/1440-1681.13037 30251267

[277] Wu, M. F. et al. Curcumin induces apoptosis of chemoresistant lung cancer cells via ROS-regulated p38 MAPK phosphorylation. *Int. J. Mol. Sci.* **23**, 8248 (2022). 10.3390/ijms23158248 PMC9367815 35897820

[278] Zhu, G. et al. Curcumin inhibited the growth and invasion of human monocytic leukaemia SHI-1 cells in vivo by altering MAPK and MMP signalling. *Pharm. Biol.* **58**, 25–34 (2020). 31854220 10.1080/13880209.2019.1701042 PMC6968541

[279] Lim, W., Jeong, M., Bazer, F. W. & Song, G. Curcumin suppresses proliferation and migration and induces apoptosis on human placental choriocarcinoma cells via ERK1/2 and SAPK/JNK MAPK signaling pathways. *Biol. Reprod.* **95**, 83 (2016). 27580989 10.1095/biolreprod.116.141630

[280] Yuan, S. X. et al. BMP9/p38 MAPK is essential for the antiproliferative effect of resveratrol on human colon cancer. *Oncol. Rep.* **35**, 939–947 (2016). 26555012 10.3892/or.2015.4407

[281] Cao, L. et al. Resveratrol inhibits hyperglycemia-driven ROS-induced invasion and migration of pancreatic cancer cells via suppression of the ERK and p38 MAPK signaling pathways. *Int. J. Oncol.* **49**, 735–743 (2016). 27278736 10.3892/ijo.2016.3559

[282] Wang, J. et al. Resveratrol, an activator of SIRT1, induces protective autophagy in non-small-cell lung cancer via inhibiting Akt/mTOR and activating p38-MAPK. *OncoTargets Ther.* **11**, 7777–7786 (2018). 10.2147/OTT.S159095 PMC6223384 30464525

[283] Si, L., Yang, R., Lin, R. & Yang S. Piperine functions as a tumor suppressor for human ovarian tumor growth via activation of JNK/p38 MAPK-mediated intrinsic apoptotic pathway. *Biosci. Rep.* **38**, BSR20180503 (2018). 10.1042/BSR20180503 PMC6435525 29717031

[284] Guo, Y. et al. Interleukin-6 signaling pathway in targeted therapy for cancer. *Cancer Treat. Rev.* **38**, 904–910 (2012). 22651903 10.1016/j.ctrv.2012.04.007

[285] Jones, S. A. & Jenkins, B. J. Recent insights into targeting the IL-6 cytokine family in inflammatory diseases and cancer. *Nat. Rev. Immunol.* **18**, 773–789 (2018). 30254251 10.1038/s41577-018-0066-7

[286] Xia, Y. et al. Piperine inhibits IL-1beta-induced IL-6 expression by suppressing p38 MAPK and STAT3 activation in gastric cancer cells. *Mol. Cell Biochem.* **398**, 147–156 (2015). 25234193 10.1007/s11010-014-2214-0

[287] Li, H. L. et al. MAPK pathways are involved in the inhibitory effect of berberine hydrochloride on gastric cancer MGC 803 cell proliferation and IL-8 secretion in vitro and in vivo. *Mol. Med. Rep.* **14**, 1430–1438 (2016). 27278862 10.3892/mmr.2016.5361

[288] Gao, X. et al. Berberine and cisplatin exhibit synergistic anticancer effects on osteosarcoma MG-63 cells by inhibiting the MAPK pathway. *Molecules* **26**, 1666 (2021). 10.3390/molecules26061666 PMC8002572 33802664

[289] Zheng, F. et al. p38alpha MAPK-mediated induction and interaction of FOXO3a and p53 contribute to the inhibited-growth and induced-apoptosis of human lung adenocarcinoma cells by berberine. *J. Exp. Clin. Cancer Res.* **33**, 36 (2014). 24766860 10.1186/1756-9966-33-36 PMC4013801

[290] Shi, X. et al. Homoharringtonine suppresses LoVo cell growth by inhibiting EphB4 and the PI3K/AKT and MAPK/EKR1/2 signaling pathways. *Food Chem. Toxicol.* **136**, 110960 (2020). 31726078 10.1016/j.fct.2019.110960

[291] Yang, T., Yao, S., Zhang, X. & Guo, Y. Andrographolide inhibits growth of human T-cell acute lymphoblastic leukemia Jurkat cells by downregulation of PI3K/AKT and upregulation of p38 MAPK pathways. *Drug Des. Dev. Ther.* **10**, 1389–1397 (2016). 10.2147/DDDT.S94983 PMC4833376 27114702

[292] Lee, K. C., Chen, Y. L., Lin, P. Y. & Chuang, W. L. Ursolic acid-induced apoptosis via regulation of the PI3K/Akt and MAPK signaling pathways in Huh-7 cells. *Molecules* **23**, 2016 (2018). 10.3390/molecules23082016 PMC6222435 30104508

[293] Chuang, W. L., Lin, P. Y., Lin, H. C. & Chen, Y. L. The apoptotic effect of ursolic acid on SK-Hep-1 cells is regulated by the PI3K/Akt, p38 and JNK MAPK signaling pathways. *Molecules* **21**, 460 (2016). 27104510 10.3390/molecules21040460 PMC6274268

[294] Wu, C. C. et al. Ursolic acid triggers apoptosis in human osteosarcoma cells via caspase activation and the ERK1/2 MAPK pathway. *J. Agric. Food Chem.* **64**, 4220–4226 (2016). 27171502 10.1021/acs.jafc.6b00542

[295] Li, Z., Ding, X., Wu, H. & Liu, C. Artemisinin inhibits angiogenesis by regulating p38 MAPK/CREB/TSP-1 signaling pathway in osteosarcoma. *J. Cell Biochem.* **120**, 11462–11470 (2019). 30746754 10.1002/jcb.28424

[296] Glaviano, A. et al. PI3K/AKT/mTOR signaling transduction pathway and targeted therapies in cancer. *Mol. Cancer* **22**, 138 (2023). 37596643 10.1186/s12943-023-01827-6 PMC10436543

[297] Janku, F., Yap, T. A. & Meric-Bernstam, F. Targeting the PI3K pathway in cancer: are we making headway? *Nat. Rev. Clin. Oncol.* **15**, 273–291 (2018). 29508857 10.1038/nrclinonc.2018.28

[298] Browne, I. M. et al. Optimal targeting of PI3K-AKT and mTOR in advanced oestrogen receptor-positive breast cancer. *Lancet Oncol.* **25**, e139–e151 (2024). 38547898 10.1016/S1470-2045(23)00676-9

[299] Yu, L., Wei, J. & Liu, P. Attacking the PI3K/Akt/mTOR signaling pathway for targeted therapeutic treatment in human cancer. *Semin Cancer Biol.* **85**, 69–94 (2022). 34175443 10.1016/j.semcancer.2021.06.019

[300] Liu, R. et al. PI3K/AKT pathway as a key link modulates the multidrug resistance of cancers. *Cell Death Dis.* **11**, 797 (2020). 32973135 10.1038/s41419-020-02998-6 PMC7515865

[301] He, Y. et al. Targeting PI3K/Akt signal transduction for cancer therapy. *Signal Transduct. Target Ther.* **6**, 425 (2021). 34916492 10.1038/s41392-021-00828-5 PMC8677728

[302] Chamcheu, J. C. et al. Role and therapeutic targeting of the PI3K/Akt/mTOR signaling pathway in skin cancer: a review of current status and future trends on natural and synthetic agents therapy. *Cells* **8**, 803 (2019). 10.3390/cells8080803 PMC6721560 31370278

[303] Yuan, Y. et al. PI3K-AKT-targeting breast cancer treatments: natural products and synthetic compounds. *Biomolecules* **13**, 93 (2023). 10.3390/biom13010093 PMC9856042 36671478

[304] Tewari, D. et al. Natural products targeting the PI3K-Akt-mTOR signaling pathway in cancer: a novel therapeutic strategy. *Semin. Cancer Biol.* **80**, 1–17 (2022). 31866476 10.1016/j.semcancer.2019.12.008

[305] Chan, K. K. L. et al. Estrogen receptor modulators genistein, daidzein and ERB-041 inhibit cell migration, invasion, proliferation and sphere formation via modulation of FAK and PI3K/AKT signaling in ovarian cancer. *Cancer Cell Int.* **18**, 65 (2018). 29743815 10.1186/s12935-018-0559-2 PMC5930957

[306] Park, C. et al. Induction of G2/M cell cycle arrest and apoptosis by genistein in human bladder cancer T24 cells through inhibition of the ROS-dependent PI3k/Akt signal transduction pathway. *Antioxidants* **8**, 327 (2019). 10.3390/antiox8090327 PMC6769882 31438633

[307] Li, X. et al. Quercetin suppresses breast cancer stem cells (CD44(+)/CD24(-)) by inhibiting the PI3K/Akt/mTOR-signaling pathway. *Life Sci.* **196**, 56–62 (2018). 29355544 10.1016/j.lfs.2018.01.014

[308] Granato, M. et al. Quercetin induces apoptosis and autophagy in primary effusion lymphoma cells by inhibiting PI3K/AKT/mTOR and STAT3 signaling pathways. *J. Nutr. Biochem.* **41**, 124–136 (2017). 28092744 10.1016/j.jnutbio.2016.12.011

[309] Yang, J., Pi, C. & Wang, G. Inhibition of PI3K/Akt/mTOR pathway by apigenin induces apoptosis and autophagy in hepatocellular carcinoma cells. *Biomed. Pharmacother.* **103**, 699–707 (2018). 29680738 10.1016/j.biopha.2018.04.072

[310] Tian, B. et al. Curcumin inhibits urothelial tumor development by suppressing IGF2 and IGF2-mediated PI3K/AKT/mTOR signaling pathway. *J. Drug Target* **25**, 626–636 (2017). 28286973 10.1080/1061186X.2017.1306535

[311] Liu, F. et al. Antitumor activity of curcumin by modulation of apoptosis and autophagy in human lung cancer A549 cells through inhibiting PI3K/Akt/mTOR pathway. *Oncol. Rep.* **39**, 1523–1531 (2018). 29328421 10.3892/or.2018.6188

[312] Zhu, X. & Zhu, R. Curcumin suppresses the progression of laryngeal squamous cell carcinoma through the upregulation of miR-145 and inhibition of the PI3K/Akt/mTOR pathway. *OncoTargets Ther.* **11**, 3521–3531 (2018). 10.2147/OTT.S159236 PMC6016259 29950857

[313] Han, E. J. et al. Piperine induces apoptosis and autophagy in HSC-3 human oral cancer cells by regulating PI3K signaling pathway. *Int. J. Mol. Sci.* **24**, 13949 (2023). 10.3390/ijms241813949 PMC10530752 37762259

[314] Li, G. et al. Berberine regulates the Notch1/PTEN/PI3K/AKT/mTOR pathway and acts synergistically with 17-AAG and SAHA in SW480 colon cancer cells. *Pharm. Biol.* **59**, 21–30 (2021). 33417512 10.1080/13880209.2020.1865407 PMC7808376

[315] Mohiuddin, M. & Kasahara, K. Paclitaxel impedes EGFR-mutated PC9 cell growth via reactive oxygen species-mediated DNA damage and EGFR/PI3K/AKT/mTOR signaling pathway suppression. *Cancer Genomics Proteom.* **18**, 645–659 (2021). 10.21873/cgp.20287 PMC8441765 34479917

[316] Tohkayomatee, R. et al. Andrographolide exhibits anticancer activity against breast cancer cells (MCF-7 and MDA-MB-231 cells) through suppressing cell proliferation and inducing cell apoptosis via inactivation of ER-alpha receptor and PI3K/AKT/mTOR signaling. *Molecules* **27**, 3544 (2022). 10.3390/molecules27113544 PMC9182433 35684480

[317] Meng, Y. et al. Ursolic acid induces apoptosis of prostate cancer cells via the PI3K/Akt/mTOR pathway. *Am. J. Chin. Med.* **43**, 1471–1486 (2015). 26503559 10.1142/S0192415X15500834

[318] Farhan, M. et al. Artemisinin inhibits the migration and invasion in uveal melanoma via inhibition of the PI3K/AKT/mTOR signaling pathway. *Oxid. Med. Cell Longev.* **2021**, 9911537 (2021). 34931134 10.1155/2021/9911537 PMC8684509

[319] Liu, J. et al. Wnt/beta-catenin signalling: function, biological mechanisms, and therapeutic opportunities. *Signal Transduct. Target Ther.* **7**, 3 (2022). 34980884 10.1038/s41392-021-00762-6 PMC8724284

[320] Zhang, Y. & Wang, X. Targeting the Wnt/beta-catenin signaling pathway in cancer. *J. Hematol. Oncol.* **13**, 165 (2020). 33276800 10.1186/s13045-020-00990-3 PMC7716495

[321] Zhao, H. et al. Wnt signaling in colorectal cancer: pathogenic role and therapeutic target. *Mol. Cancer* **21**, 144 (2022). 35836256 10.1186/s12943-022-01616-7 PMC9281132

[322] Cruciat, C. M. & Niehrs, C. Secreted and transmembrane wnt inhibitors and activators. *Cold Spring Harb. Perspect. Biol.* **5**, a015081 (2013). 23085770 10.1101/cshperspect.a015081 PMC3578365

[323] Reyes, M. et al. Wnt/beta-catenin signaling in oral carcinogenesis. *Int. J. Mol. Sci.* **21**, 4682 (2020). 32630122 10.3390/ijms21134682 PMC7369957

[324] Baruah, M. M., Khandwekar, A. P. & Sharma, N. Quercetin modulates Wnt signaling components in prostate cancer cell line by inhibiting cell viability, migration, and metastases. *Tumour Biol.* **37**, 14025–14034 (2016). 27495232 10.1007/s13277-016-5277-6

[325] Xu, M. et al. Apigenin suppresses colorectal cancer cell proliferation, migration and invasion via inhibition of the Wnt/beta-catenin signaling pathway. *Oncol. Lett.* **11**, 3075–3080 (2016). 27123066 10.3892/ol.2016.4331 PMC4840993

[326] Pan, F. F. et al. H19-Wnt/beta-catenin regulatory axis mediates the suppressive effects of apigenin on tumor growth in hepatocellular carcinoma. *Eur. J. Pharm.* **893**, 173810 (2021). 10.1016/j.ejphar.2020.173810 33345859

[327] Zhu, J. et al. Wnt/beta-catenin pathway mediates (-)-Epigallocatechin-3-gallate (EGCG) inhibition of lung cancer stem cells. *Biochem. Biophys. Res. Commun.* **482**, 15–21 (2017). 27836540 10.1016/j.bbrc.2016.11.038

[328] Chen, Y. et al. (-)-Epigallocatechin-3-gallate inhibits colorectal cancer stem cells by suppressing Wnt/beta-catenin pathway. *Nutrients* **9**, 572 (2017). 10.3390/nu9060572 PMC5490551 28587207

[329] Yang, C., Du, W. & Yang, D. Inhibition of green tea polyphenol EGCG((-)-epigallocatechin-3-gallate) on the proliferation of gastric cancer cells by suppressing canonical wnt/beta-catenin signalling pathway. *Int. J. Food Sci. Nutr.* **67**, 818–827 (2016). 27338284 10.1080/09637486.2016.1198892

[330] Lu, Y., Wei, C. & Xi, Z. Curcumin suppresses proliferation and invasion in non-small cell lung cancer by modulation of MTA1-mediated Wnt/beta-catenin pathway. *Vitr. Cell Dev. Biol. Anim.* **50**, 840–850 (2014). 10.1007/s11626-014-9779-5 24938356

[331] Zhu, J. Y. et al. Curcumin suppresses lung cancer stem cells via inhibiting Wnt/beta-catenin and sonic hedgehog pathways. *Phytother. Res.* **31**, 680–688 (2017). 28198062 10.1002/ptr.5791

[332] Shao, J. et al. LincROR mediates the suppressive effects of curcumin on hepatocellular carcinoma through inactivating Wnt/beta-catenin signaling. *Front. Pharm.* **11**, 847 (2020). 10.3389/fphar.2020.00847 PMC7351502 32714183

[333] Li, H. et al. SPAG5, the upstream protein of Wnt and the target of curcumin, inhibits hepatocellular carcinoma. *Oncol. Rep.* **50**, 1–10 (2023). 10.3892/or.2023.8609 PMC10433440 37539742

[334] Zhang, Z. et al. Curcumin inhibits tumor epithelial-mesenchymal transition by downregulating the Wnt signaling pathway and upregulating NKD2 expression in colon cancer cells. *Oncol. Rep.* **35**, 2615–2623 (2016). 26985708 10.3892/or.2016.4669 PMC4811403

[335] Dou, H. et al. Curcumin suppresses the colon cancer proliferation by inhibiting Wnt/beta-catenin pathways via miR-130a. *Front. Pharm.* **8**, 877 (2017). 10.3389/fphar.2017.00877 PMC5705620 29225578

[336] Hu, P. et al. Both glypican-3/Wnt/beta-catenin signaling pathway and autophagy contributed to the inhibitory effect of curcumin on hepatocellular carcinoma. *Dig. Liver Dis.* **51**, 120–126 (2019). 30001951 10.1016/j.dld.2018.06.012

[337] Zheng, R., Deng, Q., Liu, Y. & Zhao, P. Curcumin inhibits gastric carcinoma cell growth and induces apoptosis by suppressing the Wnt/beta-catenin signaling pathway. *Med. Sci. Monit.* **23**, 163–171 (2017). 28077837 10.12659/MSM.902711 PMC5248567

[338] Liang, Z. et al. Curcumin reversed chronic tobacco smoke exposure induced urocystic EMT and acquisition of cancer stem cells properties via Wnt/beta-catenin. *Cell Death Dis.* **8**, e3066 (2017). 28981096 10.1038/cddis.2017.452 PMC5680574

[339] Liu, H., Zhang, X., Fang, C. & Li, S. Resveratrol induces the growth inhibition of CDX-deficient gastric cancer cells using CDX2 and RUNX3 via the beta-catenin/TCF4 signaling pathway. *Transl. Oncol.* **35**, 101727 (2023). 37354639 10.1016/j.tranon.2023.101727 PMC10320604

[340] Li, S. Y., Shi, C. J., Fu, W. M. & Zhang, J. F. Berberine inhibits tumour growth in vivo and in vitro through suppressing the lincROR-Wnt/beta-catenin regulatory axis in colorectal cancer. *J. Pharm. Pharm.* **75**, 129–138 (2023). 10.1093/jpp/rgac067 36130331

[341] de Almeida, G. C. et al. Piperine suppresses the Wnt/beta-catenin pathway and has anti-cancer effects on colorectal cancer cells. *Sci. Rep.* **10**, 11681 (2020). 32669593 10.1038/s41598-020-68574-2 PMC7363889

[342] Tong, Y. et al. Artemisinin and its derivatives can significantly inhibit lung tumorigenesis and tumor metastasis through Wnt/beta-catenin signaling. *Oncotarget* **7**, 31413–31428 (2016). 27119499 10.18632/oncotarget.8920 PMC5058767

[343] Mandal, S. et al. Inhibition of breast cancer stem-like cells by a triterpenoid, ursolic acid, via activation of Wnt antagonist, sFRP4 and suppression of miRNA-499a-5p. *Life Sci.* **265**, 118854 (2021). 33278391 10.1016/j.lfs.2020.118854

[344] Li, L. et al. Andrographolide suppresses breast cancer progression by modulating tumor-associated macrophage polarization through the Wnt/beta-catenin pathway. *Phytother. Res.* **36**, 4587–4603 (2022). 35916377 10.1002/ptr.7578 PMC10086840

[345] Baud, V. & Karin, M. Is NF-kappaB a good target for cancer therapy? Hopes and pitfalls. *Nat. Rev. Drug Discov.* **8**, 33–40 (2009). 19116625 10.1038/nrd2781 PMC2729321

[346] Zhang, Q., Lenardo, M. J. & Baltimore, D. 30 Years of NF-kappaB: a blossoming of relevance to human pathobiology. *Cell* **168**, 37–57 (2017). 28086098 10.1016/j.cell.2016.12.012 PMC5268070

[347] Sun, S. C. The non-canonical NF-kappaB pathway in immunity and inflammation. *Nat. Rev. Immunol.* **17**, 545–558 (2017). 28580957 10.1038/nri.2017.52 PMC5753586

[348] Morgan, D. et al. Pharmacological significance of the non-canonical NF-kappaB pathway in tumorigenesis. *Biochim. Biophys. Acta Rev. Cancer* **1874**, 188449 (2020). 33058996 10.1016/j.bbcan.2020.188449

[349] Taniguchi, K. & Karin, M. NF-kappaB, inflammation, immunity and cancer: coming of age. *Nat. Rev. Immunol.* **18**, 309–324 (2018). 29379212 10.1038/nri.2017.142

[350] Pikarsky, E. et al. NF-kappaB functions as a tumour promoter in inflammation-associated cancer. *Nature* **431**, 461–466 (2004). 15329734 10.1038/nature02924

[351] Panahi, Y. et al. Molecular mechanisms of curcumins suppressing effects on tumorigenesis, angiogenesis and metastasis, focusing on NF-kappaB pathway. *Cytokine Growth Factor Rev.* **28**, 21–29 (2016). 26774676 10.1016/j.cytogfr.2015.12.004

[352] Capece, D. et al. NF-kappaB: blending metabolism, immunity, and inflammation. *Trends Immunol.* **43**, 757–775 (2022). 35965153 10.1016/j.it.2022.07.004

[353] Zhang, W. et al. Chemoprevention by quercetin of oral squamous cell carcinoma by suppression of the NF-kappaB signaling pathway in DMBA-treated hamsters. *Anticancer Res.* **37**, 4041–4049 (2017). 28739686 10.21873/anticanres.11789

[354] Kielbik, M., Przygodzka, P., Szulc-Kielbik, I. & Klink, M. Snail transcription factors as key regulators of chemoresistance, stemness and metastasis of ovarian cancer cells. *Biochim. Biophys. Acta Rev. Cancer* **1878**, 189003 (2023). 37863122 10.1016/j.bbcan.2023.189003

[355] Tong, J. et al. Apigenin inhibits epithelial-mesenchymal transition of human colon cancer cells through NF-kappaB/Snail signaling pathway. *Biosci. Rep.* **39**, BSR20190452 (2019). 10.1042/BSR20190452 PMC6522743 30967496

[356] Qin, Y. et al. Apigenin inhibits NF-kappaB and snail signaling, EMT and metastasis in human hepatocellular carcinoma. *Oncotarget* **7**, 41421–41431 (2016). 27203387 10.18632/oncotarget.9404 PMC5173069

[357] Wu, D. G. et al. Apigenin potentiates the growth inhibitory effects by IKK-beta-mediated NF-kappaB activation in pancreatic cancer cells. *Toxicol. Lett.* **224**, 157–164 (2014). 24148603 10.1016/j.toxlet.2013.10.007

[358] Luo, K. W. et al. EGCG inhibited bladder cancer SW780 cell proliferation and migration both in vitro and in vivo via down-regulation of NF-kappaB and MMP-9. *J. Nutr. Biochem.* **41**, 56–64 (2017). 28040581 10.1016/j.jnutbio.2016.12.004

[359] Sah, D. K. et al. (-)-Epigallocatechin-3-gallate prevents IL-1beta-induced uPAR expression and invasiveness via the suppression of NF-kappaB and AP-1 in human bladder cancer cells. *Int. J. Mol. Sci.* **23**, 14008 (2022). 10.3390/ijms232214008 PMC9697952 36430487

[360] Sidaway, P. Bladder cancer: uPAR expression indicates worse prognosis of urothelial carcinoma. *Nat. Rev. Urol.* **12**, 120 (2015). 25644167 10.1038/nrurol.2015.5

[361] Li, Y. J. et al. (-)-Epigallocatechin-3-gallate inhibits nasopharyngeal cancer stem cell self-renewal and migration and reverses the epithelial-mesenchymal transition via NF-kappaB p65 inactivation. *Tumour Biol.* **36**, 2747–2761 (2015). 25487615 10.1007/s13277-014-2899-4

[362] Marquardt, J. U. et al. Curcumin effectively inhibits oncogenic NF-kappaB signaling and restrains stemness features in liver cancer. *J. Hepatol.* **63**, 661–669 (2015). 25937435 10.1016/j.jhep.2015.04.018 PMC4543531

[363] Schwertheim, S. et al. Curcumin induces G2/M arrest, apoptosis, NF-kappaB inhibition, and expression of differentiation genes in thyroid carcinoma cells. *J. Cancer Res. Clin. Oncol.* **143**, 1143–1154 (2017). 28265769 10.1007/s00432-017-2380-z PMC11819374

[364] Arumugam, A. et al. Silencing growth hormone receptor inhibits estrogen receptor negative breast cancer through ATP-binding cassette sub-family G member 2. *Exp. Mol. Med.* **51**, 1–13 (2019). 30617282 10.1038/s12276-018-0197-8 PMC6323053

[365] Zhang, W. et al. Autocrine/paracrine human growth hormone-stimulated microRNA 96-182-183 cluster promotes epithelial-mesenchymal transition and invasion in breast cancer. *J. Biol. Chem.* **290**, 13812–13829 (2015). 25873390 10.1074/jbc.M115.653261 PMC4447958

[366] Coker-Gurkan, A. et al. Curcumin inhibits autocrine growth hormone-mediated invasion and metastasis by targeting NF-kappaB signaling and polyamine metabolism in breast cancer cells. *Amino Acids* **50**, 1045–1069 (2018). 29770869 10.1007/s00726-018-2581-z

[367] Coker-Gurkan, A. et al. Curcumin prevented human autocrine growth hormone (GH) signaling mediated NF-kappaB activation and miR-183-96-182 cluster stimulated epithelial mesenchymal transition in T47D breast cancer cells. *Mol. Biol. Rep.* **46**, 355–369 (2019). 30467667 10.1007/s11033-018-4479-y

[368] Huang, F. et al. Curcumin inhibits gastric cancer-derived mesenchymal stem cells mediated angiogenesis by regulating NF-kappaB/VEGF signaling. *Am. J. Transl. Res.* **9**, 5538–5547 (2017). 29312505 PMC5752903

[369] Cao, L. et al. Curcumin inhibits H2O2-induced invasion and migration of human pancreatic cancer via suppression of the ERK/NF-kappaB pathway. *Oncol. Rep.* **36**, 2245–2251 (2016). 27572503 10.3892/or.2016.5044

[370] Tsai, J. R. et al. Curcumin inhibits non-small cell lung cancer cells metastasis through the adiponectin/NF-kappab/MMPs signaling pathway. *PLoS ONE* **10**, e0144462 (2015). 26656720 10.1371/journal.pone.0144462 PMC4675518

[371] Tong, W., Wang, Q., Sun, D. & Suo, J. Curcumin suppresses colon cancer cell invasion via AMPK-induced inhibition of NF-kappaB, uPA activator and MMP9. *Oncol. Lett.* **12**, 4139–4146 (2016). 27895783 10.3892/ol.2016.5148 PMC5104244

[372] Tino, A. B., Chitcholtan, K., Sykes, P. H. & Garrill, A. Resveratrol and acetyl-resveratrol modulate activity of VEGF and IL-8 in ovarian cancer cell aggregates via attenuation of the NF-kappaB protein. *J. Ovarian Res.* **9**, 84 (2016). 27906095 10.1186/s13048-016-0293-0 PMC5134119

[373] Li, M. et al. Induction of apoptosis by berberine in hepatocellular carcinoma HepG2 cells via downregulation of NF-kappaB. *Oncol. Res.* **25**, 233–239 (2017). 28277195 10.3727/096504016X14742891049073 PMC7840840

[374] Liu, J. et al. Homoharringtonine attenuates dextran sulfate sodium-induced colitis by inhibiting NF-kappaB signaling. *Mediators Inflamm.* **2022**, 3441357 (2022). 36211988 10.1155/2022/3441357 PMC9536985

[375] Chen, X. J. et al. Homoharringtonine deregulates MYC transcriptional expression by directly binding NF-kappaB repressing factor. *Proc. Natl. Acad. Sci. USA* **116**, 2220–2225 (2019). 30659143 10.1073/pnas.1818539116 PMC6369765

[376] Zhang, R. et al. Andrographolide suppresses proliferation of human colon cancer SW620 cells through the TLR4/NF-kappaB/MMP-9 signaling pathway. *Oncol. Lett.* **14**, 4305–4310 (2017). 28943944 10.3892/ol.2017.6669 PMC5604146

[377] Yuan, M., Meng, W., Liao, W. & Lian, S. Andrographolide antagonizes TNF-alpha-induced IL-8 via inhibition of NADPH oxidase/ROS/NF-kappaB and Src/MAPKs/AP-1 axis in human colorectal cancer HCT116 cells. *J. Agric. Food Chem.* **66**, 5139–5148 (2018). 29672044 10.1021/acs.jafc.8b00810

[378] Li, J. et al. Andrographolide suppresses the growth and metastasis of luminal-like breast cancer by inhibiting the NF-kappaB/miR-21-5p/PDCD4 signaling pathway. *Front. Cell Dev. Biol.* **9**, 643525 (2021). 34249905 10.3389/fcell.2021.643525 PMC8261247

[379] Li, J., Dai, C. & Shen, L. Ursolic acid inhibits epithelial-mesenchymal transition through the Axl/NF-kappaB pathway in gastric cancer cells. *Evid. Based Complement Altern. Med.* **2019**, 2474805 (2019). 10.1155/2019/2474805 PMC6590617 31281396

[380] Su, T. et al. Artemisinin and its derivatives prevent *Helicobacter pylori*-induced gastric carcinogenesis via inhibition of NF-kappaB signaling. *Phytomedicine* **63**, 152968 (2019). 31280140 10.1016/j.phymed.2019.152968

[381] Hu, X. et al. The JAK/STAT signaling pathway: from bench to clinic. *Signal Transduct. Target Ther.* **6**, 402 (2021). 34824210 10.1038/s41392-021-00791-1 PMC8617206

[382] Salas, A. et al. JAK-STAT pathway targeting for the treatment of inflammatory bowel disease. *Nat. Rev. Gastroenterol. Hepatol.* **17**, 323–337 (2020). 32203403 10.1038/s41575-020-0273-0

[383] Villarino, A. V., Kanno, Y. & O’Shea, J. J. Mechanisms and consequences of Jak-STAT signaling in the immune system. *Nat. Immunol.* **18**, 374–384 (2017). 28323260 10.1038/ni.3691 PMC11565648

[384] Lv, Y. et al. The JAK-STAT pathway: from structural biology to cytokine engineering. *Signal Transduct. Target Ther.* **9**, 221 (2024). 39169031 10.1038/s41392-024-01934-w PMC11339341

[385] Bose, S. et al. Targeting the JAK/STAT signaling pathway using phytocompounds for cancer prevention and therapy. *Cells* **9**, 1451 (2020). 10.3390/cells9061451 PMC7348822 32545187

[386] Garg, M. et al. The pleiotropic role of transcription factor STAT3 in oncogenesis and its targeting through natural products for cancer prevention and therapy. *Med. Res. Rev.* **41**, 1291–1336 (2020). 10.1002/med.21761 33289118

[387] Soendergaard, C., Bergenheim, F. H., Bjerrum, J. T. & Nielsen, O. H. Targeting JAK-STAT signal transduction in IBD. *Pharm. Ther.* **192**, 100–111 (2018). 10.1016/j.pharmthera.2018.07.003 30048708

[388] Fortelny, N. et al. JAK-STAT signaling maintains homeostasis in T cells and macrophages. *Nat. Immunol.* **25**, 847–859 (2024). 38658806 10.1038/s41590-024-01804-1 PMC11065702

[389] Groner, B. & von Manstein, V. Jak Stat signaling and cancer: opportunities, benefits and side effects of targeted inhibition. *Mol. Cell Endocrinol.* **451**, 1–14 (2017). 28576744 10.1016/j.mce.2017.05.033

[390] Igbe, I. et al. Dietary quercetin potentiates the antiproliferative effect of interferon-alpha in hepatocellular carcinoma cells through activation of JAK/STAT pathway signaling by inhibition of SHP2 phosphatase. *Oncotarget* **8**, 113734–113748 (2017). 29371942 10.18632/oncotarget.22556 PMC5768359

[391] Wu, L. et al. Quercetin shows anti-tumor effect in hepatocellular carcinoma LM3 cells by abrogating JAK2/STAT3 signaling pathway. *Cancer Med.* **8**, 4806–4820 (2019). 31273958 10.1002/cam4.2388 PMC6712453

[392] Seo, H. S. et al. Induction of caspase-dependent apoptosis by apigenin by inhibiting STAT3 signaling in HER2-overexpressing MDA-MB-453 breast cancer cells. *Anticancer Res.* **34**, 2869–2882 (2014). 24922650

[393] Seo, H. S. et al. Induction of caspase-dependent extrinsic apoptosis by apigenin through inhibition of signal transducer and activator of transcription 3 (STAT3) signalling in HER2-overexpressing BT-474 breast cancer cells. *Biosci. Rep.* **35**, e00276 (2015). 10.1042/BSR20150165 PMC4708008 26500281

[394] Rodriguez Torres, S. et al. Epigallocatechin-3-gallate prevents the acquisition of a cancer stem cell phenotype in ovarian cancer tumorspheres through the inhibition of Src/JAK/STAT3 signaling. *Biomedicines* **11**, 1000 (2023). 10.3390/biomedicines11041000 PMC10135615 37189618

[395] Liu, Y. et al. The natural polyphenol curcumin induces apoptosis by suppressing STAT3 signaling in esophageal squamous cell carcinoma. *J. Exp. Clin. Cancer Res.* **37**, 303 (2018). 30518397 10.1186/s13046-018-0959-0 PMC6280482

[396] Zhao, H. M. et al. Curcumin suppressed activation of dendritic cells via JAK/STAT/SOCS signal in mice with experimental colitis. *Front. Pharm.* **7**, 455 (2016). 10.3389/fphar.2016.00455 PMC5122716 27932984

[397] Hu, Y., Zhou, N. & Zhu, Q. Curcumin inhibits proliferation and invasion of papillary thyroid carcinoma cells by inhibiting the JAK2/STAT3 pathway. *J. BUON* **26**, 1635–1641 (2021). 34565029

[398] Khan, A. Q. et al. Curcumin-mediated apoptotic cell death in papillary thyroid cancer and cancer stem-like cells through targeting of the JAK/STAT3 signaling pathway. *Int. J. Mol. Sci*. **21**, 438 (2020). 10.3390/ijms21020438 PMC7014270 31936675

[399] Hu, A. et al. Curcumin suppresses invasiveness and vasculogenic mimicry of squamous cell carcinoma of the larynx through the inhibition of JAK-2/STAT-3 signaling pathway. *Am. J. Cancer Res.* **5**, 278–288 (2015). 25628937 PMC4300723

[400] Sun, Y. et al. Curcumin inhibits the proliferation and invasion of MG-63 cells through inactivation of the p-JAK2/p-STAT3 pathway. *OncoTargets Ther.* **12**, 2011–2021 (2019). 10.2147/OTT.S172909 PMC6421868 30936718

[401] Kim, M. J., Park, K. S., Kim, K. T. & Gil, E. Y. The inhibitory effect of curcumin via fascin suppression through JAK/STAT3 pathway on metastasis and recurrence of ovary cancer cells. *BMC Women’s Health* **20**, 256 (2020). 33213437 10.1186/s12905-020-01122-2 PMC7678137

[402] Yaqin, Z. et al. Resveratrol alleviates inflammatory bowel disease by inhibiting JAK2/STAT3 pathway activity via the reduction of O-GlcNAcylation of STAT3 in intestinal epithelial cells. *Toxicol. Appl. Pharm.* **484**, 116882 (2024). 10.1016/j.taap.2024.116882 38437956

[403] Zhang, C. et al. Resveratrol ameliorates glioblastoma inflammatory response by reducing NLRP3 inflammasome activation through inhibition of the JAK2/STAT3 pathway. *J. Cancer Res. Clin. Oncol.* **150**, 168 (2024). 38546908 10.1007/s00432-024-05625-5 PMC10978631

[404] Xu, M. et al. Berberine inhibits gastric cancer development and progression by regulating the JAK2/STAT3 pathway and downregulating IL-6. *Life Sci.* **290**, 120266 (2022). 34968467 10.1016/j.lfs.2021.120266

[405] Cao, W. et al. Homoharringtonine induces apoptosis and inhibits STAT3 via IL-6/JAK1/STAT3 signal pathway in Gefitinib-resistant lung cancer cells. *Sci. Rep.* **5**, 8477 (2015). 26166037 10.1038/srep08477 PMC4499885

[406] Zheng, Y. & Pan, D. The Hippo signaling pathway in development and disease. *Dev. Cell* **50**, 264–282 (2019). 31386861 10.1016/j.devcel.2019.06.003 PMC6748048

[407] Yu, F. X., Zhao, B. & Guan, K. L. Hippo pathway in organ size control, tissue homeostasis, and cancer. *Cell* **163**, 811–828 (2015). 26544935 10.1016/j.cell.2015.10.044 PMC4638384

[408] Mohajan, S. et al. Hippo pathway: regulation, deregulation and potential therapeutic targets in cancer. *Cancer Lett.* **507**, 112–123 (2021). 33737002 10.1016/j.canlet.2021.03.006 PMC10370464

[409] Hong, L. et al. Role of Hippo signaling in regulating immunity. *Cell Mol. Immunol.* **15**, 1003–1009 (2018). 29568120 10.1038/s41423-018-0007-1 PMC6269503

[410] Zhou, Y. et al. Emerging roles of Hippo signaling in inflammation and YAP-driven tumor immunity. *Cancer Lett.* **426**, 73–79 (2018). 29654891 10.1016/j.canlet.2018.04.004

[411] Zhang, Y., Zhang, H. & Zhao, B. Hippo signaling in the immune system. *Trends Biochem. Sci.* **43**, 77–80 (2018). 29249569 10.1016/j.tibs.2017.11.009

[412] Janse van Rensburg, H. J. et al. The hippo pathway component TAZ promotes immune evasion in human cancer through PD-L1. *Cancer Res.* **78**, 1457–1470 (2018). 29339539 10.1158/0008-5472.CAN-17-3139

[413] Yan, R. et al. Inhibition of DCLK1 down-regulates PD-L1 expression through Hippo pathway in human pancreatic cancer. *Life Sci.* **241**, 117150 (2020). 31837335 10.1016/j.lfs.2019.117150

[414] Liu, C., Song, Y., Li, D. & Wang, B. Regulation of the tumor immune microenvironment by the Hippo pathway: implications for cancer immunotherapy. *Int Immunopharmacol.* **122**, 110586 (2023). 37393838 10.1016/j.intimp.2023.110586

[415] Liedtke, C. et al. Response to neoadjuvant therapy and long-term survival in patients with triple-negative breast cancer. *J. Clin. Oncol.* **41**, 1809–1815 (2023). 10.1200/JCO.2007.14.4147 18250347

[416] Li, Y. W. et al. Apigenin suppresses the stem cell-like properties of triple-negative breast cancer cells by inhibiting YAP/TAZ activity. *Cell Death Discov.* **4**, 105 (2018). 30479839 10.1038/s41420-018-0124-8 PMC6244166

[417] Zeng, J. et al. Apigenin regulates the migration, invasion, and autophagy of hepatocellular carcinoma cells by downregulating YAP. *Neoplasma* **69**, 292–302 (2022). 35014535 10.4149/neo_2021_210615N798

[418] Li, A. et al. Epigallocatechin-3-gallate affects the proliferation, apoptosis, migration and invasion of tongue squamous cell carcinoma through the hippo-TAZ signaling pathway. *Int. J. Mol. Med.* **42**, 2615–2627 (2018). 30106116 10.3892/ijmm.2018.3818 PMC6192764

[419] Wu, Y. et al. KLF5 promotes tumor progression and parp inhibitor resistance in ovarian cancer. *Adv. Sci.* **10**, e2304638 (2023). 10.1002/advs.202304638 PMC10625120 37702443

[420] Jiang, D. et al. YB-1 is a positive regulator of KLF5 transcription factor in basal-like breast cancer. *Cell Death Differ.* **29**, 1283–1295 (2022). 35022570 10.1038/s41418-021-00920-x PMC9177637

[421] Zhang, B. et al. Acetylation of KLF5 maintains EMT and tumorigenicity to cause chemoresistant bone metastasis in prostate cancer. *Nat. Commun.* **12**, 1714 (2021). 33731701 10.1038/s41467-021-21976-w PMC7969754

[422] Gao, Y. et al. Curcumin promotes KLF5 proteasome degradation through downregulating YAP/TAZ in bladder cancer cells. *Int. J. Mol. Sci.* **15**, 15173–15187 (2014). 25170806 10.3390/ijms150915173 PMC4200832

[423] Zhou, X. et al. Antitumor activity of curcumin is involved in down-regulation of YAP/TAZ expression in pancreatic cancer cells. *Oncotarget* **7**, 79076–79088 (2016). 27738325 10.18632/oncotarget.12596 PMC5346699

[424] Zhu, J. et al. Curcumin induces autophagy via inhibition of Yes-associated protein (YAP) in human colon cancer cells. *Med. Sci. Monit.* **24**, 7035–7042 (2018). 30281585 10.12659/MSM.910650 PMC6354647

[425] Zheng, Y. et al. Curcumin suppresses the stemness of non-small cell lung cancer cells via promoting the nuclear-cytoplasm translocation of TAZ. *Environ. Toxicol.* **36**, 1135–1142 (2021). 33539684 10.1002/tox.23112

[426] Thiele, M. et al. Screening for liver fibrosis: lessons from colorectal and lung cancer screening. *Nat. Rev. Gastroenterol. Hepatol.* **21**, 517–527 (2024). 38480849 10.1038/s41575-024-00907-2

[427] Li, C., Zhang, R., Zhan, Y. & Zheng, J. Resveratrol inhibits hepatic stellate cell activation via the hippo pathway. *Mediators Inflamm.* **2021**, 3399357 (2021). 34690551 10.1155/2021/3399357 PMC8528611

[428] Xu, G. et al. Resveratrol inhibits the tumorigenesis of follicular thyroid cancer via ST6GAL2-regulated activation of the hippo signaling pathway. *Mol. Ther. Oncolytics* **16**, 124–133 (2020). 32055676 10.1016/j.omto.2019.12.010 PMC7005482

[429] Qin, X. et al. Resveratrol inhibits proliferation and induces apoptosis via the Hippo/YAP pathway in human colon cancer cells. *Biochem Biophys. Res. Commun.* **636**, 197–204 (2022). 36335870 10.1016/j.bbrc.2022.10.077

[430] Kim, Y. N. et al. Resveratrol suppresses breast cancer cell invasion by inactivating a RhoA/YAP signaling axis. *Exp. Mol. Med.* **49**, e296 (2017). 28232662 10.1038/emm.2016.151 PMC5336560

[431] Deng, L. et al. Resveratrol inhibits TGF-beta1-induced EMT in gastric cancer cells through Hippo-YAP signaling pathway. *Clin. Transl. Oncol.* **24**, 2210–2221 (2022). 35842894 10.1007/s12094-022-02882-z

[432] Wang, H. et al. Homoharringtonine exerts anti-tumor effects in hepatocellular carcinoma through activation of the hippo pathway. *Front. Pharm.* **12**, 592071 (2021). 10.3389/fphar.2021.592071 PMC7943857 33716735

[433] Kim, S. H. et al. Activating hippo pathway via rassf1 by ursolic acid suppresses the tumorigenesis of gastric cancer. *Int. J. Mol. Sci*. **20**, 4709 (2019). 10.3390/ijms20194709 PMC6801984 31547587

[434] Li, Y. et al. Artemisinin suppresses hepatocellular carcinoma cell growth, migration and invasion by targeting cellular bioenergetics and Hippo-YAP signaling. *Arch. Toxicol.* **93**, 3367–3383 (2019). 31563988 10.1007/s00204-019-02579-3

[435] Hanna, A. & Shevde, L. A. Hedgehog signaling: modulation of cancer properies and tumor mircroenvironment. *Mol. Cancer* **15**, 24 (2016). 26988232 10.1186/s12943-016-0509-3 PMC4797362

[436] Salaritabar, A. et al. Targeting Hedgehog signaling pathway: paving the road for cancer therapy. *Pharm. Res* **141**, 466–480 (2019). 10.1016/j.phrs.2019.01.014 30639373

[437] Takebe, N. et al. Targeting Notch, Hedgehog, and Wnt pathways in cancer stem cells: clinical update. *Nat. Rev. Clin. Oncol.* **12**, 445–464 (2015). 25850553 10.1038/nrclinonc.2015.61 PMC4520755

[438] Jiang, J. Hedgehog signaling mechanism and role in cancer. *Semin Cancer Biol.* **85**, 107–122 (2022). 33836254 10.1016/j.semcancer.2021.04.003 PMC8492792

[439] Zhang, Q. et al. Genistein inhibits nasopharyngeal cancer stem cells through sonic hedgehog signaling. *Phytother. Res.* **33**, 2783–2791 (2019). 31342620 10.1002/ptr.6464

[440] Li, E. et al. Sonic hedgehog pathway mediates genistein inhibition of renal cancer stem cells. *Oncol. Lett.* **18**, 3081–3091 (2019). 31452785 10.3892/ol.2019.10657 PMC6704282

[441] Yu, D. et al. Genistein attenuates cancer stem cell characteristics in gastric cancer through the downregulation of Gli1. *Oncol. Rep.* **31**, 673–678 (2014). 24297371 10.3892/or.2013.2893

[442] Sun, X. et al. (-)-Epigallocatechin-3-gallate inhibits bladder cancer stem cells via suppression of sonic hedgehog pathway. *Oncol. Rep.* **42**, 425–435 (2019). 31180522 10.3892/or.2019.7170

[443] Wang, D. et al. Curcumin inhibits bladder cancer stem cells by suppressing Sonic Hedgehog pathway. *Biochem. Biophys. Res. Commun.* **493**, 521–527 (2017). 28870814 10.1016/j.bbrc.2017.08.158

[444] Li, M. et al. Curcumin inhibits the invasion and metastasis of triple negative breast cancer via Hedgehog/Gli1 signaling pathway. *J. Ethnopharmacol.* **283**, 114689 (2022). 34592340 10.1016/j.jep.2021.114689

[445] Cao, L. et al. Curcumin inhibits hypoxia-induced epithelial-mesenchymal transition in pancreatic cancer cells via suppression of the hedgehog signaling pathway. *Oncol. Rep.* **35**, 3728–3734 (2016). 27035865 10.3892/or.2016.4709

[446] Xu, Q. H. et al. Resveratrol counteracts hypoxia-induced gastric cancer invasion and EMT through Hedgehog pathway suppression. *Anticancer Agents Med. Chem.* **20**, 1105–1114 (2020). 32238142 10.2174/1871520620666200402080034

[447] Gao, Q., Yuan, Y., Gan, H. Z. & Peng, Q. Resveratrol inhibits the hedgehog signaling pathway and epithelial-mesenchymal transition and suppresses gastric cancer invasion and metastasis. *Oncol. Lett.* **9**, 2381–2387 (2015). 26137075 10.3892/ol.2015.2988 PMC4467343

[448] Qin, Y. et al. Effect of resveratrol on proliferation and apoptosis of human pancreatic cancer MIA PaCa-2 cells may involve inhibition of the Hedgehog signaling pathway. *Mol. Med. Rep.* **10**, 2563–2567 (2014). 25190613 10.3892/mmr.2014.2511

[449] Li, W. et al. Resveratrol inhibits hypoxia-driven ROS-induced invasive and migratory ability of pancreatic cancer cells via suppression of the Hedgehog signaling pathway. *Oncol. Rep.* **35**, 1718–1726 (2016). 26707376 10.3892/or.2015.4504

[450] Ferraresi, A. et al. Resveratrol contrasts LPA-induced ovarian cancer cell migration and platinum resistance by rescuing hedgehog-mediated autophagy. *Cells* **10**, 3213(2021). 10.3390/cells10113213 PMC8625920 34831435

[451] Jiang, J. et al. Resveratrol induces apoptosis, suppresses migration, and invasion of cervical cancer cells by inhibiting the hedgehog signaling pathway. *Biomed. Res. Int.* **2022**, 8453011 (2022). 36246980 10.1155/2022/8453011 PMC9568329

[452] Du, Z. et al. The hedgehog/Gli-1 signaling pathways is involved in the inhibitory effect of resveratrol on human colorectal cancer HCT116 cells. *Iran. J. Basic Med. Sci.* **19**, 1171–1176 (2016). 27917272 PMC5126217

[453] Sun, H. et al. Resveratrol inhibition of renal cancer stem cell characteristics and modulation of the sonic Hedgehog pathway. *Nutr. Cancer* **73**, 1157–1167 (2021). 32586140 10.1080/01635581.2020.1784966

[454] Wang, J. et al. Berberine, a natural compound, suppresses Hedgehog signaling pathway activity and cancer growth. *BMC Cancer* **15**, 595 (2015). 26296751 10.1186/s12885-015-1596-z PMC4546096

[455] Sun, Q. et al. Berberine is a suppressor of Hedgehog signaling cascade in colorectal cancer. *Phytomedicine* **114**, 154792 (2023). 37028248 10.1016/j.phymed.2023.154792

[456] Elhefnawy, E. A. et al. Genistein and/or sulfasalazine ameliorate acetic acid-induced ulcerative colitis in rats via modulating INF-gamma/JAK1/STAT1/IRF-1, TLR-4/NF-kappaB/IL-6, and JAK2/STAT3/COX-2 crosstalk. *Biochem Pharm.* **214**, 115673 (2023). 37414101 10.1016/j.bcp.2023.115673

[457] Zhang, J. et al. Quercetin inhibits chronic stress-mediated progression of triple-negative breast cancer by blocking beta(2)-AR/ERK1/2 pathway. *Biomed. Pharmacother.* **177**, 116985 (2024). 38901200 10.1016/j.biopha.2024.116985

[458] Xia, Y. et al. Apigenin suppresses the IL-1beta-induced expression of the urokinase-type plasminogen activator receptor by inhibiting MAPK-mediated AP-1 and NF-kappaB signaling in human bladder cancer T24 cells. *J. Agric. Food Chem.* **66**, 7663–7673 (2018). 29945448 10.1021/acs.jafc.8b02351

[459] Kassouri, C. et al. EGCG Prevents the transcriptional reprogramming of an inflammatory and immune-suppressive molecular signature in macrophage-like differentiated human HL60 promyelocytic leukemia cells. *Cancers* **14**, 5065 (2022). 10.3390/cancers14205065 PMC9599716 36291849

[460] Kurzava Kendall, L. et al. Epigenetic effects of resveratrol on oncogenic signaling in breast cancer. *Nutrients* **16**, 699 (2024). 10.3390/nu16050699 PMC10934115 38474826

[461] Li, L. et al. Berberine could inhibit thyroid carcinoma cells by inducing mitochondrial apoptosis, G0/G1 cell cycle arrest and suppressing migration via PI3K-AKT and MAPK signaling pathways. *Biomed. Pharmacother.* **95**, 1225–1231 (2017). 28931215 10.1016/j.biopha.2017.09.010

[462] Hu, Q. et al. Berberine attenuated proliferation, invasion and migration by targeting the AMPK/HNF4alpha/WNT5A pathway in gastric carcinoma. *Front. Pharm.* **9**, 1150 (2018). 10.3389/fphar.2018.01150 PMC6202939 30405404

[463] Cheng, C. C. et al. STAT3 exacerbates survival of cancer stem-like tumorspheres in EGFR-positive colorectal cancers: RNAseq analysis and therapeutic screening. *J. Biomed. Sci.* **25**, 60 (2018). 30068339 10.1186/s12929-018-0456-y PMC6090986

[464] Rehman, M. U. et al. Piperine regulates Nrf-2/Keap-1 signalling and exhibits anticancer effect in experimental colon carcinogenesis in Wistar rats. *Biology* **9**, 302 (2020). 10.3390/biology9090302 PMC7565681 32967203

[465] Luo, J., Hu, Y. L. & Wang, H. Ursolic acid inhibits breast cancer growth by inhibiting proliferation, inducing autophagy and apoptosis, and suppressing inflammatory responses via the PI3K/AKT and NF-kappaB signaling pathways in vitro. *Exp. Ther. Med.* **14**, 3623–3631 (2017). 29042957 10.3892/etm.2017.4965 PMC5639319

[466] Sontheimer-Phelps, A., Hassell, B. A. & Ingber, D. E. Modelling cancer in microfluidic human organs-on-chips. *Nat. Rev. Cancer* **19**, 65–81 (2019). 30647431 10.1038/s41568-018-0104-6

[467] Fujii, M., Clevers, H. & Sato, T. Modeling human digestive diseases with CRISPR-Cas9-modified organoids. *Gastroenterology* **156**, 562–576 (2019). 30476497 10.1053/j.gastro.2018.11.048

[468] Vlachogiannis, G. et al. Patient-derived organoids model treatment response of metastatic gastrointestinal cancers. *Science* **359**, 920–926 (2018). 29472484 10.1126/science.aao2774 PMC6112415

[469] Mircetic, J. et al. CRISPR/Cas9 screen in gastric cancer patient-derived organoids reveals KDM1A-NDRG1 axis as a targetable vulnerability. *Small Methods* **7**, e2201605 (2023). 36908010 10.1002/smtd.202201605

[470] Magre, L. et al. Emerging organoid-immune co-culture models for cancer research: from oncoimmunology to personalized immunotherapies. *J. Immunother. Cancer* **11**, 5 (2023). 10.1136/jitc-2022-006290 PMC10231025 37220953

[471] Makarova, K. S. et al. Evolutionary classification of CRISPR-Cas systems: a burst of class 2 and derived variants. *Nat. Rev. Microbiol.* **18**, 67–83 (2020). 31857715 10.1038/s41579-019-0299-x PMC8905525

[472] Sket, T., Falcomata, C. & Saur, D. Dual recombinase-based mouse models help decipher cancer biology and targets for therapy. *Cancer Res.* **83**, 2279–2282 (2023). 37449355 10.1158/0008-5472.CAN-22-2119 PMC10351565

[473] Deng, L. J. et al. Natural products and their derivatives: promising modulators of tumor immunotherapy. *J. Leukoc. Biol.* **108**, 493–508 (2020). 32678943 10.1002/JLB.3MR0320-444R PMC7496826

[474] Fenis, A. et al. New immune cell engagers for cancer immunotherapy. *Nat. Rev. Immunol.* **24**, 471–486 (2024). 38273127 10.1038/s41577-023-00982-7

[475] He, X. & Xu, C. Immune checkpoint signaling and cancer immunotherapy. *Cell Res.* **30**, 660–669 (2020). 32467592 10.1038/s41422-020-0343-4 PMC7395714

[476] Sun, C., Mezzadra, R. & Schumacher, T. N. Regulation and function of the PD-L1 checkpoint. *Immunity* **48**, 434–452 (2018). 29562194 10.1016/j.immuni.2018.03.014 PMC7116507

[477] Pardoll, D. M. The blockade of immune checkpoints in cancer immunotherapy. *Nat. Rev. Cancer* **12**, 252–264 (2012). 22437870 10.1038/nrc3239 PMC4856023

[478] Escors, D. et al. The intracellular signalosome of PD-L1 in cancer cells. *Signal Transduct. Target Ther.* **3**, 26 (2018). 30275987 10.1038/s41392-018-0022-9 PMC6160488

[479] Akinleye, A. & Rasool, Z. Immune checkpoint inhibitors of PD-L1 as cancer therapeutics. *J. Hematol. Oncol.* **12**, 92 (2019). 31488176 10.1186/s13045-019-0779-5 PMC6729004

[480] Doroshow, D. B. et al. PD-L1 as a biomarker of response to immune-checkpoint inhibitors. *Nat. Rev. Clin. Oncol.* **18**, 345–362 (2021). 33580222 10.1038/s41571-021-00473-5

[481] Keir, M. E., Butte, M. J., Freeman, G. J. & Sharpe, A. H. PD-1 and its ligands in tolerance and immunity. *Annu. Rev. Immunol.* **26**, 677–704 (2008). 18173375 10.1146/annurev.immunol.26.021607.090331 PMC10637733

[482] Ribas, A. Tumor immunotherapy directed at PD-1. *New Engl. J. Med.* **366**, 2517–2519 (2012). 22658126 10.1056/NEJMe1205943

[483] Xu, L. et al. Apigenin suppresses PD-L1 expression in melanoma and host dendritic cells to elicit synergistic therapeutic effects. *J. Exp. Clin. Cancer Res.* **37**, 261 (2018). 30373602 10.1186/s13046-018-0929-6 PMC6206930

[484] Lim, S. O. et al. Deubiquitination and Stabilization of PD-L1 by CSN5. *Cancer Cell* **30**, 925–939 (2016). 27866850 10.1016/j.ccell.2016.10.010 PMC5171205

[485] Verdura, S. et al. Resveratrol targets PD-L1 glycosylation and dimerization to enhance antitumor T-cell immunity. *Aging* **12**, 8–34 (2020). 31901900 10.18632/aging.102646 PMC6977679

[486] Yang, M. et al. Resveratrol induces PD-L1 expression through snail-driven activation of Wnt pathway in lung cancer cells. *J. Cancer Res. Clin. Oncol.* **147**, 1101–1113 (2021). 33471184 10.1007/s00432-021-03510-z PMC7954741

[487] Liu, Y. et al. Fucoxanthin activates apoptosis via inhibition of PI3K/Akt/mTOR pathway and suppresses invasion and migration by restriction of p38-MMP-2/9 pathway in human glioblastoma cells. *Neurochem. Res.* **41**, 2728–2751 (2016). 27394418 10.1007/s11064-016-1989-7

[488] Wang, X. R. et al. Andrographolide suppresses non-small-cell lung cancer progression through induction of autophagy and antitumor immune response. *Pharm. Res.* **179**, 106198 (2022). 10.1016/j.phrs.2022.106198 35367343

[489] Kang, D. Y., Sp, N., Lee, J. M. & Jang, K. J. Antitumor effects of ursolic acid through mediating the inhibition of STAT3/PD-L1 signaling in non-small cell lung cancer cells. *Biomedicines* **9**, 297 (2021). 10.3390/biomedicines9030297 PMC7998465 33805840

[490] Hathcock, K. S. et al. Identification of an alternative CTLA-4 ligand costimulatory for T cell activation. *Science* **262**, 905–907 (1993). 7694361 10.1126/science.7694361

[491] Chambers, C. A., Kuhns, M. S., Egen, J. G. & Allison, J. P. CTLA-4-mediated inhibition in regulation of T cell responses: mechanisms and manipulation in tumor immunotherapy. *Annu. Rev. Immunol.* **19**, 565–594 (2001). 11244047 10.1146/annurev.immunol.19.1.565

[492] Egen, J. G., Kuhns, M. S. & Allison, J. P. CTLA-4: new insights into its biological function and use in tumor immunotherapy. *Nat. Immunol.* **3**, 611–618 (2002). 12087419 10.1038/ni0702-611

[493] Chae, Y. K. et al. Current landscape and future of dual anti-CTLA4 and PD-1/PD-L1 blockade immunotherapy in cancer; lessons learned from clinical trials with melanoma and non-small cell lung cancer (NSCLC). *J. Immunother. Cancer* **6**, 39 (2018). 29769148 10.1186/s40425-018-0349-3 PMC5956851

[494] Takahashi, T. et al. Immunologic self-tolerance maintained by CD25(+)CD4(+) regulatory T cells constitutively expressing cytotoxic T lymphocyte-associated antigen 4. *J. Exp. Med.* **192**, 303–310 (2000). 10899917 10.1084/jem.192.2.303 PMC2193248

[495] Zhao, G. J. et al. Curcumin inhibits suppressive capacity of naturally occurring CD4+CD25+ regulatory T cells in mice in vitro. *Int. Immunopharmacol.* **14**, 99–106 (2012). 22749847 10.1016/j.intimp.2012.06.016

[496] Wang, B. et al. Resveratrol prevents suppression of regulatory T-cell production, oxidative stress, and inflammation of mice prone or resistant to high-fat diet-induced obesity. *Nutr. Res.* **33**, 971–981 (2013). 24176237 10.1016/j.nutres.2013.07.016

[497] Zitvogel, L. et al. Anticancer effects of the microbiome and its products. *Nat. Rev. Microbiol.* **15**, 465–478 (2017). 28529325 10.1038/nrmicro.2017.44

[498] Helmink, B. A. et al. The microbiome, cancer, and cancer therapy. *Nat. Med.* **25**, 377–388 (2019). 30842679 10.1038/s41591-019-0377-7

[499] Singh, A. et al. Microbiome and host crosstalk: a new paradigm to cancer therapy. *Semin. Cancer Biol.* **70**, 71–84 (2021). 32479952 10.1016/j.semcancer.2020.05.014

[500] Pandya, G. et al. A comprehensive review of the multifaceted role of the microbiota in human pancreatic carcinoma. *Semin. Cancer Biol.* **86**, 682–692 (2022). 34051351 10.1016/j.semcancer.2021.05.027

[501] Cullin, N. et al. Microbiome and cancer. *Cancer Cell* **39**, 1317–1341 (2021). 34506740 10.1016/j.ccell.2021.08.006

[502] Sepich-Poore, G. D. et al. The microbiome and human cancer. *Science* **371**, eabc4552 (2021). 10.1126/science.abc4552 PMC8767999 33766858

[503] Gopalakrishnan, V. et al. The influence of the gut microbiome on cancer, immunity, and cancer immunotherapy. *Cancer Cell* **33**, 570–580 (2018). 29634945 10.1016/j.ccell.2018.03.015 PMC6529202

[504] Liou, J. M. et al. Screening and eradication of *Helicobacter pylori* for gastric cancer prevention: the Taipei global consensus. *Gut* **69**, 2093–2112 (2020). 33004546 10.1136/gutjnl-2020-322368

[505] Sugizaki, K. et al. Anti-Helicobacter pylori therapy in localized gastric mucosa-associated lymphoid tissue lymphoma: a prospective, nationwide, multicenter study in Japan. *Helicobacter* **23**, e12474 (2018). 29504247 10.1111/hel.12474 PMC5900897

[506] Wang, N. & Fang, J. Y. Fusobacterium nucleatum, a key pathogenic factor and microbial biomarker for colorectal cancer. *Trends Microbiol.* **31**, 159–172 (2023). 36058786 10.1016/j.tim.2022.08.010

[507] Iftekhar, A. et al. Genomic aberrations after short-term exposure to colibactin-producing *E. coli* transform primary colon epithelial cells. *Nat. Commun.* **12**, 1003 (2021). 33579932 10.1038/s41467-021-21162-y PMC7881031

[508] Zhou, C. B., Zhou, Y. L. & Fang, J. Y. Gut microbiota in cancer immune response and immunotherapy. *Trends Cancer* **7**, 647–660 (2021). 33674230 10.1016/j.trecan.2021.01.010

[509] Bhatt, A. P., Redinbo, M. R. & Bultman, S. J. The role of the microbiome in cancer development and therapy. *CA Cancer J. Clin.* **67**, 326–344 (2017). 28481406 10.3322/caac.21398 PMC5530583

[510] Liu, L. & Shah, K. The potential of the gut microbiome to reshape the cancer therapy paradigm: a review. *JAMA Oncol.* **8**, 1059–1067 (2022). 35482355 10.1001/jamaoncol.2022.0494

[511] Chattopadhyay, I., Nandi, D. & Nag, A. The pint- sized powerhouse: Illuminating the mighty role of the gut microbiome in improving the outcome of anti- cancer therapy. *Semin. Cancer Biol.* **70**, 98–111 (2021). 32739479 10.1016/j.semcancer.2020.07.012

[512] Geller, L. T. et al. Potential role of intratumor bacteria in mediating tumor resistance to the chemotherapeutic drug gemcitabine. *Science* **357**, 1156–1160 (2017). 28912244 10.1126/science.aah5043 PMC5727343

[513] Johnson, D. B. et al. Targeted next generation sequencing identifies markers of response to PD-1 blockade. *Cancer Immunol. Res.* **4**, 959–967 (2016). 27671167 10.1158/2326-6066.CIR-16-0143 PMC5134329

[514] Sivan, A. et al. Commensal Bifidobacterium promotes antitumor immunity and facilitates anti-PD-L1 efficacy. *Science* **350**, 1084–1089 (2015). 26541606 10.1126/science.aac4255 PMC4873287

[515] Zhang, M., Liu, J. & Xia, Q. Role of gut microbiome in cancer immunotherapy: from predictive biomarker to therapeutic target. *Exp. Hematol. Oncol.* **12**, 84 (2023). 37770953 10.1186/s40164-023-00442-x PMC10537950

[516] Manni, A. et al. Complementarity between microbiome and immunity may account for the potentiating effect of quercetin on the antitumor action of cyclophosphamide in a triple-negative breast cancer model. *Pharmaceuticals* **16**, 1422 (2023). 10.3390/ph16101422 PMC10610118 37895893

[517] Fu, R. et al. Apigenin remodels the gut microbiota to ameliorate ulcerative colitis. *Front Nutr.* **9**, 1062961 (2022). 36590200 10.3389/fnut.2022.1062961 PMC9800908

[518] Bian, S., Wan, H., Liao, X. & Wang, W. Inhibitory effects of apigenin on tumor carcinogenesis by altering the gut microbiota. *Mediators Inflamm.* **2020**, 7141970 (2020). 33082711 10.1155/2020/7141970 PMC7559228

[519] McFadden, R. M. et al. The role of curcumin in modulating colonic microbiota during colitis and colon cancer prevention. *Inflamm. Bowel Dis.* **21**, 2483–2494 (2015). 26218141 10.1097/MIB.0000000000000522 PMC4615313

[520] Yu, B. et al. Resveratrol ameliorates DSS-induced ulcerative colitis by acting on mouse gut microbiota. *Inflammopharmacology* **32**, 2023–2033 (2024). 38492181 10.1007/s10787-024-01456-5

[521] Dong, Y. et al. Berberine ameliorates DSS-induced intestinal mucosal barrier dysfunction through microbiota-dependence and Wnt/beta-catenin pathway. *Int. J. Biol. Sci.* **18**, 1381–1397 (2022). 35280677 10.7150/ijbs.65476 PMC8898376

[522] Sun, X. et al. Berberine improves DSS-induced colitis in mice by modulating the fecal-bacteria-related bile acid metabolism. *Biomed. Pharmacother.* **167**, 115430 (2023). 37683590 10.1016/j.biopha.2023.115430

[523] Jing, W. et al. Berberine improves colitis by triggering AhR activation by microbial tryptophan catabolites. *Pharm. Res.* **164**, 105358 (2021). 10.1016/j.phrs.2020.105358 33285228

[524] Sun, Q. et al. Berberine suppresses colorectal cancer by regulation of Hedgehog signaling pathway activity and gut microbiota. *Phytomedicine* **103**, 154227 (2022). 35679795 10.1016/j.phymed.2022.154227

[525] Chen, H. et al. A holistic view of berberine inhibiting intestinal carcinogenesis in conventional mice based on microbiome-metabolomics analysis. *Front. Immunol.* **11**, 588079 (2020). 33072135 10.3389/fimmu.2020.588079 PMC7541814

[526] Sun, Y. et al. Berberine inhibits breast carcinoma proliferation and metastasis under hypoxic microenvironment involving gut microbiota and endogenous metabolites. *Pharm. Res.* **193**, 106817 (2023). 10.1016/j.phrs.2023.106817 37315824

[527] Shou, J. W. & Shaw, P. C. Berberine activates PPARdelta and promotes gut microbiota-derived butyric acid to suppress hepatocellular carcinoma. *Phytomedicine* **115**, 154842 (2023). 37148713 10.1016/j.phymed.2023.154842

[528] Sheng, Q. et al. Ursolic acid regulates intestinal microbiota and inflammatory cell infiltration to prevent ulcerative colitis. *J. Immunol. Res.* **2021**, 6679316 (2021). 34007853 10.1155/2021/6679316 PMC8111854

[529] Chehelgerdi, M. et al. Progressing nanotechnology to improve targeted cancer treatment: overcoming hurdles in its clinical implementation. *Mol. Cancer* **22**, 169 (2023). 37814270 10.1186/s12943-023-01865-0 PMC10561438

[530] Kashyap, D. et al. Natural product-based nanoformulations for cancer therapy: opportunities and challenges. *Semin. Cancer Biol.* **69**, 5–23 (2021). 31421264 10.1016/j.semcancer.2019.08.014

[531] Siddiqui, I. A. & Sanna, V. Impact of nanotechnology on the delivery of natural products for cancer prevention and therapy. *Mol. Nutr. Food Res.* **60**, 1330–1341 (2016). 26935239 10.1002/mnfr.201600035

[532] Khetan, R. et al. Using GPCRs as molecular beacons to target ovarian cancer with nanomedicines. *Cancers* **14**, 2362 (2022). 10.3390/cancers14102362 PMC9140059 35625966

[533] Xu, M. & Li, S. Nano-drug delivery system targeting tumor microenvironment: a prospective strategy for melanoma treatment. *Cancer Lett.* **574**, 216397 (2023). 37730105 10.1016/j.canlet.2023.216397

[534] Large, D. E., Abdelmessih, R. G., Fink, E. A. & Auguste, D. T. Liposome composition in drug delivery design, synthesis, characterization, and clinical application. *Adv. Drug Deliv. Rev.* **176**, 113851 (2021). 34224787 10.1016/j.addr.2021.113851

[535] Chen, J. et al. Recent advances and clinical translation of liposomal delivery systems in cancer therapy. *Eur. J. Pharm. Sci.* **193**, 106688 (2024). 38171420 10.1016/j.ejps.2023.106688

[536] Gu, Z. et al. Effective combination of liposome-targeted chemotherapy and PD-L1 blockade of murine colon cancer. *J. Control Release* **353**, 490–506 (2023). 36460179 10.1016/j.jconrel.2022.11.049

[537] George, T. A. et al. Liposome-encapsulated anthraquinone improves efficacy and safety in triple negative breast cancer. *J. Control Release* **342**, 31–43 (2022). 34896187 10.1016/j.jconrel.2021.12.001

[538] Zhang, Z. et al. Holo-lactoferrin modified liposome for relieving tumor hypoxia and enhancing radiochemotherapy of cancer. *Small* **15**, e1803703 (2019). 30645056 10.1002/smll.201803703

[539] Wang, X. et al. Miriplatin-loaded liposome, as a novel mitophagy inducer, suppresses pancreatic cancer proliferation through blocking POLG and TFAM-mediated mtDNA replication. *Acta Pharm. Sin. B* **13**, 4477–4501 (2023). 37969736 10.1016/j.apsb.2023.07.009 PMC10638513

[540] Bhagya, N. & Chandrashekar, K. R. Liposome encapsulated anticancer drugs on autophagy in cancer cells—current and future perspective. *Int. J. Pharm.* **642**, 123105 (2023). 37279869 10.1016/j.ijpharm.2023.123105

[541] Cho, E. et al. Tumor-targeted liposomes with platycodin D2 promote apoptosis in colorectal cancer. *Mater. Today Bio* **22**, 100745 (2023). 37576871 10.1016/j.mtbio.2023.100745 PMC10415802

[542] Gao, A. et al. Overview of recent advances in liposomal nanoparticle-based cancer immunotherapy. *Acta Pharm. Sin.* **40**, 1129–1137 (2019). 10.1038/s41401-019-0281-1 PMC6786406 31371782

[543] Ma, G. L. & Lin, W. F. Immune checkpoint inhibition mediated with liposomal nanomedicine for cancer therapy. *Mil. Med. Res.* **10**, 20 (2023). 37106400 10.1186/s40779-023-00455-x PMC10142459

[544] Barenholz, Y. Doxil(R)–the first FDA-approved nano-drug: lessons learned. *J. Control Release* **160**, 117–134 (2012). 22484195 10.1016/j.jconrel.2012.03.020

[545] Silverman, J. A. & Deitcher, S. R. Marqibo(R) (vincristine sulfate liposome injection) improves the pharmacokinetics and pharmacodynamics of vincristine. *Cancer Chemother. Pharm.* **71**, 555–564 (2013). 10.1007/s00280-012-2042-4 PMC3579462 23212117

[546] Jing, D. et al. Quercetin encapsulated in folic acid-modified liposomes is therapeutic against osteosarcoma by non-covalent binding to the JH2 domain of JAK2 Via the JAK2-STAT3-PDL1. *Pharm. Res.* **182**, 106287 (2022). 10.1016/j.phrs.2022.106287 35671921

[547] Zhang, T. et al. Inhalation treatment of primary lung cancer using liposomal curcumin dry powder inhalers. *Acta Pharm. Sin. B* **8**, 440–448 (2018). 29881683 10.1016/j.apsb.2018.03.004 PMC5989825

[548] Jhaveri, A., Deshpande, P., Pattni, B. & Torchilin, V. Transferrin-targeted, resveratrol-loaded liposomes for the treatment of glioblastoma. *J. Control Release* **277**, 89–101 (2018). 29522834 10.1016/j.jconrel.2018.03.006 PMC5911193

[549] Cheng, C. J., Tietjen, G. T., Saucier-Sawyer, J. K. & Saltzman, W. M. A holistic approach to targeting disease with polymeric nanoparticles. *Nat. Rev. Drug Discov.* **14**, 239–247 (2015). 25598505 10.1038/nrd4503 PMC4451203

[550] Zhang, S., Langer, R. & Traverso, G. Nanoparticulate drug delivery systems targeting inflammation for treatment of inflammatory bowel disease. *Nano Today* **16**, 82–96 (2017). 31186671 10.1016/j.nantod.2017.08.006 PMC6557461

[551] Masood, F. Polymeric nanoparticles for targeted drug delivery system for cancer therapy. *Mater. Sci. Eng. C. Mater. Biol. Appl.* **60**, 569–578 (2016). 26706565 10.1016/j.msec.2015.11.067

[552] Zhao, C. Y., Cheng, R., Yang, Z. & Tian, Z. M. Nanotechnology for cancer therapy based on chemotherapy. *Molecules* **23**, 826 (2018). 10.3390/molecules23040826 PMC6017446 29617302

[553] Xiong, K. et al. Co-delivery of paclitaxel and curcumin by biodegradable polymeric nanoparticles for breast cancer chemotherapy. *Int. J. Pharm.* **589**, 119875 (2020). 32919003 10.1016/j.ijpharm.2020.119875

[554] Xiao, B. et al. Co-delivery of camptothecin and curcumin by cationic polymeric nanoparticles for synergistic colon cancer combination chemotherapy. *J. Mater. Chem. B* **3**, 7724–7733 (2015). 26617985 10.1039/c5tb01245g PMC4662402

[555] Kaur, J. et al. Harnessing amphiphilic polymeric micelles for diagnostic and therapeutic applications: breakthroughs and bottlenecks. *J. Control Release* **334**, 64–95 (2021). 33887283 10.1016/j.jconrel.2021.04.014

[556] Ghezzi, M. et al. Polymeric micelles in drug delivery: an insight of the techniques for their characterization and assessment in biorelevant conditions. *J. Control Release* **332**, 312–336 (2021). 33652113 10.1016/j.jconrel.2021.02.031

[557] Hwang, D., Ramsey, J. D. & Kabanov, A. V. Polymeric micelles for the delivery of poorly soluble drugs: from nanoformulation to clinical approval. *Adv. Drug Deliv. Rev.* **156**, 80–118 (2020). 32980449 10.1016/j.addr.2020.09.009 PMC8173698

[558] Ghosh, B. & Biswas, S. Polymeric micelles in cancer therapy: state of the art. *J. Control Release* **332**, 127–147 (2021). 33609621 10.1016/j.jconrel.2021.02.016

[559] Cabral, H. & Kataoka, K. Progress of drug-loaded polymeric micelles into clinical studies. *J. Control Release* **190**, 465–476 (2014). 24993430 10.1016/j.jconrel.2014.06.042

[560] Gong, C. et al. Improving antiangiogenesis and anti-tumor activity of curcumin by biodegradable polymeric micelles. *Biomaterials* **34**, 1413–1432 (2013). 23164423 10.1016/j.biomaterials.2012.10.068

[561] Yang, X. et al. Curcumin-encapsulated polymeric micelles suppress the development of colon cancer in vitro and in vivo. *Sci. Rep.* **5**, 10322 (2015). 25980982 10.1038/srep10322 PMC4434844

[562] Guo, X. et al. Co-delivery of resveratrol and docetaxel via polymeric micelles to improve the treatment of drug-resistant tumors. *Asian J. Pharm. Sci.* **14**, 78–85 (2019). 32104440 10.1016/j.ajps.2018.03.002 PMC7032195

[563] Cote, B., Carlson, L. J., Rao, D. A. & Alani, A. W. G. Combinatorial resveratrol and quercetin polymeric micelles mitigate doxorubicin induced cardiotoxicity in vitro and in vivo. *J. Control Release* **213**, 128–133 (2015). 26160305 10.1016/j.jconrel.2015.06.040

[564] Caminade, A. M., Yan, D. & Smith, D. K. Dendrimers and hyperbranched polymers. *Chem. Soc. Rev.* **44**, 3870–3873 (2015). 26024369 10.1039/c5cs90049b

[565] Dias, A. P. et al. Dendrimers in the context of nanomedicine. *Int. J. Pharm.* **573**, 118814 (2020). 31759101 10.1016/j.ijpharm.2019.118814

[566] Bober, Z., Bartusik-Aebisher, D. & Aebisher, D. Application of dendrimers in anticancer diagnostics and therapy. *Molecules* **27**, 3237 (2022). 10.3390/molecules27103237 PMC9144149 35630713

[567] Dey, A. D. et al. Dendrimers as nanoscale vectors: unlocking the bars of cancer therapy. *Semin. Cancer Biol.* **86**, 396–419 (2022). 35700939 10.1016/j.semcancer.2022.06.003

[568] Gallien, J. et al. Curcumin loaded dendrimers specifically reduce viability of glioblastoma cell lines. *Molecules* **26**, 6050 (2021). 10.3390/molecules26196050 PMC8512379 34641594

[569] Shen, Z. et al. A self-assembly nanodrug delivery system based on amphiphilic low generations of PAMAM dendrimers-ursolic acid conjugate modified by lactobionic acid for HCC targeting therapy. *Nanomedicine* **14**, 227–236 (2018). 29128661 10.1016/j.nano.2017.10.007

[570] Kalluri, R. & McAndrews, K. M. The role of extracellular vesicles in cancer. *Cell* **186**, 1610–1626 (2023). 37059067 10.1016/j.cell.2023.03.010 PMC10484374

[571] Ming-Kun, C. et al. Engineered extracellular vesicles: a new approach for targeted therapy of tumors and overcoming drug resistance. *Cancer Commun.* **44**, 205–225 (2024). 10.1002/cac2.12518 PMC10876209 38155418

[572] Escude Martinez de Castilla, P. et al. Extracellular vesicles as a drug delivery system: a systematic review of preclinical studies. *Adv. Drug Deliv. Rev.* **175**, 113801 (2021). 34015418 10.1016/j.addr.2021.05.011

[573] Yang, C. et al. Extracellular vesicles and their engineering strategies, delivery systems, and biomedical applications. *J. Control Release* **365**, 1089–1123 (2024). 38065416 10.1016/j.jconrel.2023.11.057

[574] S, E. L. A., Mager, I., Breakefield, X. O. & Wood, M. J. Extracellular vesicles: biology and emerging therapeutic opportunities. *Nat. Rev. Drug Discov.* **12**, 347–357 (2013). 23584393 10.1038/nrd3978

[575] Dai, J. et al. Exosomes: key players in cancer and potential therapeutic strategy. *Signal Transduct. Target Ther.* **5**, 145 (2020). 32759948 10.1038/s41392-020-00261-0 PMC7406508

[576] Cabeza, L. et al. Cancer therapy based on extracellular vesicles as drug delivery vehicles. *J. Control Release* **327**, 296–315 (2020). 32814093 10.1016/j.jconrel.2020.08.018

[577] Yong, T. et al. Extracellular vesicles for tumor targeting delivery based on five features principle. *J. Control Release* **322**, 555–565 (2020). 32246977 10.1016/j.jconrel.2020.03.039

[578] Wang, Z. et al. Extracellular vesicles as an emerging drug delivery system for cancer treatment: Current strategies and recent advances. *Biomed. Pharmacother.* **153**, 113480 (2022). 36076581 10.1016/j.biopha.2022.113480

[579] Sun, H. et al. Pancreatic ductal cell-derived extracellular vesicles are effective drug carriers to enhance paclitaxel’s efficacy in pancreatic cancer cells through clathrin-mediated endocytosis. *Int. J. Mol. Sci*. **23**, 4773 (2022). 10.3390/ijms23094773 PMC9099870 35563165

[580] Melzer, C. et al. Taxol-loaded MSC-derived exosomes provide a therapeutic vehicle to target metastatic breast cancer and other carcinoma cells. *Cancers* **11**, 798 (2019). 10.3390/cancers11060798 PMC6627807 31181850

[581] Wang, P. et al. Exosomes from M1-polarized macrophages enhance paclitaxel antitumor activity by activating macrophages-mediated inflammation. *Theranostics* **9**, 1714–1727 (2019). 31037133 10.7150/thno.30716 PMC6485189

[582] Chen, C. et al. Phospholipid-anchored ligand conjugation on extracellular vesicles for enhanced cancer targeting. *Small* **20**, e2310712 (2024). 38733222 10.1002/smll.202310712

[583] Zhu, Q. et al. Embryonic stem cells-derived exosomes endowed with targeting properties as chemotherapeutics delivery vehicles for glioblastoma therapy. *Adv. Sci.* **6**, 1801899 (2019). 10.1002/advs.201801899 PMC6425428 30937268

[584] Zheng, W. et al. Inhalable CAR-T cell-derived exosomes as paclitaxel carriers for treating lung cancer. *J. Transl. Med.* **21**, 383 (2023). 37308954 10.1186/s12967-023-04206-3 PMC10262566

[585] Yang, Y. et al. Camptothecin delivery via tumor-derived exosome for radiosensitization by cell cycle regulation on patient-derived xenograft mice. *Front. Bioeng. Biotechnol.* **10**, 876641 (2022). 35497339 10.3389/fbioe.2022.876641 PMC9039187

[586] Aqil, F. et al. Exosomes for the enhanced tissue bioavailability and efficacy of curcumin. *AAPS J.* **19**, 1691–1702 (2017). 29047044 10.1208/s12248-017-0154-9

[587] Jia, G. et al. NRP-1 targeted and cargo-loaded exosomes facilitate simultaneous imaging and therapy of glioma in vitro and in vivo. *Biomaterials* **178**, 302–316 (2018). 29982104 10.1016/j.biomaterials.2018.06.029

[588] Deng, C. et al. Exosomes derived from mesenchymal stem cells containing berberine for ulcerative colitis therapy. *J. Colloid Interface Sci.* **671**, 354–373 (2024). 38815372 10.1016/j.jcis.2024.05.162

[589] Salek, A. et al. Enhancement of the in vitro antitumor effects of berberine chloride when encapsulated within small extracellular vesicles. *Pharmaceutics* **14**, 1913 (2022). 10.3390/pharmaceutics14091913 PMC9500604 36145661

[590] Lammers, T., Kiessling, F., Hennink, W. E. & Storm, G. Drug targeting to tumors: principles, pitfalls and (pre-) clinical progress. *J. Control Release* **161**, 175–187 (2012). 21945285 10.1016/j.jconrel.2011.09.063

[591] Moosavian, S. A., Bianconi, V., Pirro, M. & Sahebkar, A. Challenges and pitfalls in the development of liposomal delivery systems for cancer therapy. *Semin. Cancer Biol.* **69**, 337–348 (2021). 31585213 10.1016/j.semcancer.2019.09.025

[592] He, H. et al. Survey of clinical translation of cancer nanomedicines-lessons learned from successes and failures. *Acc. Chem. Res.* **52**, 2445–2461 (2019). 31424909 10.1021/acs.accounts.9b00228

[593] Araújo, F. et al. Functionalized materials for multistage platforms in the oral delivery of biopharmaceuticals. *Prog. Mater. Sci.* **89**, 306–344 (2017).

[594] Chen, T. et al. Natural products for combating multidrug resistance in cancer. *Pharm. Res.* **202**, 107099 (2024). 10.1016/j.phrs.2024.107099 38342327

[595] U. Ferreira, M. J. Natural product-derived compounds for targeting multidrug resistance in cancer and microorganisms. *Int. J. Mol. Sci*. **24**, 14321 (2023). 10.3390/ijms241814321 PMC10531746 37762623

[596] Kumar, A. & Jaitak, V. Natural products as multidrug resistance modulators in cancer. *Eur. J. Med. Chem.* **176**, 268–291 (2019). 31103904 10.1016/j.ejmech.2019.05.027

[597] Zou, J. Y. et al. Natural products reverse cancer multidrug resistance. *Front. Pharm.* **15**, 1348076 (2024). 10.3389/fphar.2024.1348076 PMC10988293 38572428

[598] Talib, W. H. et al. Targeting drug chemo-resistance in cancer using natural products. *Biomedicines* **9**, 1353 (2021). 10.3390/biomedicines9101353 PMC8533186 34680470

[599] Yang, C. et al. Natural products in preventing tumor drug resistance and related signaling pathways. *Molecules* **27**, 3513 (2022). 10.3390/molecules27113513 PMC9181879 35684449

[600] Lu, X. et al. Quercetin reverses docetaxel resistance in prostate cancer via androgen receptor and PI3K/Akt signaling pathways. *Int. J. Biol. Sci.* **16**, 1121–1134 (2020). 32174789 10.7150/ijbs.41686 PMC7053318

[601] Tummala, R., Lou, W., Gao, A. C. & Nadiminty, N. Quercetin targets hnRNPA1 to overcome enzalutamide resistance in prostate cancer cells. *Mol. Cancer Ther.* **16**, 2770–2779 (2017). 28729398 10.1158/1535-7163.MCT-17-0030 PMC5716891

[602] Chen, Z. et al. Reversal effect of quercetin on multidrug resistance via FZD7/beta-catenin pathway in hepatocellular carcinoma cells. *Phytomedicine* **43**, 37–45 (2018). 29747752 10.1016/j.phymed.2018.03.040

[603] Seo, H. S. et al. Apigenin overcomes drug resistance by blocking the signal transducer and activator of transcription 3 signaling in breast cancer cells. *Oncol. Rep.* **38**, 715–724 (2017). 28656316 10.3892/or.2017.5752 PMC5562081

[604] Maashi, M. S. et al. Apigenin alleviates resistance to doxorubicin in breast cancer cells by acting on the JAK/STAT signaling pathway. *Mol. Biol. Rep.* **49**, 8777–8784 (2022). 35804214 10.1007/s11033-022-07727-0

[605] Chen, P. et al. Curcumin overcome primary gefitinib resistance in non-small-cell lung cancer cells through inducing autophagy-related cell death. *J. Exp. Clin. Cancer Res.* **38**, 254 (2019). 31196210 10.1186/s13046-019-1234-8 PMC6567416

[606] Lu, Y. et al. Curcumin may reverse 5-fluorouracil resistance on colonic cancer cells by regulating TET1-NKD-Wnt signal pathway to inhibit the EMT progress. *Biomed. Pharmacother.* **129**, 110381 (2020). 32887024 10.1016/j.biopha.2020.110381

[607] Xu, J. et al. Resveratrol reverses Doxorubicin resistance by inhibiting epithelial-mesenchymal transition (EMT) through modulating PTEN/Akt signaling pathway in gastric cancer. *J. Exp. Clin. Cancer Res.* **36**, 19 (2017). 28126034 10.1186/s13046-016-0487-8 PMC5270306

[608] Wang, W. et al. Andrographolide reversed 5-FU resistance in human colorectal cancer by elevating BAX expression. *Biochem. Pharm.* **121**, 8–17 (2016). 27693317 10.1016/j.bcp.2016.09.024

[609] Wang, W. J. et al. Ursolic acid inhibits proliferation and reverses drug resistance of ovarian cancer stem cells by downregulating ABCG2 through suppressing the expression of hypoxia-inducible factor-1alpha in vitro. *Oncol. Rep.* **36**, 428–440 (2016). 27221674 10.3892/or.2016.4813

[610] Li, W. et al. Ursolic acid reduces Adriamycin resistance of human ovarian cancer cells through promoting the HuR translocation from cytoplasm to nucleus. *Environ. Toxicol.* **36**, 267–275 (2021). 33009882 10.1002/tox.23032

[611] Palaskas, N. L. et al. Cardiovascular toxicity of immune therapies for cancer. *BMJ* **385**, e075859 (2024). 38749554 10.1136/bmj-2023-075859

[612] Abdallah, H. M. I. et al. Protective effect of some natural products against chemotherapy-induced toxicity in rats. *Heliyon* **5**, e01590 (2019). 31080906 10.1016/j.heliyon.2019.e01590 PMC6507045

[613] Sahin, T. K., Bilir, B. & Kucuk, O. Modulation of inflammation by phytochemicals to enhance efficacy and reduce toxicity of cancer chemotherapy. *Crit. Rev. Food Sci. Nutr.* **63**, 2494–2508 (2023). 34529530 10.1080/10408398.2021.1976721

[614] Hussain, Y. et al. Curcumin-cisplatin chemotherapy: a novel strategy in promoting chemotherapy efficacy and reducing side effects. *Phytother. Res* **35**, 6514–6529 (2021). 34347326 10.1002/ptr.7225

[615] Fu, B. et al. Multi-component herbal products in the prevention and treatment of chemotherapy-associated toxicity and side effects: a review on experimental and clinical evidences. *Front. Pharm.* **9**, 1394 (2018). 10.3389/fphar.2018.01394 PMC6281965 30555327

[616] Fang, C. Y. et al. Natural products: potential treatments for cisplatin-induced nephrotoxicity. *Acta Pharm. Sin.* **42**, 1951–1969 (2021). 10.1038/s41401-021-00620-9 PMC8633358 33750909

[617] Loren, P. et al. MicroRNAs involved in intrinsic apoptotic pathway during cisplatin-induced nephrotoxicity: potential use of natural products against DDP-induced apoptosis. *Biomolecules* **12**, 1206 (2022). 10.3390/biom12091206 PMC9496062 36139046

[618] Upshaw, J. N. et al. Dexrazoxane to prevent cardiotoxicity in adults treated with anthracyclines: JACC: CardioOncology Controversies in Cardio-Oncology. *JACC CardioOncol.* **6**, 322–324 (2024). 38773999 10.1016/j.jaccao.2024.02.004 PMC11103024

[619] de Baat, E. C. et al. Dexrazoxane for preventing or reducing cardiotoxicity in adults and children with cancer receiving anthracyclines. *Cochrane Database Syst. Rev.* **9**, CD014638 (2022). 36162822 10.1002/14651858.CD014638.pub2 PMC9512638

[620] Benzer, F. et al. Curcumin ameliorates doxorubicin-induced cardiotoxicity by abrogation of inflammation, apoptosis, oxidative DNA damage, and protein oxidation in rats. *J. Biochem. Mol. Toxicol*. **32**, e22030 (2018). 10.1002/jbt.22030 29315967

[621] Bahadir, A. et al. Protective effects of curcumin and beta-carotene on cisplatin-induced cardiotoxicity: an experimental rat model. *Anatol. J. Cardiol.* **19**, 213–221 (2018). 29521316 10.14744/AnatolJCardiol.2018.53059 PMC5864772

[622] Sheu, M. T. et al. Efficacy of antioxidants as a complementary and alternative medicine (CAM) in combination with the chemotherapeutic agent doxorubicin. *Integr. Cancer Ther.* **14**, 184–195 (2015). 25542609 10.1177/1534735414564425

[623] He, H. et al. Epigallocatechin-3-gallate pretreatment alleviates doxorubicin-induced ferroptosis and cardiotoxicity by upregulating AMPKalpha2 and activating adaptive autophagy. *Redox Biol.* **48**, 102185 (2021). 34775319 10.1016/j.redox.2021.102185 PMC8600154

[624] Santana, A. B. et al. Murine response to the opportunistic bacterium *Pseudomonas aeruginosa* infection in gut dysbiosis caused by 5-fluorouracil chemotherapy-induced mucositis. *Life Sci.* **307**, 120890 (2022). 35988752 10.1016/j.lfs.2022.120890

[625] Wang, X. Y. et al. RNA-seq and in vitro experiments reveal the protective effect of curcumin against 5-fluorouracil-induced intestinal mucositis via IL-6/STAT3 signaling pathway. *J. Immunol. Res.* **2021**, 8286189 (2021). 34337082 10.1155/2021/8286189 PMC8318760

[626] Lotfi, M. et al. The protective effects of quercetin nano-emulsion on intestinal mucositis induced by 5-fluorouracil in mice. *Biochem. Biophys. Res. Commun.* **585**, 75–81 (2021). 34800883 10.1016/j.bbrc.2021.11.005

[627] Tang, C., Livingston, M. J., Safirstein, R. & Dong, Z. Cisplatin nephrotoxicity: new insights and therapeutic implications. *Nat. Rev. Nephrol.* **19**, 53–72 (2023). 36229672 10.1038/s41581-022-00631-7

[628] Volarevic, V. et al. Molecular mechanisms of cisplatin-induced nephrotoxicity: a balance on the knife edge between renoprotection and tumor toxicity. *J. Biomed. Sci.* **26**, 25 (2019). 30866950 10.1186/s12929-019-0518-9 PMC6417243

[629] Seker, U. et al. The nephroprotective effect of Quercetin in Cyclophosphamide-induced renal toxicity might be associated with MAPK/ERK and NF-κB signal modulation activity. *Drug Chem. Toxicol.* **47**, 1165–1174 (2024). 38726926 10.1080/01480545.2024.2347541

[630] Borse, V. et al. Epigallocatechin-3-gallate, a prototypic chemopreventative agent for protection against cisplatin-based ototoxicity. *Cell Death Dis.* **8**, e2921 (2017). 28703809 10.1038/cddis.2017.314 PMC5550861

[631] Ortega-Dominguez, B. et al. Curcumin prevents cisplatin-induced renal alterations in mitochondrial bioenergetics and dynamic. *Food Chem. Toxicol.* **107**, 373–385 (2017). 28698153 10.1016/j.fct.2017.07.018

[632] Ali, B. H. et al. Effect of concomitant treatment of curcumin and melatonin on cisplatin-induced nephrotoxicity in rats. *Biomed. Pharmacother.* **131**, 110761 (2020). 33152924 10.1016/j.biopha.2020.110761

[633] Fetoni, A. R. et al. Molecular targets for anticancer redox chemotherapy and cisplatin-induced ototoxicity: the role of curcumin on pSTAT3 and Nrf-2 signalling. *Br. J. Cancer* **113**, 1434–1444 (2015). 26469832 10.1038/bjc.2015.359 PMC4815880

[634] Paciello, F. et al. The dual role of curcumin and ferulic acid in counteracting chemoresistance and cisplatin-induced ototoxicity. *Sci. Rep.* **10**, 1063 (2020). 31974389 10.1038/s41598-020-57965-0 PMC6978317

[635] Ward, R. A. et al. Challenges and opportunities in cancer drug resistance. *Chem. Rev.* **121**, 3297–3351 (2021). 32692162 10.1021/acs.chemrev.0c00383

[636] Beretta, G. L., Gatti, L., Perego, P. & Zaffaroni, N. Camptothecin resistance in cancer: insights into the molecular mechanisms of a DNA-damaging drug. *Curr. Med. Chem.* **20**, 1541–1565 (2013). 23432590 10.2174/0929867311320120006

[637] Januchowski, R., Wojtowicz, K., Andrzejewska, M. & Zabel, M. Expression of MDR1 and MDR3 gene products in paclitaxel-, doxorubicin- and vincristine-resistant cell lines. *Biomed. Pharmacother.* **68**, 111–117 (2014). 24140176 10.1016/j.biopha.2013.09.004

[638] Moon, D. O. Interplay between paclitaxel, gap junctions, and kinases: unraveling mechanisms of action and resistance in cancer therapy. *Mol. Biol. Rep.* **51**, 472 (2024). 38551726 10.1007/s11033-024-09411-x

[639] Rochat, B. Role of cytochrome P450 activity in the fate of anticancer agents and in drug resistance: focus on tamoxifen, paclitaxel and imatinib metabolism. *Clin. Pharmacokinet.* **44**, 349–366 (2005). 15828850 10.2165/00003088-200544040-00002

[640] van Eijk, M. et al. Cytochrome P450 3A4, 3A5, and 2C8 expression in breast, prostate, lung, endometrial, and ovarian tumors: relevance for resistance to taxanes. *Cancer Chemother. Pharm.* **84**, 487–499 (2019). 10.1007/s00280-019-03905-3 PMC6682574 31309254

[641] Hashemi, M. et al. EMT mechanism in breast cancer metastasis and drug resistance: revisiting molecular interactions and biological functions. *Biomed. Pharmacother.* **155**, 113774 (2022). 36271556 10.1016/j.biopha.2022.113774

[642] Shibue, T. & Weinberg, R. A. EMT, CSCs, and drug resistance: the mechanistic link and clinical implications. *Nat. Rev. Clin. Oncol.* **14**, 611–629 (2017). 28397828 10.1038/nrclinonc.2017.44 PMC5720366

[643] Han, M. L. et al. Cathepsin L upregulation-induced EMT phenotype is associated with the acquisition of cisplatin or paclitaxel resistance in A549 cells. *Acta Pharm. Sin.* **37**, 1606–1622 (2016). 10.1038/aps.2016.93 PMC5309731 27840408

[644] Liu, J. et al. Hypoxia induced ferritin light chain (FTL) promoted epithelia mesenchymal transition and chemoresistance of glioma. *J. Exp. Clin. Cancer Res.* **39**, 137 (2020). 32677981 10.1186/s13046-020-01641-8 PMC7364815

[645] Tian, T. et al. Hypoxia-induced intracellular and extracellular heat shock protein gp96 increases paclitaxel-resistance and facilitates immune evasion in breast cancer. *Front. Oncol.* **11**, 784777 (2021). 34988020 10.3389/fonc.2021.784777 PMC8722103

[646] Beinke, S. & Ley, S. C. Functions of NF-kappaB1 and NF-kappaB2 in immune cell biology. *Biochem J.* **382**, 393–409 (2004). 15214841 10.1042/BJ20040544 PMC1133795

[647] Devanaboyina, M. et al. NF-kappaB signaling in tumor pathways focusing on breast and ovarian cancer. *Oncol. Rev.* **16**, 10568 (2022). 36531159 10.3389/or.2022.10568 PMC9756851

[648] Hoarau-Vechot, J. et al. Akt-activated endothelium increases cancer cell proliferation and resistance to treatment in ovarian cancer cell organoids. *Int. J. Mol. Sci.* **23**, 14173 (2022). 10.3390/ijms232214173 PMC9694384 36430649

[649] Luo, X. et al. Enrichment of ovarian cancer stem-like cells is associated with epithelial to mesenchymal transition through an miRNA-activated AKT pathway. *Cell Prolif.* **46**, 436–446 (2013). 23869765 10.1111/cpr.12038 PMC6496712

[650] Sun, T. et al. Inhibition of the notch signaling pathway overcomes resistance of cervical cancer cells to paclitaxel through retardation of the epithelial-mesenchymal transition process. *Environ. Toxicol.* **36**, 1758–1764 (2021). 34048126 10.1002/tox.23296

[651] Park, S. H., Seong, M. A. & Lee, H. Y. p38 MAPK-induced MDM2 degradation confers paclitaxel resistance through p53-mediated regulation of EGFR in human lung cancer cells. *Oncotarget* **7**, 8184–8199 (2016). 26799187 10.18632/oncotarget.6945 PMC4884985

[652] Brosseau, S. et al. YAP/TEAD involvement in resistance to paclitaxel chemotherapy in lung cancer. *Mol. Cell Biochem.* **480**, 231–248 (2025). 38427166 10.1007/s11010-024-04949-7

[653] Zhang, S. F. et al. TXNDC17 promotes paclitaxel resistance via inducing autophagy in ovarian cancer. *Autophagy* **11**, 225–238 (2015). 25607466 10.1080/15548627.2014.998931 PMC4502659

[654] Zhang, X. et al. The role of lncRNA H19 in tumorigenesis and drug resistance of human cancers. *Front. Genet.* **13**, 1005522 (2022). 36246634 10.3389/fgene.2022.1005522 PMC9555214

[655] Han, J. et al. Knockdown of lncRNA H19 restores chemo-sensitivity in paclitaxel-resistant triple-negative breast cancer through triggering apoptosis and regulating Akt signaling pathway. *Toxicol. Appl. Pharm.* **359**, 55–61 (2018). 10.1016/j.taap.2018.09.018 30244121

[656] Bida, O. et al. A novel mitosis-associated lncRNA, MA-linc1, is required for cell cycle progression and sensitizes cancer cells to Paclitaxel. *Oncotarget* **6**, 27880–27890 (2015). 26337085 10.18632/oncotarget.4944 PMC4695032

[657] Chen, Y. M. et al. Linc-ROR induces epithelial-mesenchymal transition and contributes to drug resistance and invasion of breast cancer cells. *Tumour Biol.* **37**, 10861–10870 (2016). 26883251 10.1007/s13277-016-4909-1

[658] Li, X. et al. Knockdown of lncRNA CCAT1 enhances sensitivity of paclitaxel in prostate cancer via regulating miR-24-3p and FSCN1. *Cancer Biol. Ther.* **21**, 452–462 (2020). 32089062 10.1080/15384047.2020.1727700 PMC7515504

[659] Gu, M. et al. LncRNA NONHSAT141924 promotes paclitaxel chemotherapy resistance through p-CREB/Bcl-2 apoptosis signaling pathway in breast cancer. *J. Cancer* **11**, 3645–3654 (2020). 32284761 10.7150/jca.39463 PMC7150466

[660] Liu, X. et al. MicroRNA-34a attenuates paclitaxel resistance in prostate cancer cells via direct suppression of JAG1/Notch1 axis. *Cell Physiol. Biochem.* **50**, 261–276 (2018). 30282072 10.1159/000494004

[661] Zhao, J. et al. STAT3/miR-135b/NF-kappaB axis confers aggressiveness and unfavorable prognosis in non-small-cell lung cancer. *Cell Death Dis.* **12**, 493 (2021). 33990540 10.1038/s41419-021-03773-x PMC8121828

[662] Santos, J. C. et al. Exosome-mediated breast cancer chemoresistance via miR-155 transfer. *Sci. Rep.* **8**, 829 (2018). 29339789 10.1038/s41598-018-19339-5 PMC5770414

[663] Chen, L., Cao, H. & Feng, Y. MiR-199a suppresses prostate cancer paclitaxel resistance by targeting YES1. *World J. Urol.* **36**, 357–365 (2018). 29204706 10.1007/s00345-017-2143-0

[664] Wang, L. et al. Drug resistance in ovarian cancer: from mechanism to clinical trial. *Mol. Cancer* **23**, 66 (2024). 38539161 10.1186/s12943-024-01967-3 PMC10976737

[665] Kim, M. S. et al. Development of exosome-encapsulated paclitaxel to overcome MDR in cancer cells. *Nanomedicine* **12**, 655–664 (2016). 26586551 10.1016/j.nano.2015.10.012 PMC4809755

[666] Nouri-Vaskeh, M. et al. Effect of curcumin supplementation on disease severity in patients with liver cirrhosis: a randomized controlled trial. *Phytother. Res.* **34**, 1446–1454 (2020). 32017253 10.1002/ptr.6620

[667] Choi, Y. H. et al. A randomized, double-blind, placebo-controlled trial to evaluate the role of curcumin in prostate cancer patients with intermittent androgen deprivation. *Prostate* **79**, 614–621 (2019). 30671976 10.1002/pros.23766

[668] Cruz-Correa, M. et al. Efficacy and safety of curcumin in treatment of intestinal adenomas in patients with familial adenomatous polyposis. *Gastroenterology* **155**, 668–673 (2018). 29802852 10.1053/j.gastro.2018.05.031 PMC6120769

[669] Bopanna, S. et al. Risk of colorectal cancer in Asian patients with ulcerative colitis: a systematic review and meta-analysis. *Lancet Gastroenterol. Hepatol.* **2**, 269–276 (2017). 28404156 10.1016/S2468-1253(17)30004-3 PMC5713894

[670] Olen, O. et al. Colorectal cancer in Crohn’s disease: a Scandinavian population-based cohort study. *Lancet Gastroenterol. Hepatol.* **5**, 475–484 (2020). 32066530 10.1016/S2468-1253(20)30005-4

[671] Sadeghi, N., Mansoori, A., Shayesteh, A. & Hashemi, S. J. The effect of curcumin supplementation on clinical outcomes and inflammatory markers in patients with ulcerative colitis. *Phytother. Res.* **34**, 1123–1133 (2020). 31802559 10.1002/ptr.6581

[672] Zheng, X. et al. Single-cell transcriptomic profiling unravels the adenoma-initiation role of protein tyrosine kinases during colorectal tumorigenesis. *Signal Transduct. Target Ther.* **7**, 60 (2022). 35221332 10.1038/s41392-022-00881-8 PMC8882672

[673] Chen, Y. X. et al. Berberine versus placebo for the prevention of recurrence of colorectal adenoma: a multicentre, double-blinded, randomised controlled study. *Lancet Gastroenterol. Hepatol.* **5**, 267–275 (2020). 31926918 10.1016/S2468-1253(19)30409-1

[674] Pintova, S. et al. Genistein combined with FOLFOX or FOLFOX-Bevacizumab for the treatment of metastatic colorectal cancer: phase I/II pilot study. *Cancer Chemother. Pharm.* **84**, 591–598 (2019). 10.1007/s00280-019-03886-3 31203390

[675] Saltz, L. B. et al. Bevacizumab in combination with oxaliplatin-based chemotherapy as first-line therapy in metastatic colorectal cancer: a randomized phase III study. *J. Clin. Oncol.* **26**, 2013–2019 (2008). 18421054 10.1200/JCO.2007.14.9930

[676] Zhao, H. et al. A phase I study of concurrent chemotherapy and thoracic radiotherapy with oral epigallocatechin-3-gallate protection in patients with locally advanced stage III non-small-cell lung cancer. *Radiother. Oncol.* **110**, 132–136 (2014). 24444526 10.1016/j.radonc.2013.10.014

[677] Zhao, H. et al. A prospective, three-arm, randomized trial of EGCG for preventing radiation-induced esophagitis in lung cancer patients receiving radiotherapy. *Radiother. Oncol.* **137**, 186–191 (2019). 30898322 10.1016/j.radonc.2019.02.022

[678] Li, X. et al. Phase II trial of epigallocatechin-3-gallate in acute radiation-induced esophagitis for esophagus cancer. *J. Med. Food* **23**, 43–49 (2020). 31747326 10.1089/jmf.2019.4445

[679] Zhu, W. et al. Epigallocatechin-3-gallate mouthwash protects mucosa from radiation-induced mucositis in head and neck cancer patients: a prospective, non-randomised, phase 1 trial. *Invest. N. Drugs* **38**, 1129–1136 (2020). 10.1007/s10637-019-00871-8 31701429

[680] Zhao, H. et al. Efficacy of epigallocatechin-3-gallate in preventing dermatitis in patients with breast cancer receiving postoperative radiotherapy: a double-blind, placebo-controlled, phase 2 randomized clinical trial. *JAMA Dermatol.* **158**, 779–786 (2022). 35648426 10.1001/jamadermatol.2022.1736 PMC9161122

[681] Lang, A. et al. Curcumin in combination with mesalamine induces remission in patients with mild-to-moderate ulcerative colitis in a randomized controlled trial. *Clin. Gastroenterol. Hepatol.* **13**, 1444–1449.e1441 (2015). 25724700 10.1016/j.cgh.2015.02.019

[682] Epelbaum, R. et al. Curcumin and gemcitabine in patients with advanced pancreatic cancer. *Nutr. Cancer* **62**, 1137–1141 (2010). 21058202 10.1080/01635581.2010.513802

[683] Kanai, M. et al. A phase I/II study of gemcitabine-based chemotherapy plus curcumin for patients with gemcitabine-resistant pancreatic cancer. *Cancer Chemother. Pharm.* **68**, 157–164 (2011). 10.1007/s00280-010-1470-2 20859741

[684] Belcaro, G. et al. A controlled study of a lecithinized delivery system of curcumin (Meriva(R)) to alleviate the adverse effects of cancer treatment. *Phytother. Res.* **28**, 444–450 (2014). 23775598 10.1002/ptr.5014

[685] Bayet-Robert, M. et al. Phase I dose escalation trial of docetaxel plus curcumin in patients with advanced and metastatic breast cancer. *Cancer Biol. Ther.* **9**, 8–14 (2010). 19901561 10.4161/cbt.9.1.10392

[686] Gbolahan, O. B. et al. A phase I evaluation of the effect of curcumin on dose-limiting toxicity and pharmacokinetics of irinotecan in participants with solid tumors. *Clin. Transl. Sci.* **15**, 1304–1315 (2022). 35157783 10.1111/cts.13250 PMC9099132

[687] Saghatelyan, T. et al. Efficacy and safety of curcumin in combination with paclitaxel in patients with advanced, metastatic breast cancer: A comparative, randomized, double-blind, placebo-controlled clinical trial. *Phytomedicine* **70**, 153218 (2020). 32335356 10.1016/j.phymed.2020.153218

[688] Pastorelli, D. et al. Phytosome complex of curcumin as complementary therapy of advanced pancreatic cancer improves safety and efficacy of gemcitabine: results of a prospective phase II trial. *Pharm. Res.* **132**, 72–79 (2018). 10.1016/j.phrs.2018.03.013 29614381

[689] De La Fouchardiere, C. et al. Gemcitabine and paclitaxel versus gemcitabine alone after 5-fluorouracil, oxaliplatin, and irinotecan in metastatic pancreatic adenocarcinoma: a randomized phase III PRODIGE 65-UCGI 36-GEMPAX UNICANCER study. *J. Clin. Oncol.* **42**, 1055–1066 (2024). 38232341 10.1200/JCO.23.00795

[690] Paller, C. J. et al. A phase I study of muscadine grape skin extract in men with biochemically recurrent prostate cancer: safety, tolerability, and dose determination. *Prostate* **75**, 1518–1525 (2015). 26012728 10.1002/pros.23024 PMC4537354

[691] Paller, C. J. et al. Muscadine grape skin extract (MPX) in men with biochemically recurrent prostate cancer: a randomized, multicenter, placebo-controlled clinical trial. *Clin. Cancer Res.* **24**, 306–315 (2018). 29113986 10.1158/1078-0432.CCR-17-1100 PMC5771949

[692] van Die, M. D. et al. A placebo-controlled double-blinded randomized pilot study of combination phytotherapy in biochemically recurrent prostate cancer. *Prostate* **77**, 765–775 (2017). 28181675 10.1002/pros.23317 PMC5444299

[693] Sanoff, H. K. et al. Phase I/II trial of nano-camptothecin CRLX101 with capecitabine and radiotherapy as neoadjuvant treatment for locally advanced rectal cancer. *Nanomedicine* **18**, 189–195 (2019). 30858085 10.1016/j.nano.2019.02.021 PMC7881832

[694] Voss, M. H. et al. A randomized phase II trial of CRLX101 in combination with bevacizumab versus standard of care in patients with advanced renal cell carcinoma. *Ann. Oncol.* **28**, 2754–2760 (2017). 28950297 10.1093/annonc/mdx493

[695] Schmidt, K. T. et al. A single-arm phase II study combining NLG207, a nanoparticle camptothecin, with enzalutamide in advanced metastatic castration-resistant prostate cancer post-enzalutamide. *Oncologist* **27**, 718–e694 (2022). 35640474 10.1093/oncolo/oyac100 PMC9438911

[696] Choueiri, T. K. et al. Neoadjuvant dose-dense methotrexate, vinblastine, doxorubicin, and cisplatin with pegfilgrastim support in muscle-invasive urothelial cancer: pathologic, radiologic, and biomarker correlates. *J. Clin. Oncol.* **32**, 1889–1894 (2014). 24821883 10.1200/JCO.2013.52.4785 PMC7057274

[697] Pfister, C. et al. Dose-dense methotrexate, vinblastine, doxorubicin, and cisplatin or gemcitabine and cisplatin as perioperative chemotherapy for patients with nonmetastatic muscle-invasive bladder cancer: results of the GETUG-AFU V05 VESPER trial. *J. Clin. Oncol.* **40**, 2013–2022 (2022). 35254888 10.1200/JCO.21.02051

[698] Pfister, C. et al. Randomized phase III trial of dose-dense methotrexate, vinblastine, doxorubicin, and cisplatin, or gemcitabine and cisplatin as perioperative chemotherapy for patients with muscle-invasive bladder cancer. Analysis of the GETUG/AFU V05 VESPER trial secondary endpoints: chemotherapy toxicity and pathological responses. *Eur. Urol.* **79**, 214–221 (2021). 32868138 10.1016/j.eururo.2020.08.024

[699] Liu, H. et al. Using low-dose homoharringtonine and cytarabine in combination with granulocyte colony-stimulating factor in a priming induction therapy for acute myeloid leukemia: a retrospective study of 29 cases in China. *Leuk. Lymphoma* **58**, 2758–2761 (2017). 28406352 10.1080/10428194.2017.1312378

[700] Li, J. et al. Homoharringtonine-based induction regimen improved the remission rate and survival rate in Chinese childhood AML: a report from the CCLG-AML 2015 protocol study. *J. Clin. Oncol.* **41**, 4881–4892 (2023). 37531592 10.1200/JCO.22.02836 PMC10617822

[701] Zhang, C. et al. Effectiveness of chemotherapy using bortezomib combined with homoharringtonine and cytarabine in refractory or relapsed acute myeloid leukemia: a phase II, multicenter, prospective clinical trial. *Front. Oncol.* **13**, 1142449 (2023). 37664023 10.3389/fonc.2023.1142449 PMC10472935

[702] Jin, H. et al. Venetoclax combined with azacitidine and homoharringtonine in relapsed/refractory AML: a multicenter, phase 2 trial. *J. Hematol. Oncol.* **16**, 42 (2023). 37120593 10.1186/s13045-023-01437-1 PMC10149010

[703] Yu, S. et al. Sorafenib plus triplet therapy with venetoclax, azacitidine and homoharringtonine for refractory/relapsed acute myeloid leukemia with FLT3-ITD: A multicenter phase 2 study. *J. Intern. Med.* **295**, 216–228 (2024). 37899297 10.1111/joim.13738

[704] Li, J. et al. High efficacy of azacitidine combined with homoharringtonine, idarubicin, and cytarabine in newly diagnosed patients with AML: a single arm, phase 2 trial. *Front. Oncol.* **12**, 1069246 (2022). 36568250 10.3389/fonc.2022.1069246 PMC9773133

[705] Atanasov, A. G. et al. Natural products in drug discovery: advances and opportunities. *Nat. Rev. Drug Discov.* **20**, 200–216 (2021). 33510482 10.1038/s41573-020-00114-z PMC7841765

[706] Zhang, L. et al. The strategies and techniques of drug discovery from natural products. *Pharm. Ther.* **216**, 107686 (2020). 10.1016/j.pharmthera.2020.107686 32961262

[707] Harvey, A. L., Edrada-Ebel, R. & Quinn, R. J. The re-emergence of natural products for drug discovery in the genomics era. *Nat. Rev. Drug Discov.* **14**, 111–129 (2015). 25614221 10.1038/nrd4510

[708] Ancajas, C. M. F., Oyedele, A. S., Butt, C. M. & Walker, A. S. Advances, opportunities, and challenges in methods for interrogating the structure activity relationships of natural products. *Nat. Prod. Rep.* **41**, 1543–1578 (2024). 38912779 10.1039/d4np00009a PMC11484176

[709] Saldivar-Gonzalez, F. I., Aldas-Bulos, V. D., Medina-Franco, J. L. & Plisson, F. Natural product drug discovery in the artificial intelligence era. *Chem. Sci.* **13**, 1526–1546 (2022). 35282622 10.1039/d1sc04471k PMC8827052

[710] Wang, T. et al. Functional metabolomics innovates therapeutic discovery of traditional Chinese medicine derived functional compounds. *Pharm. Ther.* **224**, 107824 (2021). 10.1016/j.pharmthera.2021.107824 33667524

[711] Patil, V. M. & Masand, N. Natural product databases and tools for anti-cancer drug discovery. *Mini Rev. Med Chem.* **21**, 2764–2777 (2021). 32682374 10.2174/1389557520666200719014828

[712] Rutz, A. et al. The LOTUS initiative for open knowledge management in natural products research. *eLife* **11**, e70780 (2022). 10.7554/eLife.70780 PMC9135406 35616633

[713] Zeng, X. et al. NPASS: natural product activity and species source database for natural product research, discovery and tool development. *Nucleic Acids Res.* **46**, D1217–D1222 (2018). 29106619 10.1093/nar/gkx1026 PMC5753227

[714] Banerjee, P. et al. Super Natural II–a database of natural products. *Nucleic Acids Res.* **43**, D935–D939 (2015). 25300487 10.1093/nar/gku886 PMC4384003

[715] Ru, J. et al. TCMSP: a database of systems pharmacology for drug discovery from herbal medicines. *J. Cheminform.* **6**, 13 (2014). 24735618 10.1186/1758-2946-6-13 PMC4001360

[716] Xue, R. et al. TCMID: traditional Chinese medicine integrative database for herb molecular mechanism analysis. *Nucleic Acids Res.* **41**, D1089–D1095 (2013). 23203875 10.1093/nar/gks1100 PMC3531123

[717] Wu, Y. et al. SymMap: an integrative database of traditional Chinese medicine enhanced by symptom mapping. *Nucleic Acids Res.* **47**, D1110–D1117 (2019). 30380087 10.1093/nar/gky1021 PMC6323958

[718] Kong, X. et al. BATMAN-TCM 2.0: an enhanced integrative database for known and predicted interactions between traditional Chinese medicine ingredients and target proteins. *Nucleic Acids Res.* **52**, D1110–D1120 (2024). 37904598 10.1093/nar/gkad926 PMC10767940

[719] Editorial: ChemSpider—a tool for natural products research. *Nat. Prod. Rep.* **32**, 1163–1164 (2015). 10.1039/c5np90022k 26155872

[720] Kim, S. et al. PubChem 2023 update. *Nucleic Acids Res.* **51**, D1373–D1380 (2023). 36305812 10.1093/nar/gkac956 PMC9825602

[721] Sorokina, M. & Steinbeck, C. Review on natural products databases: where to find data in 2020. *J. Cheminform.* **12**, 20 (2020). 33431011 10.1186/s13321-020-00424-9 PMC7118820

[722] van Santen, J. A., Kautsar, S. A., Medema, M. H. & Linington, R. G. Microbial natural product databases: moving forward in the multi-omics era. *Nat. Prod. Rep.* **38**, 264–278 (2021). 32856641 10.1039/d0np00053a PMC7864863

[723] Wilkinson, M. D. et al. The FAIR guiding principles for scientific data management and stewardship. *Sci. Data* **3**, 160018 (2016). 26978244 10.1038/sdata.2016.18 PMC4792175

[724] Boeckhout, M., Zielhuis, G. A. & Bredenoord, A. L. The FAIR guiding principles for data stewardship: fair enough? *Eur. J. Hum. Genet.* **26**, 931–936 (2018). 29777206 10.1038/s41431-018-0160-0 PMC6018669

[725] Ge, N. et al. Version updates of strategies for drug discovery based on effective constituents of traditional Chinese medicine. *Acupunct. Herb. Med.* **2023**, 158–179 (2023).

[726] Caesar, L. K., Montaser, R., Keller, N. P. & Kelleher, N. L. Metabolomics and genomics in natural products research: complementary tools for targeting new chemical entities. *Nat. Prod. Rep.* **38**, 2041–2065 (2021). 34787623 10.1039/d1np00036e PMC8691422

[727] Chen, C. et al. Applications of multi-omics analysis in human diseases. *MedComm* **4**, e315 (2023). 37533767 10.1002/mco2.315 PMC10390758

[728] Chen, B. et al. Harnessing big ‘omics’ data and AI for drug discovery in hepatocellular carcinoma. *Nat. Rev. Gastroenterol. Hepatol.* **17**, 238–251 (2020). 31900465 10.1038/s41575-019-0240-9 PMC7401304

[729] Zielinski, J. M., Luke, J. J., Guglietta, S. & Krieg, C. High throughput multi-omics approaches for clinical trial evaluation and drug discovery. *Front. Immunol.* **12**, 590742 (2021). 33868223 10.3389/fimmu.2021.590742 PMC8044891

[730] Wolfender, J. L., Litaudon, M., Touboul, D. & Queiroz, E. F. Innovative omics-based approaches for prioritisation and targeted isolation of natural products—new strategies for drug discovery. *Nat. Prod. Rep.* **36**, 855–868 (2019). 31073562 10.1039/c9np00004f

[731] Li, D. & Gaquerel, E. Next-generation mass spectrometry metabolomics revives the functional analysis of plant metabolic diversity. *Annu. Rev. Plant Biol.* **72**, 867–891 (2021). 33781077 10.1146/annurev-arplant-071720-114836

[732] Luo, F., Yu, Z., Zhou, Q. & Huang, A. Multi-Omics-based discovery of plant signaling molecules. *Metabolites* **12**, 76 (2022). 10.3390/metabo12010076 PMC8777911 35050197

[733] Li, J. X. et al. Metabolomics and integrated network pharmacology analysis reveal Tricin as the active anti-cancer component of Weijing decoction by suppression of PRKCA and sphingolipid signaling. *Pharm. Res.* **171**, 105574 (2021). 10.1016/j.phrs.2021.105574 34419228

[734] Hao, X. et al. Network pharmacology research and dual-omic analyses reveal the molecular mechanism of natural product nodosin inhibiting muscle-invasive bladder cancer in vitro and in vivo. *J. Nat. Prod.* **85**, 2006–2017 (2022). 35976233 10.1021/acs.jnatprod.2c00400

[735] Han, Y. et al. Chinmedomics, a new strategy for evaluating the therapeutic efficacy of herbal medicines. *Pharm. Ther.* **216**, 107680 (2020). 10.1016/j.pharmthera.2020.107680 PMC7500400 32956722

[736] Ren, J. L. et al. Efficacy evaluation, active ingredients, and multitarget exploration of herbal medicine. *Trends Endocrinol. Metab.* **34**, 146–157 (2023). 36710216 10.1016/j.tem.2023.01.005

[737] Wang, X. J. et al. Novel applications of mass spectrometry-based metabolomics in herbal medicines and its active ingredients: current evidence. *Mass Spectrom. Rev.* **38**, 380–402 (2019). 30817039 10.1002/mas.21589

[738] Wang, M. et al. Chinmedomics: a potent tool for the evaluation of traditional Chinese medicine efficacy and identification of its active components. *Chin. Med.* **19**, 47 (2024). 38481256 10.1186/s13020-024-00917-x PMC10935806

[739] Yan, Y. et al. Screening the effective components of Suanzaoren decoction on the treatment of chronic restraint stress induced anxiety-like mice by integrated chinmedomics and network pharmacology. *Phytomedicine* **115**, 154853 (2023). 37156059 10.1016/j.phymed.2023.154853

[740] Kong, L. et al. Chinmedomics strategy for elucidating the pharmacological effects and discovering bioactive compounds from keluoxin against diabetic retinopathy. *Front. Pharm.* **13**, 728256 (2022). 10.3389/fphar.2022.728256 PMC9008273 35431942

[741] Li, X. N. et al. Screening the active compounds of Phellodendri Amurensis cortex for treating prostate cancer by high-throughput chinmedomics. *Sci. Rep.* **7**, 46234 (2017). 28383015 10.1038/srep46234 PMC5382783

[742] Wang, W. & Krishnan, E. Big data and clinicians: a review on the state of the science. *JMIR Med. Inf.* **2**, e1 (2014). 10.2196/medinform.2913 PMC4288113 25600256

[743] Chunarkar-Patil, P. et al. Anticancer drug discovery based on natural products: from computational approaches to clinical studies. *Biomedicines* **12**, 201 (2024). 10.3390/biomedicines12010201 PMC10813144 38255306

[744] Mullowney, M. W. et al. Artificial intelligence for natural product drug discovery. *Nat. Rev. Drug Discov.* **22**, 895–916 (2023). 37697042 10.1038/s41573-023-00774-7 PMC13118512

[745] Li, G. et al. Artificial intelligence-guided discovery of anticancer lead compounds from plants and associated microorganisms. *Trends Cancer* **8**, 65–80 (2022). 34750090 10.1016/j.trecan.2021.10.002

[746] Yang, X. et al. Concepts of artificial intelligence for computer-assisted drug discovery. *Chem. Rev.* **119**, 10520–10594 (2019). 31294972 10.1021/acs.chemrev.8b00728

[747] Dara, S. et al. Machine learning in drug discovery: a review. *Artif. Intell. Rev.* **55**, 1947–1999 (2022). 34393317 10.1007/s10462-021-10058-4 PMC8356896

[748] Tran, K. A. et al. Deep learning in cancer diagnosis, prognosis and treatment selection. *Genome Med.* **13**, 152 (2021). 34579788 10.1186/s13073-021-00968-x PMC8477474

[749] Li, G. H. & Huang, J. F. CDRUG: a web server for predicting anticancer activity of chemical compounds. *Bioinformatics* **28**, 3334–3335 (2012). 23080119 10.1093/bioinformatics/bts625

[750] Dai, S. X. et al. In silico identification of anti-cancer compounds and plants from traditional Chinese medicine database. *Sci. Rep.* **6**, 25462 (2016). 27145869 10.1038/srep25462 PMC4857115

[751] Rayan, A., Raiyn, J. & Falah, M. Nature is the best source of anticancer drugs: Indexing natural products for their anticancer bioactivity. *PLoS ONE* **12**, e0187925 (2017). 29121120 10.1371/journal.pone.0187925 PMC5679595

[752] Nguyen, L. et al. iANP-EC: identifying anticancer natural products using ensemble learning incorporated with evolutionary computation. *J. Chem. Inf. Model* **62**, 5080–5089 (2022). 35157472 10.1021/acs.jcim.1c00920

[753] Ouyang, B. et al. AI-powered omics-based drug pair discovery for pyroptosis therapy targeting triple-negative breast cancer. *Nat. Commun.* **15**, 7560 (2024). 39215014 10.1038/s41467-024-51980-9 PMC11364624

[754] Odenkirk, M. T., Reif, D. M. & Baker, E. S. Multiomic big data analysis challenges: increasing confidence in the interpretation of artificial intelligence assessments. *Anal. Chem.* **93**, 7763–7773 (2021). 34029068 10.1021/acs.analchem.0c04850 PMC8465926

[755] Chi, J. et al. Artificial intelligence in metabolomics: a current review. *Trends Analyt. Chem.* **178**, 117852 (2024). 10.1016/j.trac.2024.117852 PMC11271759 39071116

[756] Misra, B. B., Langefeld, C. D., Olivier, M. & Cox, L. A. Integrated Omics: tools, advances, and future approaches. *J. Mol. Endocrinol*. **62**, R21–R45 (2018). 10.1530/JME-18-0055 30006342

[757] Jendoubi, T. Approaches to integrating metabolomics and Multi-Omics data: a primer. *Metabolites* **11**,184 (2021). 10.3390/metabo11030184 PMC8003953 33801081

[758] Nussinov, R., Tsai, C. J. & Jang, H. Anticancer drug resistance: an update and perspective. *Drug Resist. Updat* **59**, 100796 (2021). 34953682 10.1016/j.drup.2021.100796 PMC8810687

[759] Wong, A. H. & Deng, C. X. Precision medicine for personalized cancer therapy. *Int. J. Biol. Sci.* **11**, 1410–1412 (2015). 26681920 10.7150/ijbs.14154 PMC4671998

[760] Konig, I. R. et al. What is precision medicine? *Eur. Respir. J.* **50**, 1700391 (2017). 29051268 10.1183/13993003.00391-2017

[761] Zhao, Q. et al. Drug-microbiota interactions: an emerging priority for precision medicine. *Signal Transduct. Target Ther.* **8**, 386 (2023). 37806986 10.1038/s41392-023-01619-w PMC10560686

[762] Duan, X. P. et al. New clinical trial design in precision medicine: discovery, development and direction. *Signal Transduct. Target Ther.* **9**, 57 (2024). 38438349 10.1038/s41392-024-01760-0 PMC10912713

[763] He, X. et al. Artificial intelligence-based multi-omics analysis fuels cancer precision medicine. *Semin. Cancer Biol.* **88**, 187–200 (2023). 36596352 10.1016/j.semcancer.2022.12.009

[764] Adir, O. et al. Integrating artificial intelligence and nanotechnology for precision cancer medicine. *Adv. Mater.* **32**, e1901989 (2020). 31286573 10.1002/adma.201901989 PMC7124889

[765] Cammarota, G. et al. Gut microbiome, big data and machine learning to promote precision medicine for cancer. *Nat. Rev. Gastroenterol. Hepatol.* **17**, 635–648 (2020). 32647386 10.1038/s41575-020-0327-3

[766] Force, U. S. P. S. T. et al. Screening for colorectal cancer: US Preventive Services Task Force Recommendation Statement. *JAMA* **325**, 1965–1977 (2021). 34003218 10.1001/jama.2021.6238

[767] Force, U. S. P. S. T. et al. Aspirin use to prevent cardiovascular disease: US Preventive Services Task Force Recommendation Statement. *JAMA* **327**, 1577–1584 (2022). 35471505 10.1001/jama.2022.4983

[768] Force, U. S. P. S. T. et al. Screening for Colorectal Cancer: US Preventive Services Task Force Recommendation Statement. *JAMA* **315**, 2564–2575 (2016). 27304597 10.1001/jama.2016.5989

[769] Bibbins-Domingo, K., Force U. S. P. S. T. Aspirin Use For The Primary Prevention Of Cardiovascular Disease And Colorectal Cancer: U.S. Preventive Services Task Force Recommendation Statement. *Ann. Intern. Med.* **164**, 836–845 (2016). 27064677 10.7326/M16-0577

[770] Jordan, V. C. & Morrow, M. Tamoxifen, raloxifene, and the prevention of breast cancer. *Endocr. Rev.* **20**, 253–278 (1999). 10368771 10.1210/edrv.20.3.0368

[771] Freedman, A. N. et al. Benefit/risk assessment for breast cancer chemoprevention with raloxifene or tamoxifen for women age 50 years or older. *J. Clin. Oncol.* **29**, 2327–2333 (2011). 21537036 10.1200/JCO.2010.33.0258 PMC3107748

[772] Chen, L., Manautou, J. E., Rasmussen, T. P. & Zhong, X. B. Development of precision medicine approaches based on inter-individual variability of BCRP/ABCG2. *Acta Pharm. Sin. B* **9**, 659–674 (2019). 31384528 10.1016/j.apsb.2019.01.007 PMC6664102

[773] Banik, A., Ghosh, K., Patil, U. K. & Gayen, S. Identification of molecular fingerprints of natural products for the inhibition of breast cancer resistance protein (BCRP). *Phytomedicine* **85**, 153523 (2021). 33662771 10.1016/j.phymed.2021.153523

[774] Raish, M. et al. Effects of Apigenin On Pharmacokinetics Of Dasatinib And Probable Interaction Mechanism. *Molecules* **28**, 1602 (2023). 10.3390/molecules28041602 PMC9964503 36838589

[775] Angelini, A. et al. Modulation of multidrug resistance p-glycoprotein activity by flavonoids and honokiol in human doxorubicin-resistant sarcoma cells (MES-SA/DX-5): implications for natural sedatives as chemosensitizing agents in cancer therapy. *J. Biol. Regul. Homeost. Agents* **24**, 197–205 (2010). 20487633

[776] Smith, A. J. & Humphries, S. E. Cytokine and cytokine receptor gene polymorphisms and their functionality. *Cytokine Growth Factor Rev.* **20**, 43–59 (2009). 19038572 10.1016/j.cytogfr.2008.11.006

[777] Vasilyev, F. F., Silkov, A. N. & Sennikov, S. V. Relationship between interleukin-1 type 1 and 2 receptor gene polymorphisms and the expression level of membrane-bound receptors. *Cell Mol. Immunol.* **12**, 222–230 (2015). 24976267 10.1038/cmi.2014.43 PMC4654293

[778] Zegarra Ruiz, D. F. et al. Microbiota manipulation to increase macrophage IL-10 improves colitis and limits colitis-associated colorectal cancer. *Gut Microbes* **14**, 2119054 (2022). 36062329 10.1080/19490976.2022.2119054 PMC9450902

[779] Yuzhalin, A. The role of interleukin DNA polymorphisms in gastric cancer. *Hum. Immunol.* **72**, 1128–1136 (2011). 21871937 10.1016/j.humimm.2011.08.003

[780] McCann, M. J. et al. The effect of turmeric (*Curcuma longa*) extract on the functionality of the solute carrier protein 22 A4 (SLC22A4) and interleukin-10 (IL-10) variants associated with inflammatory bowel disease. *Nutrients* **6**, 4178–4190 (2014). 25314644 10.3390/nu6104178 PMC4210912

[781] Vogelstein, B. et al. Cancer genome landscapes. *Science* **339**, 1546–1558 (2013). 23539594 10.1126/science.1235122 PMC3749880

[782] Park, E. J. & Pezzuto, J. M. Botanicals in cancer chemoprevention. *Cancer Metastasis Rev.* **21**, 231–255 (2002). 12549763 10.1023/a:1021254725842

[783] Veronesi, U. et al. Prevention of breast cancer with tamoxifen: preliminary findings from the Italian randomised trial among hysterectomised women. Italian Tamoxifen Prevention Study. *Lancet* **352**, 93–97 (1998). 9672273 10.1016/s0140-6736(98)85011-3

[784] Powles, T. et al. Interim analysis of the incidence of breast cancer in the Royal Marsden Hospital tamoxifen randomised chemoprevention trial. *Lancet* **352**, 98–101 (1998). 9672274 10.1016/S0140-6736(98)85012-5

[785] Cuzick, J. et al. First results from the International Breast Cancer Intervention Study (IBIS-I): a randomised prevention trial. *Lancet* **360**, 817–824 (2002). 12243915 10.1016/s0140-6736(02)09962-2

[786] Powles, T. J. et al. Twenty-year follow-up of the Royal Marsden randomized, double-blinded tamoxifen breast cancer prevention trial. *J. Natl Cancer Inst.* **99**, 283–290 (2007). 17312305 10.1093/jnci/djk050

[787] Vogel, V. G. et al. Effects of tamoxifen vs raloxifene on the risk of developing invasive breast cancer and other disease outcomes: the NSABP Study of Tamoxifen and Raloxifene (STAR) P-2 trial. *JAMA* **295**, 2727–2741 (2006). 16754727 10.1001/jama.295.23.joc60074

[788] Askew, D. A., Mickan, S. M., Soyer, H. P. & Wilkinson, D. Effectiveness of 5-fluorouracil treatment for actinic keratosis–a systematic review of randomized controlled trials. *Int. J. Dermatol.* **48**, 453–463 (2009). 19416373 10.1111/j.1365-4632.2009.04045.x

[789] Jeffes, E. W. et al. Photodynamic therapy of actinic keratosis with topical 5-aminolevulinic acid. A pilot dose-ranging study. *Arch. Dermatol* **133**, 727–732 (1997). 9197826

[790] Nelson, C. et al. Phase IV, open-label assessment of the treatment of actinic keratosis with 3.0% diclofenac sodium topical gel (Solaraze). *J. Drugs Dermatol.* **3**, 401–407 (2004). 15303784

[791] Lebwohl, M. et al. Imiquimod 5% cream for the treatment of actinic keratosis: results from two phase III, randomized, double-blind, parallel group, vehicle-controlled trials. *J. Am. Acad. Dermatol.* **50**, 714–721 (2004). 15097955 10.1016/j.jaad.2003.12.010

[792] Kempers, S. et al. Tirbanibulin ointment 1% as a novel treatment for actinic keratosis: phase 1 and 2 results. *J. Drugs Dermatol.* **19**, 1093–1100 (2020). 33196758 10.36849/JDD.2020.5576

[793] Lamm, D. L. BCG in perspective: advances in the treatment of superficial bladder cancer. *Eur. Urol.* **27**(Suppl 1), 2–8 (1995). 7750528 10.1159/000475201

[794] Necchi, A. et al. Pembrolizumab monotherapy for high-risk non-muscle-invasive bladder cancer without carcinoma in situ and unresponsive to BCG (KEYNOTE-057): a single-arm, multicentre, phase 2 trial. *Lancet Oncol.* **25**, 720–730 (2024). 38740030 10.1016/S1470-2045(24)00178-5

[795] Cheng, L., Wang, Y. & Du, J. Human papillomavirus vaccines: an updated review. *Vaccines* **8**, 391 (2020). 10.3390/vaccines8030391 PMC7565290 32708759

[796] Zhai, L. & Tumban, E. Gardasil-9: a global survey of projected efficacy. *Antivir. Res.* **130**, 101–109 (2016). 27040313 10.1016/j.antiviral.2016.03.016

[797] Venters, C., Graham, W. & Cassidy, W. Recombivax-HB: perspectives past, present and future. *Expert Rev. Vaccines* **3**, 119–129 (2004). 15056038 10.1586/14760584.3.2.119

[798] Keating, G. M. & Noble, S. Recombinant hepatitis B vaccine (Engerix-B): a review of its immunogenicity and protective efficacy against hepatitis B. *Drugs* **63**, 1021–1051 (2003). 12699402 10.2165/00003495-200363100-00006

[799] A two-dose hepatitis B vaccine for adults (Heplisav-B). *JAMA* **319**, 822–823 (2018). 10.1001/jama.2018.1097 29486033

[800] Vesikari, T., Langley, J. M., Popovic, V. & Diaz-Mitoma, F. PreHevbrio: the first approved 3-antigen hepatitis B vaccine. *Expert Rev. Vaccines* **22**, 1041–1054 (2023). 37877189 10.1080/14760584.2023.2274482

